# Antibacterial and molecular docking studies of newly synthesized nucleosides and Schiff bases derived from sulfadimidines

**DOI:** 10.1038/s41598-021-97297-1

**Published:** 2021-09-09

**Authors:** Hamada H. Amer, Essam Hassan Eldrehmy, Salama Mostafa Abdel-Hafez, Youssef Saeed Alghamdi, Magdy Yassin Hassan, Saad H. Alotaibi

**Affiliations:** 1 Department of Chemistry, Turabah University College, Taif University, Turabah, 21995 Saudi Arabia; 2 Department of Animal Medicine and Infectious Diseases, Faculty of Veterinary Medicine, University of Sadat City, Sadat City, Egypt; 3 Department of Biology, Turabah University College, Taif University, Turabah, Saudi Arabia; 4 Department of Microbiology, Faculty of Veterinary Medicine, Zagazig University, Zagazig, Egypt; 5 Immunobiology and Immunopharmacology Unit, Animal Reproduction Research Institute, Giza, Egypt; 6 Reproductive Disease Department, Animal Reproduction Research Institute, Giza, Egypt

**Keywords:** Synthetic chemistry methodology, Nuclear chemistry, Organic chemistry, Chemical synthesis, Microbiology, Chemistry

## Abstract

A new series of nucleosides, moieties, and Schiff bases were synthesized from sulfadimidine. Infrared (IR), ^1^HNMR, ^13^C NMR, and mass spectrometry techniques and elemental analysis were employed to elucidate the synthesized compounds. The prepared analogues were purified by different chromatographic techniques (preparative TLC and column chromatography). Molecular docking studies of synthesized compounds 3a, 4b, 6a, and 6e demonstrated the binding mode involved in the active site of DNA gyrase. Finally, all synthesized compounds were tested against selected bacterial strains. The most effective synthesized compounds against *S. aureus* were 3a, 4d, 4b, 3b, 3c, 4c, and 6f, which exhibited inhibition zones of inhibition of 24.33 ± 1.528, 24.67 ± 0.577, 23.67 ± 0.577, 22.33 ± 1.528, 18.67 ± 1.528 and 19.33 ± 0.577, respectively. Notably, the smallest zones were observed for 4a, 6d, 6e and 6g (6.33 ± 1.528, 11.33 ± 1.528, 11.67 ± 1.528 and 14.66 ± 1.155, respectively). Finally, 6b and 6c gave negative zone values. *K. pneumoniae* was treated with the same compounds and the following results were obtained. The most effective compounds were 4d, 4c, 4b and 3c, which showed inhibition zones of 29.67 ± 1.528, 24.67 ± 0.577, 23.67 ± 1.155 and 19.33 ± 1.528, respectively, followed by 4a and 3d (15.33 ± 1.528 for both), while moderate results (13.67 ± 1.155 and 11.33 ± 1.528) were obtained for 6f and 6g, respectively. Finally, 6a, 6b, 6c, 3a, and 3b did not show any inhibition. The most effective compounds observed for the treatment of *E. coli* were 4d, 4b, 4c, 3d, 6e and 6f (inhibition zones of 26.33 ± 0.577, 21.67 ± 1.528, 21.67 ± 1.528, 19.67 ± 1.528, 17.67 ± 1.155 and 16.67 ± 1.155, respectively). Compounds 3b, 3c, 6a, 6c, and 6g gave moderate results (13.67 ± 1.528, 12.67 ± 1.528, 11.33 ± 0.577, 15.33 ± 1.528 and 12.67 ± 1.528, respectively), while 6b showed no effect. The MIC values against *S. aureus* ranged from 50 to 3.125 mg, while those against *E. coli* and *K. pneumoniae* ranged from 50 to 1562 mg. In vitro, the antibacterial effects were promising. Further research is required to study the in vivo antibacterial effects of these compounds and determine therapeutic doses.

## Introduction

A large number of new sugar hydrazones composed of monosaccharides and heterocyclic rings have reportedly been synthesized from quinoxaline derivatives. By testing the synthesized sugar hydrazones against bacteria, it was found that they have very high activity compared to standard drugs^1^. The newly synthesized heterocyclic compounds containing a sulfonamide group showed high activity against microbes in general^2^, and bacteria in particular. Nitrogen-containing heterocyclic compounds associated with sulfonamide groups have received great interest in drug development due to their well-known activity in the fields of pharmaceutical and medicinal chemistry. Heterocyclic sulphonamides have been used as carbonic anhydrase inhibitors^3–5^. Pyrimidines constitute a large group of heterocyclic compounds that have contributed greatly to the discovery of new drugs due to their known biological activity as antibacterial^6^, anti-inflammatory^7^, analgesic^8^ and anticancer drugs^9^. Schiff bases are one of the most extensively used compound types in the fields of organic and medicinal chemistry due to their strong effects as new drugs. Schiff bases derived from heterocyclic aldehydes have shown good anti-fungal and bacterial activities^10^. Nucleosides have been of great importance in medicinal and therapeutic chemistry for a long time due to their anti-viral, anti-parasitic^11^, antibacterial^12^ and anticancer properties^13^. Derivatives of sulfamethazine that are predicted to have high biological activity were designed, synthesized, and tested for their antibacterial activity relative to the antibiotic ofloxacin as the standard antibiotic against *P. aeruginosa* (POA 286), *S. aureus* (ATCC 25923), *B. subtilis* (ATCC 6633), *Salmonella typhi* (ATCC 14028), and *E. coli* (ATCC 12022). The results illustrated that most of the new compounds showed moderate activity against the above-mentioned bacterial strains^14^. Five series of sulfonamide derivatives derived from coumarin were also synthesized, and their biological activities were assessed as clinically important gram-positive and gram-negative antibacterial drugs and the derivatives were also evaluated for their in vitro antibacterial activity against selected clinically important strains, such as *Salmonella typhi*, *S. aureus*, *P. aeruginosa* and *B. cereus*. In addition, they were assessed as antifungal compounds against pathogenic diseases such as those caused by *Curvularia lunata*, *Aspergillus niger*, *Trichoderma viride* and *Aspergillus flavus*. The results showed that the compounds containing sulfonamide groups displayed good activity against the mentioned bacterial and fungal strains^15^. A new series of heterocyclic compounds containing sulfonamides were synthesized and tested for their antibacterial activity. These compounds were found to be highly active (MIC values of 50–3.1 µg/mL) against *P. aeruginosa*, *S. aureus*, *E. coli*, and *B. cereus*; the highest activity was shown against *E. coli*. By reducing their MIC value to 1/4 or 1/32 of their original value, some of the compounds showed 4–32-fold stronger activity relative to the MIC of the reference antibiotic. Additionally, these compounds showed a low degree of cytotoxicity against the VERO, PBM and CEM cell lines^16^. A group of new nucleosides, oxadiazole and arylidine derivatives were synthesized, purified by different chromatographic methods and chemically elucidated by infrared and ^1^H and ^13^C nuclear magnetic resonance spectroscopic techniques. The synthesized compounds were tested against three different strains of bacteria. The results showed high to moderate efficacy against the tested bacterial strains. These studies were compared with theoretical reports, such as those that included molecular docking of the compounds, revealing that of one of these compounds bound to the active site of DNA gyrase^17^. Phthalimide derivatives and their copper complexes have shown antibacterial activity against three bacterial strains, namely, *S. enterica*, *E. faecalis*, and *S. pneumoniae*^18^. Among the most active compounds that reportedly exhibited antibacterial activity were the Schiff bases that were prepared by condensation of sulfonamides such as sulfamethoxypyridazine, which is a compound in the sulfadimidine family with an aldehyde moiety derived from naphthalene, and notably, their complexes were found to be effective antibacterial agents^19^. In this study^14^, Schiff bases were synthesized from 6-(phenyl)-N′-((E)-(phenyl) methylidine)-2-methylpyridine-3-carbohydrate and tested as anti-gram-positive (*S. aureus* and *E. faecalis*) and anti-gram-negative (*E. coli* and *P. aeruginosa*) compounds. The synthesized compounds showed high antibacterial activity at a concentration lower than the standard value^20^. In this work, new nucleosides and Schiff bases were synthesized from sulfadimidine and screened against gram-positive and gram-negative bacteria (Supplementary Information).

## Results and discussion

### Chemistry

**Scheme 1:**
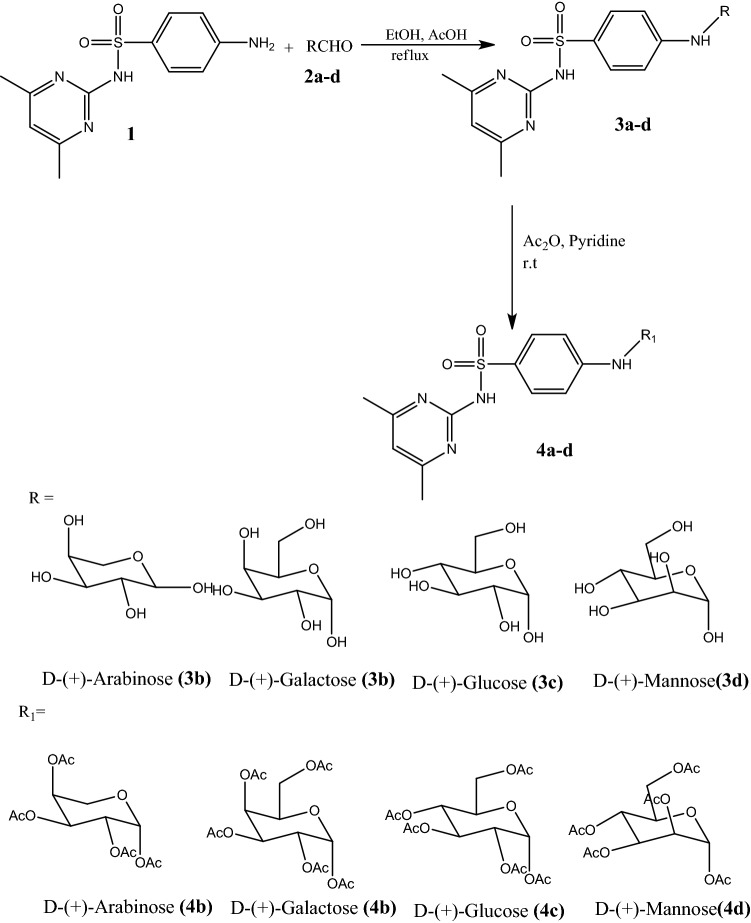
Synthesis of some Nucleoside derivatives.

Sulfadimidine (1) was reacted with different sugar moieties (2a–d) in the presence of a small amount of catalyst (AcOH) using absolute ethanol as the solvent and heated in a condenser to give the corresponding nucleoside derivatives 3a–d in 82–88% yields (Scheme 1). Elucidation of compounds 3a–d by IR spectroscopy showed the disappearance of the (NH_2_) group of sulfadimidine (1) and the appearance of the NH group at 3400 cm^−1^ and the hydroxyl groups of the nucleoside derivatives at 3360 cm^−1^. The ^1^H NMR spectra indicated multiplet peaks at approximately 3.57–4.95 for the CH groups, 3.50 and 3.80 for the OH groups and 8.36 for the NH group in the sugar moieties. The ^13^C NMR spectra showed a peak at 24.24 for the methyl groups of the pyrimidine ring, peaks at approximately 65.00 and 94.00 for the CH group of the sugar moieties, and at 61.93 for the hydroxyl groups of the sugar moieties in compounds 3b, 3c and 3d. The CH aromatic peaks appeared between approximately 110.11 to 168.60. The mass spectra showed molecular ion peaks at 410 [M]^+^, 441 [M+H]^+^, 442 [M+2H]^+^ and 440 [M]^+^ for 3a, 3b, 3c and 3d, respectively. Acetylated nucleosides 4a–d were obtained in 85–93% yields by the acetylation of nucleoside derivatives 3a–d upon reaction with acetic anhydride in dry pyridine at room temperature (Scheme 1). The elucidation of compounds 4a–d by IR spectroscopy showed the disappearance of a peak the 3360 cm^−1^ attributed to the OH group of the nucleosides in 3a–d and the appearance of a peak at 1740 cm^−1^ attributed to the acetyl group (COCH_3_) of the acetylated nucleoside derivatives 4a–d. The ^1^H NMR spectra displayed the disappearance of the peaks from the hydroxyl groups of the sugar moieties between 3.50 and 3.80 and the appearance of multiplet peaks at 2.02 and 2.23 attributed to the acetyl groups (COCH3), multiplet peaks at approximately 3.78–5.36 for the CH groups of the sugar moieties and at approximately 8.45 for the NH group. The 13C NMR spectra showed a peak at 21.00 for the methyl group of the acetyl moiety (COCH3) and at approximately 24.24 for the methyl groups of the pyrimidine ring. The peaks observed between approximately 61.31 to 90.47 were attributed to the CH group in the sugar moieties, and those at 62.41 were caused by the methylene groups in the sugar moieties of compounds 4b, 4c, and 4d. The CH-aromatic peaks appeared at approximately 110.18 to 168.63 and that of the acetyl carbonyl group was at approximately 170.47. The mass spectra showed molecular ion peaks at 539 [M]^+^, 608 [M]^+^, 609 [M+H]^+^ and 608 [M]^+^ for compounds 4a, 4b, 4c and 4d, respectively. Among the nucleoside derivatives, two isomers, E and Z, were present.

**Scheme 2:**
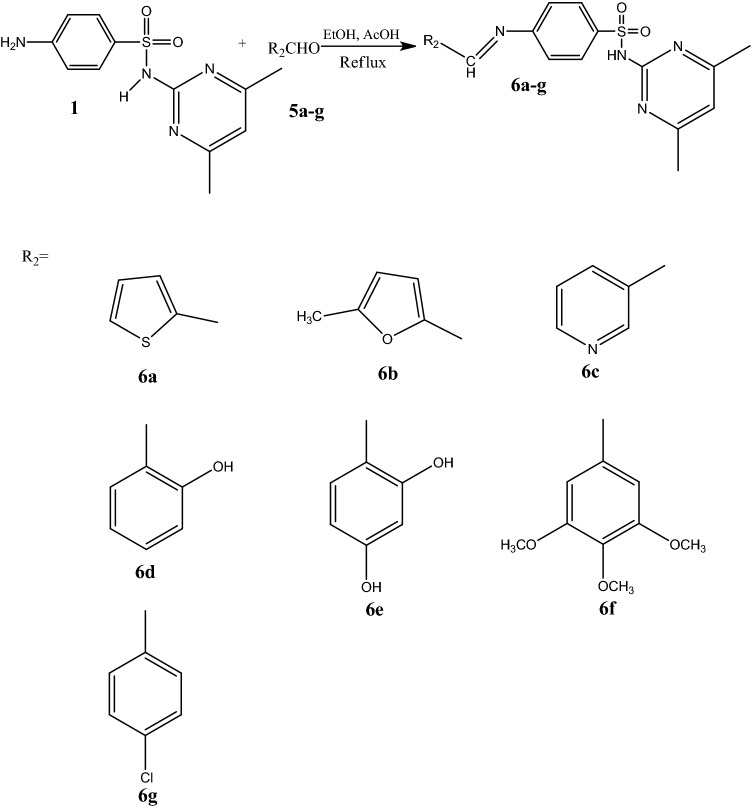
Synthesis of some Schiff bases.

Sulfadimidine (1) was reacted with different aromatic aldehydes 5a–g using absolute ethanol as the solvent in the presence of small amounts of catalyst (AcOH) and heated using a condenser to give the corresponding Schiff base derivatives 6a–g in 92–96% yields (Scheme 2). Elucidation of compounds 6a–g by IR spectroscopy showed the disappearance of the NH_2_ group of sulfadimidine (1) and the appearance of the peak attributed to the NH group at 3370 cm^−1^ and hydroxyl groups of certain Schiff bases at 3450 cm^−1^. The 1H NMR spectra displayed a singlet peak at 2.35 attributed to the methyl groups on the pyrimidine ring, a peak between approximately 5.43–5.76 for the OH groups of the Schiff bases, and multiplet peaks between approximately 6.56–8.50 for Ar-CH and at 9.53 for the NH group. The 13C NMR spectra showed a peak at 22.86 for the methyl groups on the pyrimidine ring and at approximately 54.43 and 61.21 for the methoxy groups (OCH_3_). The peaks between approximately 104.03–168.58 were attributed to the CH aromatics, and those at 163.22 were attributed to CH=N groups. The mass spectra showed molecular ion peaks at 372 [M]^+^, 370 [M]^+^, 367 [M]^+^, 382 [M]^+^, 299 [M+H]^+^, 458 [M+2H]^+^, and 400 [M]^+^ for compounds 6a, 6b, 6c, 6d, 6e, 6f and 6g, respectively.

### Docking studies

**Figure 1:**
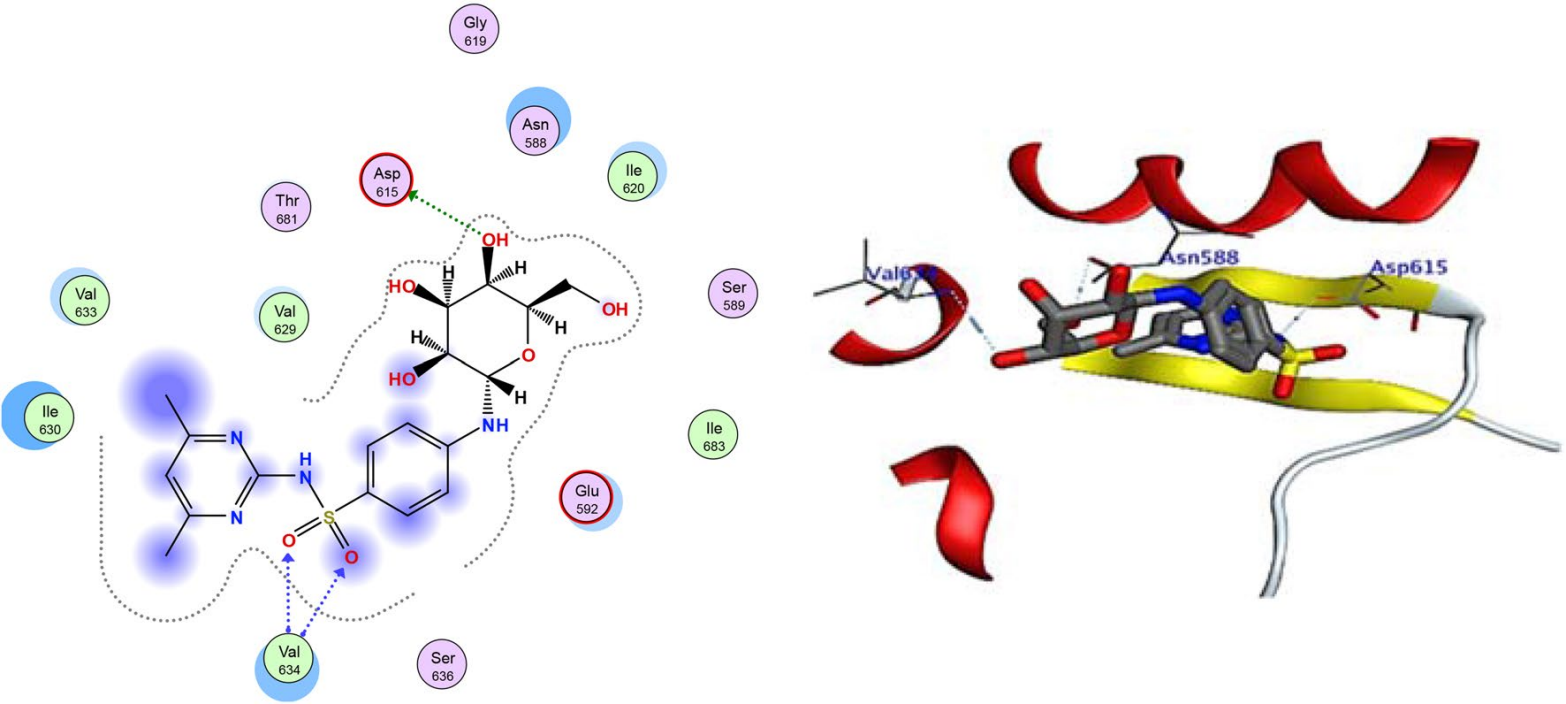
The 2D and 3D binding modes of 3a in the active site of DNA gyrase. H-bonds are represented by blue dashed lines.

**Figure 2:**
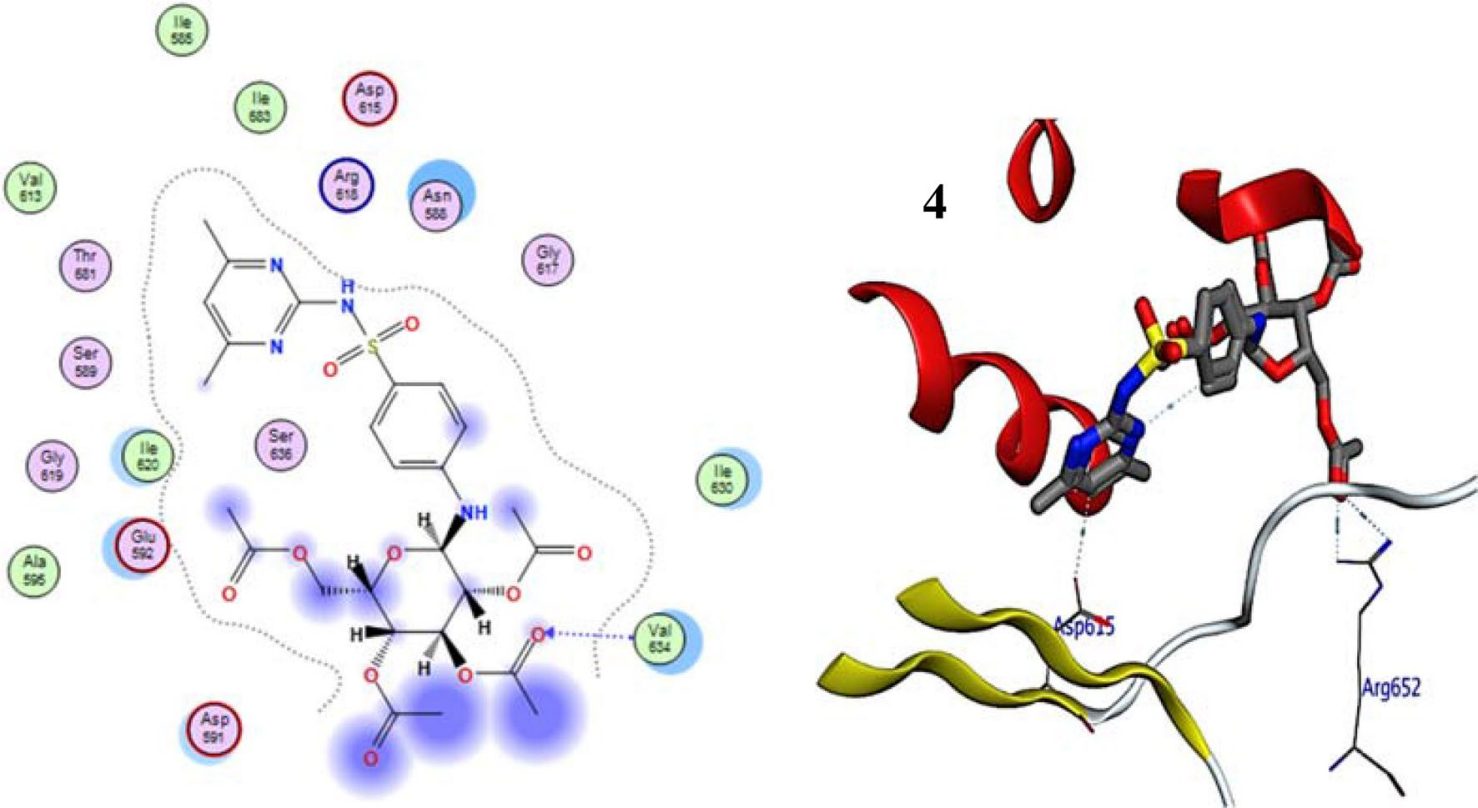
The 2D and 3D binding modes of 4b in the active site of DNA gyrase. H-bonds are represented by blue dashed lines.

**Figure 3:**
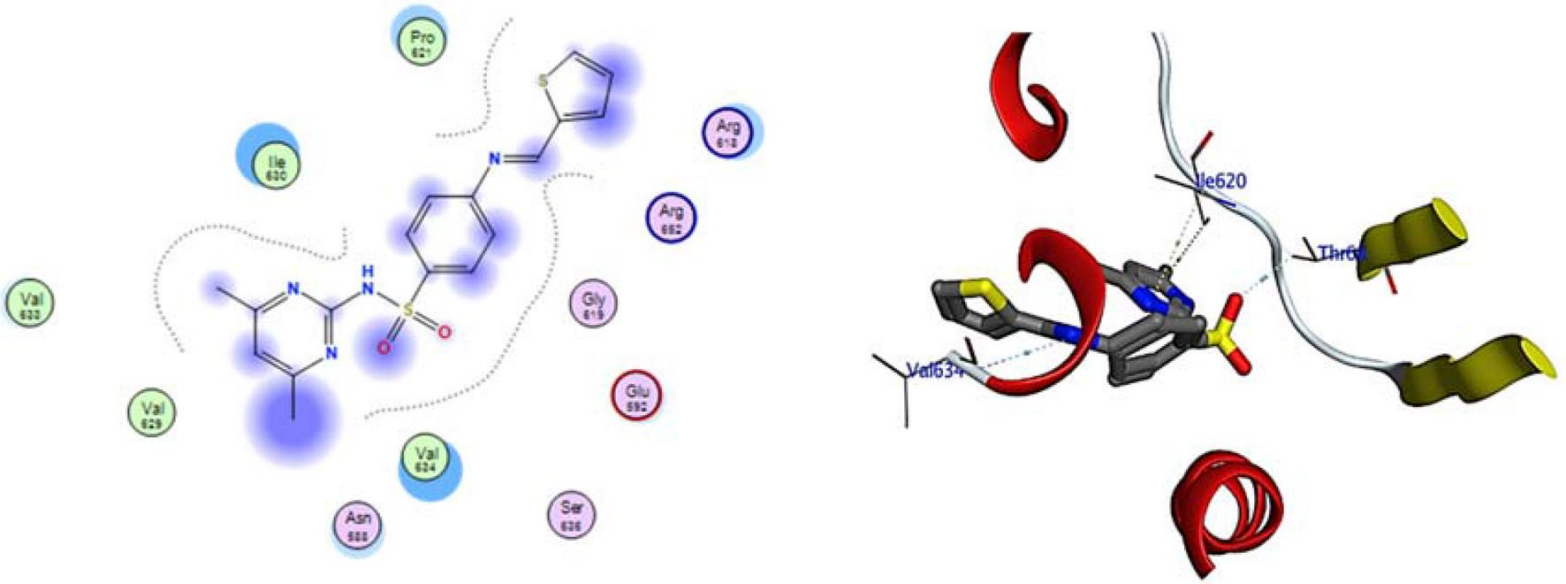
The 2D and 3D binding modes of 6a in the active site of DNA gyrase. H-bonds are represented by blue dashed lines.

**Figure 4:**
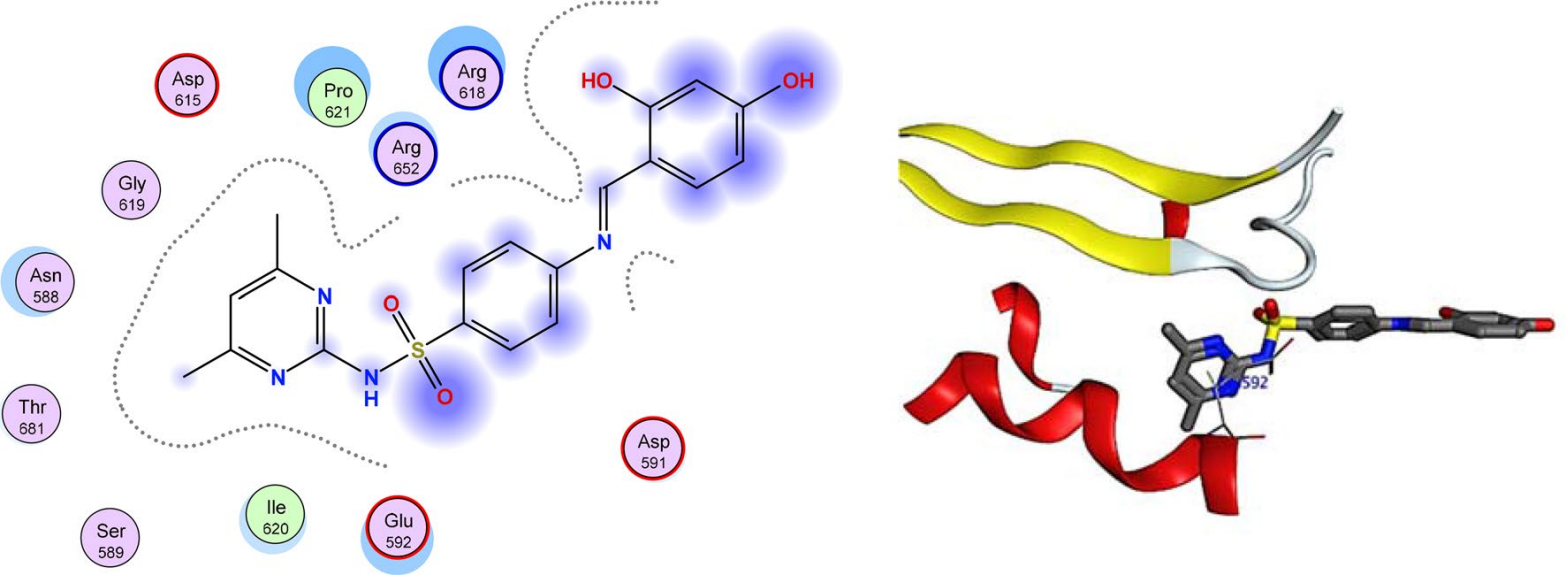
The 2D and 3D binding modes of 6e in the active site of DNA gyrase. H-bonds are represented by blue dashed lines.

The docking study used DNA gyrase B PDB ID: 4uro 14 to examine the mode of action of the small molecule compounds as antimicrobial agents. The ligand–protein interaction behaviours were estimated based on the docking score function, as implemented in the Molecular Operating Environment (MOE) 2015.10 package. All calculations of the docking experiment against the crystal structure (PDB: 4uro) are presented in Table 4. Bacterial DNA gyrase plays a vital role during the investigation of antibacterial agents, as it breaks double-stranded DNA by catalysing negative supercoiling, which is essential for DNA replication, transcription, and recombination15. Analysis of the co-crystallized DNA-gyrase cleavage complex with novobiocin, which is an effective antibacterial agent that cleaves DNA by restricting the ATPase binding site including the vital naked peptidoglycan of the cell wall (Ser55, Ala64, Asn65, Asp89, Thr164, Thr173, and Val79), was performed. The inhibitory effects may be attributed to the distinct differences in the structures of the cell walls between gram-negative and gram-positive bacteria. The cell walls of gram-negative bacteria are composed of a thin peptidoglycan layer (7–8 nm) with an additional outer membrane. Gram-positive bacteria have a thick peptidoglycan layer (20–80 nm) outside the cell wall without an outer membrane. Peptidoglycan is a mesh-like polymer consisting of sugars and amino acids. The peptidoglycan layer protects the microorganism against antibacterial agents such as antibiotics, toxins, chemicals, and degradative enzymes^16,17^. All of the compounds were redocked into the active site without a reference inhibitor and the ligands were successfully complexed with the active sites of the enzyme. The extracted docked poses of the ligands were energy-minimized using the molecular mechanics force field Amber12:EHT until a gradient convergence of 0.05 kcal/mol was achieved. The poses with a docking score of Ed.G < − 7 kcal/mol were initially selected. These poses were filtered on the basis of the lowest MOE docking score, which was attributed to those with the smallest root mean square deviation (RMSD) against the reference drugs. The highest MOE scoring function was applied for the tested compounds to evaluate their binding affinities (Table 1). Compound 3a exhibited the highest binding affinity and RMSD to DNA gyrase compared with the other compounds (− 5.99 kcal/mol.), as summarized in Table 1. The highest antimicrobial activity observed for compound 3a may be explained by the strong interaction with the binding site through the formation of H-bonds with important amino acids, namely, two bonds with Asp615 and two with Val634 (Fig. 1). The considerable RMSD value of < 2 was attributed to the high stability of these compounds in the binding site (Table 1). The binding energy for 4e was found to be − 5.77 kcal/mol, and it interacted with the binding pocket by forming 3 H-bonds with Asp615, Asn588, and Val634 (Fig. 2). Compounds 6a and 6e showed the lowest binding energies (− 4.93 and − 4.65 kcal/mol). Additionally, compound 6a formed two H-bonds with Thr171 and Ala64 and compound 6e exhibited a π–H bond with Glu592 (Table 2; Figs. 3 and 4). Based on the different interaction modes of the ligands with the hydrophilic amino acid backbone in the gyrase binding site (Fig. 1), it was postulated that the hydrophilic fragments in the synthesized compounds (Asp and Asn) play important roles on the binding of molecules to DNA gyrase. Furthermore, the inhibitory efficiency of the synthesized compounds against bacterial growth may be attributed to the attack of the naked peptidoglycan in the cell wall. Therefore, these compounds were inferred to have good biological functions.

**Table 1:** Simulated molecular docking energies (kcal/mol) of the tested ligands.

Cpd	E.dG	rmsd_	E_place	E_conf	Eele	E.Int	Eele_MD_	E.Int. _MD_
3a	− 5.99	1.02	− 136.520	− 86.518	− 11.286	− 20.913	− 13.27	− 22.00
3b	− 5.77	1.66	− 298.362	− 106.58	− 9.846	− 10.369	− 11.17	− 12.74
6a	− 4.93	1.52	− 158.321	− 52.361	− 10.685	− 21.604	− 13.685	− 26.02
6e	− 4.65	1.23	− 358.365	− 192.754	− 13.971	− 29.471	− 13.971	− 27.02

*Ed.G.* final free binding energy, *E_conf.* binding energy for the ligand-conformer, *E_place* binding energy of the ligand-receptor, *E.Int.* ligand-receptor affinity interaction energy, *E.ele* ligand-receptor electrostatic interaction, *RMSD* root mean square deviation of the docking pose compared to the co-crystal ligand position, *E.Int*_*MD*_ interaction energy obtained from molecular dynamics, *E.ele* electrostatic interaction obtained from molecular dynamics.

**Table 2:** Key interactions of the newly designed ligands with the active site of DNA gyrase B (PDB ID: 4uro).

Ligand	Receptor		Interaction	Distance	E (kcal/mol)
**Compound 3a**					
O38	Asp	615	H-donor	3.01	− 1.2
O38	Asp	615	H-donor	2.99	− 2.6
O14	Val	634	H-donor	2.89	− 9.5
O15	Val	634	H-donor	3.0	− 8.0
**Compound 4e**					
N16	ASP	615	H-donor	2.47	− 1.1
O52	ASN	588	H-donor	2.90	− 0.7
S38	Val	634	H-acceptor	2.24	− 4.1
**Compound 6a**					
6-ring	THR	171	H-donor	2.87	− 1.4
O10	ALA	64	H-acceptor	2.87	− 0.9
**Compound 6e**					
6-ring	GLU	592	Π-H	2.35	− 4.00

### Antibacterial activity

**Figure 5:**
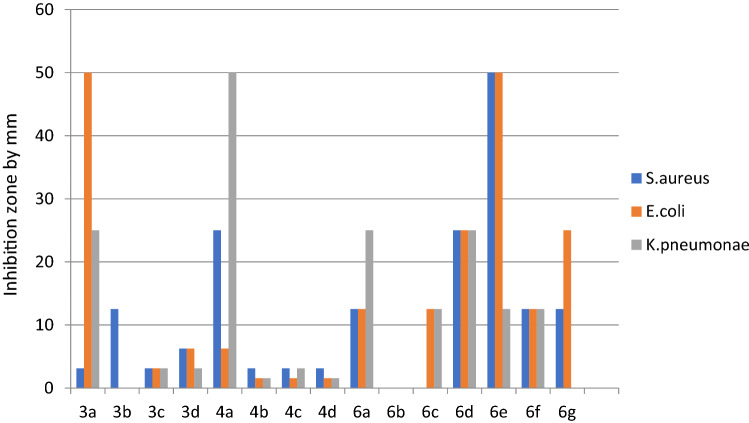
Sensitivity test of the synthesized compounds against three clinical isolates. Inhibition zones are expressed in mm.

**Figure 6:**
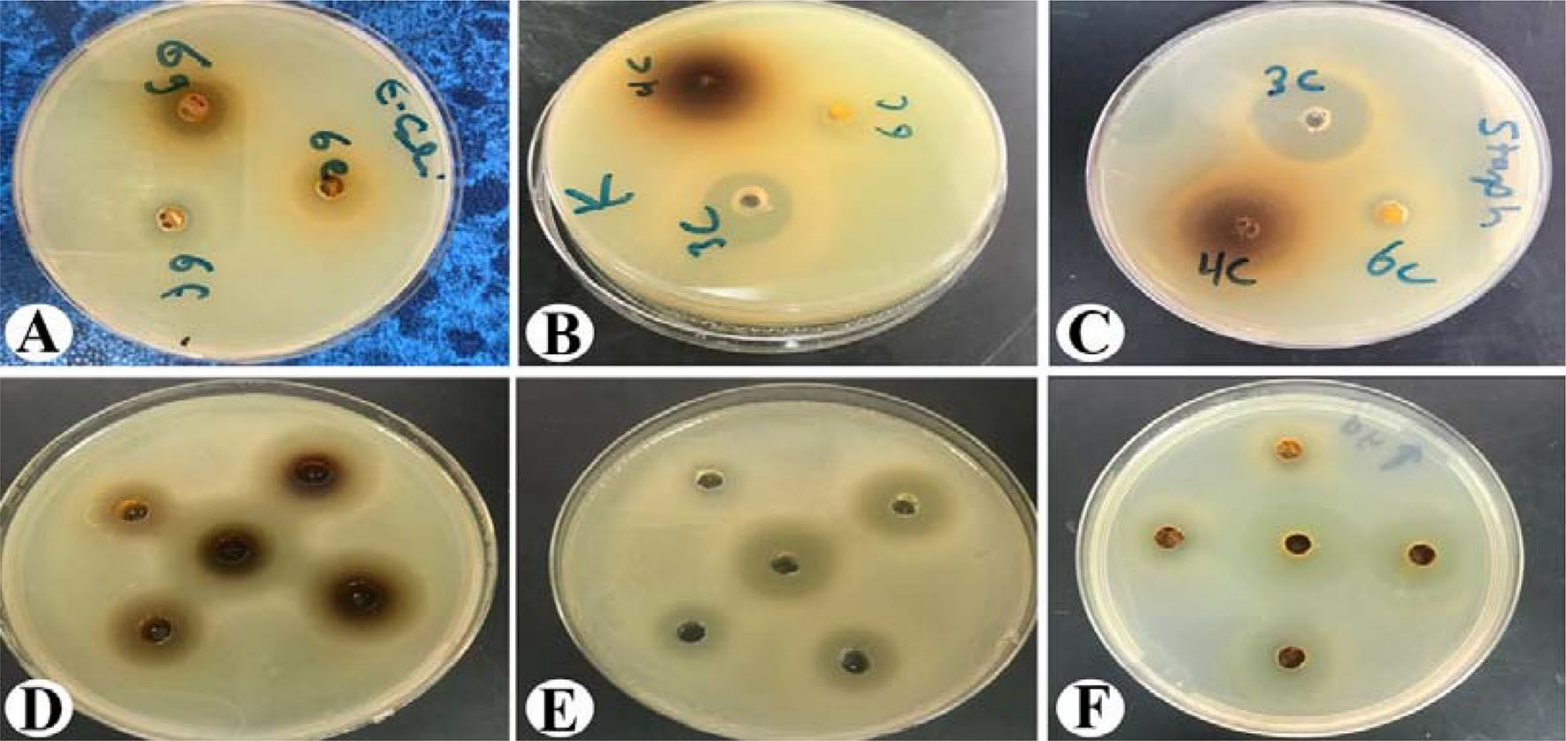
Sensitivity and minimum inhibitory concentration test (MIC) tests of the synthesized compounds against three clinical isolates [(**A**–**C**) sensitivity test; (**D**–**F**) MIC determinations by the agar dilution method].

The results of the sensitivity test are shown in Table 3 and Figs. 5 and 6, which include testing of the synthesized compounds against the three bacterial strains by measuring the sizes of the inhibition zones. The most effective synthesized compounds against *S. aureus* were 3a, 4d, 4b, 3b, 3c, 4c, and 6f Moreover, the smallest zones were observed with 4a, 6d, 6e, and 6g. Finally, 6b and 6c gave negative zone values*. K. pneumoniae* was tested with the same compounds and displayed the following results. The most effective compounds were 4d, 4c, 4b, and 3c, followed by 4a and 3d, and moderate results were obtained for 6f and 6g. Finally, compounds 6a, 6b, 6c, 3a, and 3b showed no inhibition. The most effective compounds observed against *E. coli* were 4d, 4b, 4c, 3d, 6e, and 6f, followed by moderate results for 3b, 3c, 6a, 6c, and 6g. Finally, 6b showed no effect at all. The inhibition zones with ciprofloxacin were all positive. The zones against *S. aureus* showed a value of 21.22 ± 1.55 mm, while the zones against *K. pneumoniae* were 19.22 ± 1.23 mm and those with *E. coli* were 18.52 ± 1.44 mm.

**Table 3:** Antibacterial activity of the tested chemical substances.

Inhibition zone by mm			
Substance code	*S. aureus*	*K. pneumoniae*	*E. coli*
3a	24.33 ± 1.528	nz	11.33 ± 1.528
3b	22.67 ± 1.155	nz	13.67 ± 1.528
3c	22.33 ± 1.528	19.33 ± 1.528	12.67 ± 1.528
3d	18.44 ± 1.432	15.33 ± 1.528	19.67 ± 1.528
4a	6.33 ± 1.528	15.33 ± 1.528	16.33 ± 0.577
4b	23.67 ± 0.577	23.67 ± 1.155	21.67 ± 1.528
4c	18.67 ± 1.528	24.67 ± 0.577	21.67 ± 1.528
4d	24.67 ± 0.577	29.67 ± 1.528	26.33 ± 0.577
6a	15.33 ± 0.577	nz	11.33 ± 0.577
6b	nz	nz	nz
6c	6.66 ± 2.082	nz	15.33 ± 1.528
6d	11.33 ± 1.528	nz	12.67 ± 1.528
6e	11.67 ± 1.528	8.44 ± 1.144	17.67 ± 1.155
6f	19.33 ± 0.577	13.67 ± 1.155	16.67 ± 1.155
6g	14.66 ± 1.155	11.33 ± 1.528	12.67 ± 1.528
Positive control (Ciprofloxacin)	21.22 ± 1.55	19.22 ± 1.23	18.52 ± 1.44
Negative control (DMSO)	NZ	NZ	NZ

Inhibition zones are be expressed as the mean ± SD (mm). *S. aureus*: *Staphylococcus aureus*, *K. pneumoniae*: *Klebsiella pneumoniae* and *E. coli*: *Escherichia coli*. Nz: no zone.

An MIC test was included in this study to determine the lowest concentration of compound that give the lowest inhibition zone using the agar well diffusion test. The results showed that the highest dilutions for the treatment of *S. aureus* were obtained for 3c, 4b, 4c, and 4d, followed by 3d and 6g. Compounds 4a, 6e, and 6f gave the lowest values, and finally, 3a, 6b, 6c showed no inhibition zones at all. Compounds 4b, 3c, 3d, 3a, and 6e showed the best activity against *E. coli*, followed by 6a, 6c, 6f, and 6g, while 6d and 3a gave the lowest dilution values, and 3b, 4c, 4d, and 6b gave negative results. Finally, the reported results against *K. pneumoniae* showed that compounds 4b, 3d, and 3c gave the best results, followed by 6f and 6g. Notably, 4a gave the smallest result, and 3a, 3b, 4c, 4d, 6a, 6b, 6c, 6d, and 6e gave negative results (Table 4; Fig. 5). The MIC values of the sulfonamide derivatives against the *S. aureus* isolate were also evaluated. The MIC values ranged from 50 to 3.125 mg. However, the values ranged from 50 to 1.562 mg for *E. coli* and *K. pneumoniae*^21,22^. In this study, the synthesized sulfadimidine derivatives were tested for their antibacterial activity to develop lead compounds for the production of new antibacterial agents. The outcome of our study showed that the sulfadimidine derivatives were effective enough in comparison to previously reported agents^22^. It was previously reported that sulfadimidine showed the highest inhibitory effects against gram-positive bacteria, i.e., *S. aureus*, and gram-negative bacteria, i.e., *E. coli* and *K. pneumoniae*. The antibacterial activity against these three bacterial isolates was attributed to hydrogen bonding between the functional groups, such as hydroxyl, acetyl, and heterocyclic rings, and the bases in the bacterial DNA gyrase as previously discussed by the docking results.

**Table 4:** Minimum inhibitory concentration (MIC) test of the synthesized compounds against the three tested clinical isolates. N, no zone of inhibition was present.

Substance code	MIC minimum inhibitory concentration MIC by mg/mL		
	*S. aureus*	*E. coli*	*K. pneumoniae*
3a	3.125	50	25
3b	12.5	N	N
3c	3.125	3.125	3.125
3d	6.25	6.25	3.125
4a	25	6.25	50
4b	3.125	1.562	1.562
4c	3.125	1.562	3.125
4d	3.125	1.562	1.562
6a	12.5	12.5	25
6b	N	N	N
6c	N	12.5	12.5
6d	25	25	25
6e	50	50	12.5
6f	12.5	12.5	12.5
6g	12.5	25	12.5

## Material and methods

### Chemistry

The melting points were determined using a Kofler block apparatus without correction. The 1H NMR spectra (results in δ) were acquired on a 400 MHz spectrometer at King Abdel-Aziz University, Jeddah, Saudi Arabia, using CDCl_3_ as the solvent and TMS as the internal standard. IR spectra were recorded using KBr disks on an IC50 model FT-IR (Thermo) instrument at the University College of Turbah, Taif University, Turbah, Taif, Saudi Arabia. Mass spectra were obtained using a GC–MS instrument at Taif University, Taif, Saudi Arabia. TLC was performed using aluminium silica gel plates (60 F245) to monitor the progress of the reactions. The antibacterial activities of the synthesized compounds were evaluated at the Biology Laboratory, Department of Biology, Turabah University College, Taif University, Turabah, Saudi Arabia.

### General procedure for the preparation of 4-amino-*N*-(4,6-dimethylpyrimidine-2-yl) benzenesulfonamide nucleosides (3a–d)

To a solution of sulfadimidine derivative 1 (1.39 g, 10 mmol) in absolute ethanol (50 mL), the appropriate sugar derivative (l-(+)-arabinose, d-(+)-galactose, d-(+)-glucose or d-(+)-mannose; 0.75 g, 0.9 g, 0.9 g or 0.9 g (10 mmol each), respectively) and a few drops of acetic acid were added. The mixture was refluxed for 10–14 h. The formed precipitate was filtered off, washed with absolute ethanol, dried, and recrystallized using an ethanol: methanol mixture (1:2) to give nucleosides 3a–d in 82–88% yields.

#### *N*-Arabinfuranosylamino sulphadimidine (3a)

Brown powder, 85% yield, m.p. = 217–219 °C, Rf = 0.50 (7% methanol in CHCl_3_). IR spectra (KBr) (^V^, cm^−1^), 3400 (NH), 3360 (OH), 3055 (Ar–H), 1150, 1305 (O=S=O); ^1^H NMR (400 MHz, DMSO-d6) δ: 2.35 (6H, s, 2CH_3_), 2.85 (1H, d, J = 2.7 Hz, H-1), 3.57 (2H, d, 2xOH,), 3.80 (1H, d, OH), 3.57–4.95 (4H, m, H-2, H-3, H-4, H-5), 6.67 (1H, s, CH-Pyrimidine), 7.17 (2H, dd, J = 7.00 Hz, CH-aromatic), 7.83 (2H, dd, J = 7.3 Hz, CH-aromatic), 8.36 (2H, brs, 2NH); ^13^C NMR (100 MHz, CDCl3): δ: 24.25 (CH_3_), 65.23, 70.25, 73.76, 76.32, 94.00 (CH-Sugar), 110.17, 111.21, 128.22, 130.07, 150.92, 166.48, 168.61 (Ar-CH); MS: m/z (%) 410 (M)^+^. Anal. Calcd for C_17_H_22_N_4_O_6_S: C, 49.75; H, 5.40; N, 13.65. Found C, 49.88; H, 5.62; N, 13.46.

#### *N*-Glactopyranosylamino sulphadimidine (3b)

White powder, 86% yield, m.p. = 244–246 °C, Rf = 0.45 (7% methanol in CHCl_3_). IR spectra (KBr) (^V^, cm^−1^), 3390 (NH), 3320 (OH), 3050 (Ar–H), 1150, 1305 (O=S=O); ^1^H NMR (400 MHz, DMSO-d6) δ: 2.32 (6H, s, 2CH_3_), 3.59 (2H, dd, J = 5.4 Hz, 2xOH,), 3.59 (2H, dd, J = 5.5 Hz, 2xOH), 4.58 (1H, d, J = 3.00 Hz, H-1), 3.57–3.97 (6H, m, H-2, H-3, H-4, H-5, H-6), 6.65 (1H, s, CH-Pyrimidine), 7.15 (2H, dd, J = 7.00 Hz, CH-aromatic), 7.82 (2H, dd, J = 7.3 Hz, CH-aromatic), 8.22 (2H, brs, 2NH)); ^13^C NMR (100 MHz, CDCl3): δ: 24.25 (CH_3_), 61.93 (CH_2_OH of sugar), 71.24, 74.12, 77,45, 82.72, 91.60 (CH-Sugar), 110.17, 111.11, 128.17, 130.07, 150.92, 166.48, 168.61 (Ar-CH); MS: m/z (%) 441 (M+H)^+^. Anal. Calcd for C_18_H_24_N_4_O_7_S: C, 49.08; H, 5.49; N, 12.72. Found C, 48.87; H, 5.15; N, 12.40.

#### *N*-Glucopyranosylamino sulphadimidine (3c)

Pale brown powder, 82% yield, m.p. = 256–258 °C, Rf = 0.40 (7% methanol in CHCl_3_). IR spectra (KBr) (^V^, cm^−1^), 3390 (NH), 3320 (OH), 3050 (Ar–H), 1150, 1305 (O=S=O); ^1^H NMR (400 MHz, DMSO-d6) δ: 2.30 (6H, s, 2CH_3_), 3.58 (2H, dd, J = 5.4 Hz, 2xOH,), 3.60 (2H, dd, J = 5.5 Hz, 2xOH), 4.55 (1H, d, J = 3.00 Hz, H-1), 3.57–3.95 (6H, m, H-2, H-3, H-4, H-5, H-6), 6.62 (1H, s, CH-Pyrimidine), 7.13 (2H, dd, J = 7.00 Hz, CH-aromatic), 7.80 (2H, dd, J = 7.3 Hz, CH-aromatic), 8.18 (2H, brs, 2NH); ^13^C NMR (100 MHz, CDCl3): δ: 24.25 (CH_3_), 61.93 (CH_2_OH of sugar), 71.24, 74.12, 77,45, 82.72, 91.60 (CH-Sugar), 110.17, 111.11, 128.17, 130.07, 150.92, 166.48, 168.61 (Ar-CH); MS: m/z (%) 442 (M+2H)^+^. Anal. Calcd for C_18_H_24_N_4_O_7_S: C, 49.08; H, 5.49; N, 12.72. Found C, 49.32; H, 5.64; N, 12.87.

#### *N*-Mannopyranosylamino sulphadimidine (3d)

White powder, 88% yield, m.p. = 270–272 °C, Rf = 0.45 (7% methanol in CHCl_3_). IR spectra (KBr) (^V^, cm^−1^), 3390 (NH), 3320 (OH), 3050 (Ar–H), 1150, 1305 (O=S=O); ^1^H NMR (400 MHz, DMSO-d6) δ: 2.30 (6H, s, 2CH_3_), 3.53 (2H, dd, J = 5.4 Hz, 2xOH,), 3.58 (2H, dd, J = 5.5 Hz, 2xOH), 4.59 (1H, d, J = 3.00 Hz, H-1), 3.59–3.92 (6H, m, H-2, H-3, H-4, H-5, H-6), 6.66 (1H, s, CH-Pyrimidine), 7.20 (2H, dd, J = 7.00 Hz, CH-aromatic), 7.87 (2H, dd, J = 7.3 Hz, CH-aromatic), 8.25 (2H, brs, 2NH); ^13^C NMR (100 MHz, CDCl3): δ: 24.25 (CH_3_), 61.93 (CH_2_OH of sugar), 71.24, 74.12, 77,45, 82.72, 91.60 (CH-Sugar), 110.17, 111.11, 128.17, 130.07, 150.92, 166.48, 168.61 (Ar-CH); MS: m/z (%) 440 (M)^+^. Anal. Calcd for C_18_H_24_N_4_O_7_S: C, 49.08; H, 5.49; N, 12.72. Found C, 49.55; H, 5.56; N, 12.63.

### General procedure for the synthesis of acetylated nucleosides 4a–d

A solution of nucleoside derivatives 3a–d (0.01 mol) in 15 mL of anhydrous pyridine was treated with acetic anhydride (0.01 mol). The reaction mixture was stirred for 14 h at room temperature and monitored by TLC. Each mixture was poured into 100 g of ice to give a pale yellow precipitate. The resulting precipitates were filtered, washed with water, and dried to produce acetylated nucleosides 4a–d in 85–93% yields.

#### 4-(2, 3, 5-Tri-*O*-acetyl)-*N*-arabinfuranosylamino) sulphadimidine (4a)

Pale yellow powder, 93% yield, m.p. = 265–267 °C, Rf = 0.45 (7% methanol in CHCl_3_). IR spectra (KBr) (^V^, cm^−1^), 3350 (NH), 3050 (Ar–H), 1740 (COCH_3_), 1625 (C=N), 1375 (CH_3_), 1150, 1305 (O=S=O); ^1^H NMR (400 MHz, DMSO-d6) δ: 2.02, 2.11, 2.19 (9H, m, 3× COCH_3_), 2.35 (1H, s, CH_3_), 3.78–5.20 (4H, m, H-2, H-3, H-4, H-5), 5.36 (1H, d, J = 5.5 Hz, H-1), 6.75–7.61 (5H, m, Ar–H), 8.45 (1H, brs, 2NH); ^13^C NMR (100 MHz, CDCl3): δ: 21.00 (3CH_3_ of 3COCH_3_ of sugar), 24.25 (2CH_3_ of pyrimidine ring), 61.31, 68.23, 70.91, 72.79, 90.47 (CH-Sugar), 110.18, 111.21, 128.19, 130.09, 150.96, 166.50, 168.63 (Ar-CH), 170.47 (3C=O of 3COCH3 of sugar); MS: m/z (%) 536 (M)^+^. Anal. Calcd for C_23_H_28_N_4_O_9_S: C, 51.49; H, 5.26; N, 10.44. Found C, 51.67; H, 5.47; N, 10.58.

#### 4-(2, 3, 5, 6-Tetra-*O*-acetyl)-*N*-galactopyranosylamino)- sulphadimidine (4b)

Pale yellow powder, 90% yield, m.p. = 230–232 °C, Rf = 0.45 (7% methanol in CHCl_3_). IR spectra (KBr) (^V^, cm^−1^), 3400 (NH), 3050 (Ar–H), 1735 (COCH_3_), 1375 (CH_3_), 1150, 1305 (O=S=O); ^1^H NMR (400 MHz, DMSO-d6) δ: 2.10, 2.13, 2.18, 2.23 (12H, m, 4xCOCH_3_), 2.34 (CH_3_), 4.22–5.35 (5H, m, H-2, H-3, H-4, H-5, H-6), 5.50 (1H, d, J = 5.5 Hz, H-1), 6.76–7.60 (5H, m, Ar–H), 8.75 (1H, brs, 2NH); ^13^C NMR (100 MHz, CDCl3): δ: 20. 87 (CH_3_ of COCH_3_ of sugar), 21.11 (3CH_3_ of 3COCH_3_ of sugar), 23.80 (2CH_3_ of pyrimidine ring), 62.41(CH_2_), 68.98, 69.43, 73.08, 77.41, 87.86 (CH-Sugar), 110.18, 111.21, 128.19, 130.09, 150.96, 166.50, 168.63 (Ar-CH), 170.24 (4C=O of 4COCH3 of sugar); MS: m/z (%) 608 (M)^+^. Anal. Calcd for C_26_H_32_N_4_O_11_S: C, 54.21; H, 4.71; N, 8.72. Found C, 54.41; H, 4.87; N, 8.45.

#### 4-(2, 3, 5, 6-Tetra-*O*-acetyl)-*N*-glucopyranosylamino) sulphadimidine (4c)

Pale yellow powder, 85% yield, m.p. = 243–245 °C, Rf = 0.45 (7% methanol in CHCl_3_). IR spectra (KBr) (^V^, cm^−1^), 3400 (NH), 3050 (Ar–H), 1735 (COCH_3_), 1375 (CH_3_), 1150, 1305 (O=S=O); ^1^H NMR (400 MHz, DMSO-d6) δ: 2.08, 2.11, 2.17, 2.20 (12H, m, 4xCOCH_3_), 2.35 (CH_3_), 4.24–5.30 (5H, m, H-2, H-3, H-4, H-5, H-6), 5.45 (1H, d, J = 5.5 Hz, H-1), 6.74–7.64 (5H, m, Ar–H), 8.75 (1H, brs, 2NH); ^13^C NMR (100 MHz, CDCl3): δ: 20. 87 (CH_3_ of COCH_3_ of sugar), 21.11 (3CH_3_ of 3COCH_3_ of sugar), 23.80 (2CH_3_ of pyrimidine ring), 62.41(CH_2_), 68.98, 69.43, 73.08, 77.41, 87.86 (CH-Sugar), 110.18, 111.21, 128.19, 130.09, 150.96, 166.50, 168.63 (Ar-CH), 170.24 (4C=O of 4COCH3 of sugar); MS: m/z (%) 609 (M+H)^+^. Anal. Calcd for C_26_H_32_N_4_O_11_S: C, 54.21; H, 4.71; N, 8.72. Found C, 54.48; H, 4.89; N, 8.50.

#### 4-(2, 3, 5, 6-Tetra-*O*-acetyl)-*N*-glucopyranosylamino) sulphadimidine (4d)

Pale yellow powder, 88% yield, m.p. = 270–272 °C, Rf = 0.45 (7% methanol in CHCl_3_). IR spectra (KBr) (^V^, cm^−1^), 3400 (NH), 3050 (Ar–H), 1735 (COCH_3_), 1375 (CH_3_), 1150, 1305 (O=S=O); ^1^H NMR (400 MHz, DMSO-d6) δ: 2.05, 2.10, 2.15, 2.18 (12H, m, 4xCOCH_3_), 2.30 (CH_3_), 4.25–5.32 (5H, m, H-2, H-3, H-4, H-5, H-6), 5.42 (1H, d, J = 5.5 Hz, H-1), 6.74–7.64 (5H, m, Ar–H), 8.85 (1H, brs, 2NH); ^13^CNMR (100 MHz, CDCl3): δ: 20. 87 (CH_3_ of COCH_3_ of sugar), 21.11 (3CH_3_ of 3COCH_3_ of sugar), 23.80 (2CH_3_ of pyrimidine ring), 62.41 (CH_2_), 68.98, 69.43, 73.08, 77.41, 87.86 (CH-Sugar), 110.18, 111.21, 128.19, 130.09, 150.96, 166.50, 168.63 (Ar-CH), 170.24 (4C=O of 4COCH3 of sugar); MS: m/z (%) 608 (M)^+^. Anal. Calcd for C_26_H_32_N_4_O_11_S: C, 54.21; H, 4.71; N, 8.72. Found C, 54.50; H, 4.90; N, 8.47.

### General procedure for the synthesis of arylidene derivatives 6a–g

A solution of 1 (0.01 mol) and the appropriate aromatic aldehyde [thiophene-2-aldehyde, 5-methylfurfural, pyridine-3-carboxaldehyde, salicylaldehyde, 2,4-dihydroxybenzaldehyde, 3,4,5-trimethoxybenzaldehyde or p-chlorobenzaldehyde (0.01 mol)] were dissolved in 50 mL of ethanol. A small amount of acetic acid (1 mL) was added to the reaction mixture as the catalyst, which was refluxed for 10 h. The resulting product was filtered off to afford 6a–g in 92–96% yields.

#### *N*-(4,6-dimethylpyrimidin-2-yl)-4-((thiophen-2-ylmethylene)amino)benzenesulfonamide (6a)

Pale yellow powder, 94% yield, m.p. = 155–157 °C, Rf = 0.45 (7% methanol in CHCl_3_). IR spectra (KBr) (^V^, cm^−1^), 3370 (NH), 3050 (Ar–H), 1150, 1305 (O=S=O); ^1^H NMR (400 MHz, DMSO-d6) δ: 2.35 (6H, s, 2CH_3_), 6.75–7.89 (8H, m, Ar–H), 8.25 (1H, brs, CH), 9.78 (1H, brs, NH); ^13^C NMR (100 MHz, CDCl3): δ: 24.23 (CH_3_), 110.13, 122.62, 127.21, 124.13, 128.63, 130.27, 138.23, 132.43, 151.25, 166.69, 168.57 (Ar-CH), 152.81 (CH=N); MS: m/z (%) 372 (M)^+^. Anal. Calcd for C_17_H_16_N_4_O_2_S_2_: C, 54.82; H, 4.33; N, 15.04. Found C, 54.98; H, 4.56; N, 14.97.

#### *N*-(4,6-dimethylpyrimidin-2-yl)-4-(((5-methylfuran-2-yl)methylene)amino)benzenesulfonamide (6b)

Brown powder, 93% yield, m.p. = 181–183 °C, Rf = 0.45 (7% methanol in CHCl_3_). IR spectra (KBr) (^V^, cm^−1^), 3370 (NH), 3050 (Ar–H), 1150, 1305 (O=S=O); ^1^H NMR (400 MHz, DMSO-d6) δ: 2.12 (3H, s, CH_3_), 2.30 (6H, s, 2CH_3_), 6.65–8.50 (7H, m, Ar–H), 8.88 (1H, brs, CH), 9.86 (1H, brs, NH); ^13^C NMR (100 MHz, CDCl3): δ: 16.50 (CH_3_), 24.25 (2CH_3_), 106.73, 110.11, 122.64, 128.62, 138.26, 147.33, 151.60, 155.65, 166.56, 168.67 (Ar-CH), 144.95 (CH=N);. MS: m/z (%) 370 (M)^+^. Anal. Calcd for C_18_H_18_N_4_O_3_S: C, 58.36; H, 4.90; N, 15.12. Found C, 58.47; H, 4.78; N, 15.43.

#### *N*-(4,6-dimethylpyrimidin-2-yl)-4-((pyridin-3-ylmethylene)amino)benzenesulfonamide (6c)

White powder, 92% yield, m.p. = 167–169 °C, Rf = 0.45 (7% methanol in CHCl_3_). IR spectra (KBr) (^V^, cm^−1^), 3370 (NH), 3050 (Ar–H), 1150, 1305 (O=S=O); ^1^H NMR (400 MHz, DMSO-d6) δ: 2.32 (6H, s, 2CH_3_), 6.72–7.80 (9H, m, Ar–H), 7.95 (1H, brs, CH), 9.53 (1H, brs, NH); ^13^C NMR (100 MHz, CDCl3): δ: 24.25 (2CH_3_), 110.13, 122.61, 123.90, 128.60, 130.44, 133.75, 138.27, 149.04, 151.80, 151.92, 166.66, 168.54 (Ar-CH), 159.78 (CH=N); MS: m/z (%) 367 (M)^+^. Anal. Calcd for C_18_H_17_N_5_O_2_S: C, 58.84; H, 4.66; N, 19.06. Found C, 58.70; H, 4.81; N, 18.97.

#### *N*-(4,6-dimethylpyrimidin-2-yl)-4-((2-hydroxybenzylidene)amino)benzenesulfonamide (6d)

Yellow powder, 96% yield, m.p. = 210–212 °C, Rf = 0.45 (7% methanol in CHCl_3_). IR spectra (KBr) (^V^, cm^−1^), 3370 (NH), 3450 (OH), 3050 (Ar–H), 1150, 1305 (O=S=O); ^1^H NMR (400 MHz, DMSO-d6) δ: 2.32 (6H, s, 2CH_3_), 5.43 (1H, brs, OH), 6.75–7.75 (9H, m, Ar–H), 8.78 (1H, brs, CH), 9.75 (1H, brs, NH); ^13^C NMR (100 MHz, CDCl3): δ: 24.25 (2CH_3_), 110.15, 117.85, 120.44, 121.52, 122.58, 128.65, 132.21, 132.48, 138.37, 155.31, 161.13, 166.54, 168.62 (Ar-CH), 158.98 (CH=N); MS: m/z (%) 382 (M)^+^. Anal. Calcd for C_19_H_18_N_4_O_3_S: C, 59.67; H, 4.74; N, 14.65. Found C, 59.87; H, 4.55; N, 14.76.

#### 4-((2,4-dihydroxybenzylidene)amino)-*N*-(4,6-dimethylpyrimidin-2-yl)benzenesulfonamide (6e)

Orange powder, 94% yield, m.p. = 190–192 °C, Rf = 0.45 (7% methanol in CHCl_3_). IR spectra (KBr) (^V^, cm^−1^), 3370 (NH), 3450 (OH), 3050 (Ar–H), 1150, 1305 (O=S=O); ^1^H NMR (400 MHz, DMSO-d6) δ: 2.22 (6H, s, 2CH_3_), 5.67 (2H, brs, 2OH), 6.45–7.80 (9H, m, Ar–H), 8.85 (1H, brs, CH), 9.92 (1H, brs, NH); ^13^C NMR (100 MHz, CDCl3): δ: 23.35 (2CH_3_), 103.43, 108.73, 110.25, 113.23, 122.67, 128.68, 133.89, 138.29, 155.24, 162.31, 162.54, 166.47, 168.56 (Ar-CH), 160.14 (CH=N); MS: m/z (%) 399 (M+H)^+^. Anal. Calcd for C_19_H_18_N_4_O_4_S: C, 57.27; H, 4.55; N, 14.06. Found C, 57.45; H, 4.71; N, 14.25.

#### *N*-(4,6-dimethylpyrimidin-2-yl)-4-((3,4,5-trimethoxybenzylidene)amino)benzenesulfonamide (6f)

White powder, 93% yield, m.p. = 190–192 °C, Rf = 0.45 (7% methanol in CHCl_3_). IR spectra (KBr) (^V^, cm^−1^), 3370 (NH), 3050 (Ar–H), 1375 (CH_3_), 1150, 1305 (O=S=O); ^1^H NMR (400 MHz, DMSO-d6) δ: 1.98 (6H, s, 2CH_3_), 3.65 (9H, s, 3OCH_3_), 6.80–7.87 (7H, m, Ar–H), 8.76 (1H, brs, CH), 10.00 (1H, brs, NH); ^13^C NMR (100 MHz, CDCl3): δ: 22.86 (2CH_3_), 56.43 (2OCH_3_), 61.21 (OCH_3_), 104.03, 110.13, 113.23, 122.57, 128.46, 133.32, 138.43, 141.54, 153.23, 155.34, 166.61, 168.58 (Ar-CH), 163.22 (CH=N); MS: m/z (%) 458 (M+2H)^+^. Anal. Calcd for C_22_H_24_N_4_O_5_S: C, 57.88; H, 5.33; N, 12.27. Found C, 57.77; H, 5.56; N, 12.48.

#### 4-((4-chlorobenzylidene)amino)-N-(4,6-dimethylpyrimidin-2-yl)benzenesulfonamide (6g).

Yellow powder, 92% yield, m.p. = 212–214 °C, Rf = 0.45 (7% methanol in CHCl_3_). IR spectra (KBr) (^V^, cm^−1^), 3370 (NH), 3050 (Ar–H), 1150, 1305 (O=S=O); ^1^H NMR (400 MHz, DMSO-d6) δ: 1.88 (6H, s, 2CH_3_), 6.73–7.85 (7H, m, Ar–H), 8.56 (1H, brs, CH), 9.56 (1H, brs, NH); ^13^C NMR (100 MHz, CDCl3): δ: 24.16 (2CH_3_), 110.15, 122.60, 128.55, 128.98, 130.45, 134.76, 136.67, 138.54, 155.34, 166.71, 168.63 (Ar-CH), 160.00 (CH=N); MS: m/z (%) 400 (M)^+^. Anal. Calcd for C_19_H_17_ClN_4_O_2_S: C, 56.93; H, 4.27; N, 13.98. Found C, 57.08; H, 4.47; N, 14.11.

### Ethical approval

The ethical committee of Taif University, KSA, approved all procedures used in the current study.

## Computational study

### Preparation of the ligands and protein

From the RCSB protein data bank, the 3D structure of the protein was downloaded (ID: 4uro^23^). To observe the data from the proteins, the BLAST P tool was employed to analyse the FASTA sequence. Then, the CLUSTALW18 package was utilized for multiple amino acid alignments, as reported earlier^19,20,24–30^.

### Procedure for the docking experiment

The docking study was completed with the MOE 2015 package 28 [Molecular Operating Environment (MOE)]. The tested compounds were constructed, and their energy was minimized on the basis of PM3. DFT studies of the compounds based on B3LYP/6–311G were performed. Error correction for the structure of the catalytic site in DNA gyrase B (ID: 4uro) was performed by the supplemented hydrogen and partial charges using Amber12:EHT and then minimized by using the same force field with an RMSD value of 0.100. The catalytic site was identified and analysed by the Site Finder program. This method was based on alpha spheres as well as the energy model29. The catalytic zone was predicted by the MOE-Site finder30. The water and inhibitor molecules were eliminated, and the H atoms were supplemented to obtain the crystal structure. The charges were regulated using the MMFF94x force field. The alpha-site spheres were added on the basis of the Site Finder module. The final energies, generated poses, and scores were measured using the procedures mentioned above^31^.

## Antibacterial activity

### Bacterial isolates

*S. aureus*, *E. coli*, and *K. pneumoniae* were obtained from the Microbiology Unit of King Faisal Hospital after ethical approval from the Taif Directorate of Health Affairs, Taif, Saudi Arabia. The *S. aureus* and *E. coli* strains were originally isolated from urine samples. The *K. pneumoniae* strain was isolated from wounds. The strains were identified phenotypically, biochemically, serologically, and genotypically and saved in the LDF-9025U at − 80 °C. Each isolate was cultivated separately in tryptic soy broth (Difco) at 37 °C for 24 h. The cells were harvested by centrifugation at 5000*g* for 10 min. The cells were then washed twice with normal PBS and finally resuspended to obtain a final cell density of 1.8 × 10^8^ cfu/mL (OD600 0.2) in sterile saline solution (0.85% NaCl).

### Antimicrobial assay

The antibacterial activity of the synthesized compounds was assessed using the method of Duraipandiyan^32^ with slight modifications. Specifically, the bacterial counts of the *S. aureus*, *E. coli* and *K. pneumoniae* isolates were adjusted to 0.5 McFarland turbidity standards using sterile saline solution. The bacterial cultures were poured onto sterile Petri dishes containing Mueller–Hinton agar (Difco). The freshly cultured bacterial isolates were uniformly spread using sterile cotton swabs. A total volume of 100 µL of each stock solution of the synthesized chemicals was dissolved in DMSO and loaded in 6 mm wells. Later, the plates were incubated at 37 °C for 24 h. The results are represented as the size of the inhibition zone around the wells. The negative control wells contained 100 µL of DMSO.

### Minimum inhibitory concentrations

The synthesized sulfadimidine derivatives exhibited antibacterial activity at a concentration of 100 mg in DMSO (w/v). Therefore, this concentration was selected to determine their minimum inhibitory concentration (MIC) using the agar well diffusion method33. The substrates were diluted twice (tenfold dilutions). Approximately 1 mL of each freshly prepared bacterial inoculum was pipetted into sterile Petri dishes, followed by the addition of molten Muller–Hinton agar. Later, five wells were made on each plate, and 100 µL of each diluted substance was transferred to the respective wells in a clockwise manner. The plates were kept in the refrigerator for 20 min and then incubated overnight at 37 °C. The MIC was considered the lowest concentration at which the growth of the respective bacteria was inhibited. DMSO was used as the negative control.

## Conclusions

This study focused on the synthesis of new nucleoside and Schiff base derivatives and their activities against bacterial strains. Our study concluded that the arabinose nucleoside 3a and acetylated mannose nucleoside 4d derived from sulfadimidine were the most active against *S. aureus* while 4d and the acetylated glucose nucleoside 4c were the most active against *K. pneumoniae*. Finally, 4d, 4c and the acetylated galactose nucleoside 4b were the most active against *E. coli.* The MIC values against *S. aureus* ranged from 50 mg to 3.125 mg, while those against *E. coli* and *K. pneumoniae* ranged from 50 mg to 1562 mg. In vitro, the antibacterial effects were promising. Further research is required to study the in vivo antibacterial effects of these compounds and determine therapeutic doses.

## Supplementary Information

Supplementary Information 1.

Supplementary Information 2.

## Data Availability

Data are available upon request.

## Abbreviations

AcOH: Acetic acid
Ac_2_O: Acetic anhydride
ATCC 25923: *Staphylococcus aureus* Subsp. *aureus* Rosenbach
ATCC 6633: *Bacillus subtilis* Subsp. *spizizenii* Nakamura et al.
ATCC 14028: *Salmonella enterica* Subsp. *enterica* (ex Kauffmann and Edwards) Le Minor and Popoff serovar Typhimurium
ATCC 12022: *Shigella**flexneri* Castellani and Chalmers
EtOH: Ethanol
POA 286: *P. aeruginosa*
MIC: Minimum inhibitory concentration
VERO: VERO cell
RMSD: Root mean square deviation
PBM: Pharmacy Benefits Management
CEM: Construction Engineering and Management
MOE: Molecular Operating Environment
MD: Molecular dynamics
NAMD: National Association of Medicaid Directors
EHT: Extended Hückel Theory
PLIFs: Protein–ligand interaction fingerprints
CDCl_3_: Deuterated chloroform
TMS: Trimethyl silane
TLC: Thin layer chromatography
DFT: Discrete Fourier transform
LDF-9025U: Deep freezer LDF 9025U
OD600 0.2: Orion (Knight Electronics, Inc.)
DMSO: Dimethyl sulfoxide
