# Supplementary material for: Antibacterial and molecular docking studies of newly synthesized nucleosides and Schiff bases derived from sulfadimidines
Source: Sci Rep. 2021 Sep 9;11:17953. doi: 10.1038/s41598-021-97297-1 (PMC8429437; doi:10.1038/s41598-021-97297-1)
Supplement: Supplementary file 1 — Supplementary Information 1. [file 41598_2021_97297_MOESM1_ESM.pdf]

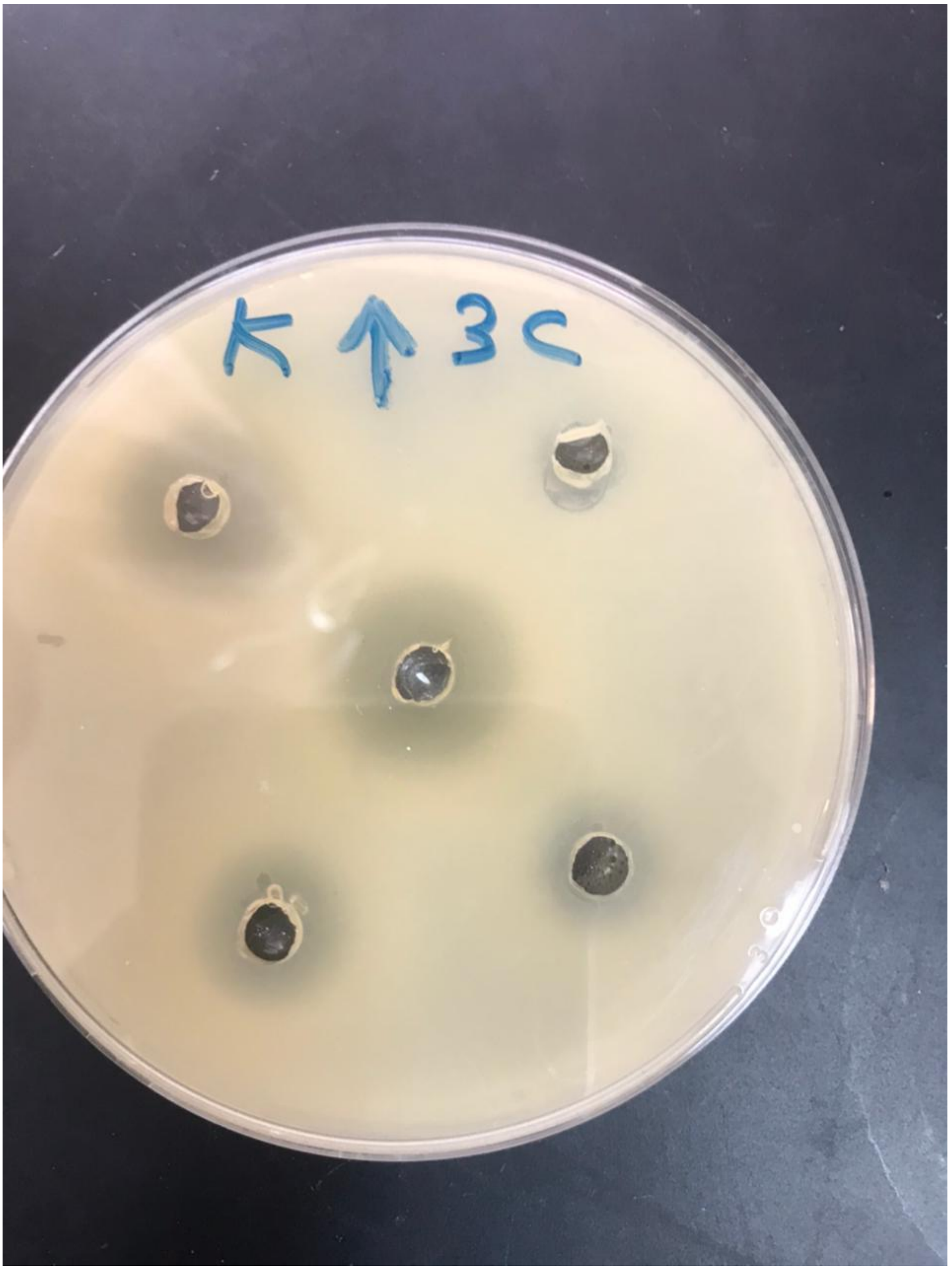

Fig 7. Antibacterial of 3c against *K. pneumoniae*.

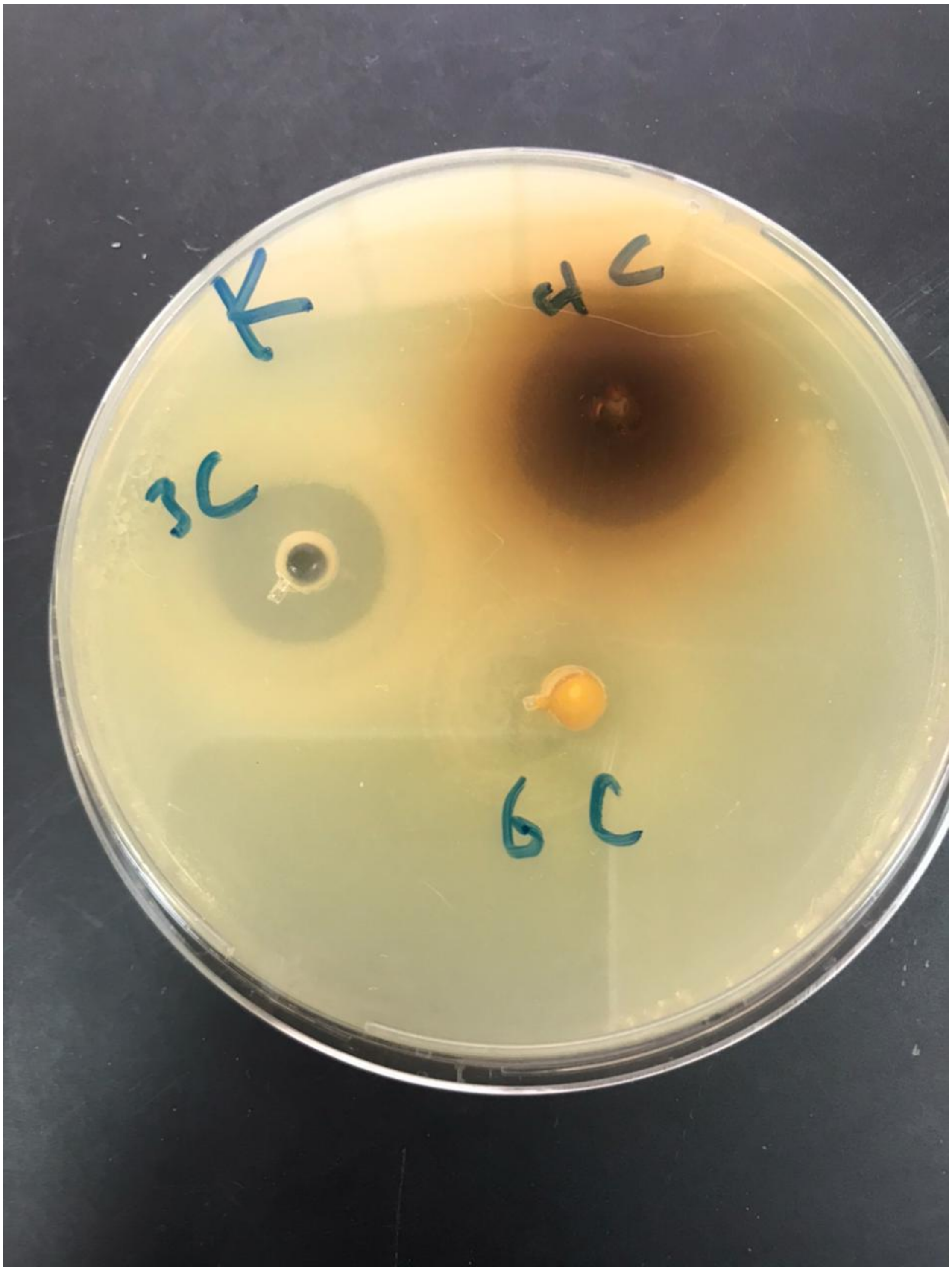

Fig 8. Antibacterial of 3c, 4c and 6c against *K. pneumoniae*.

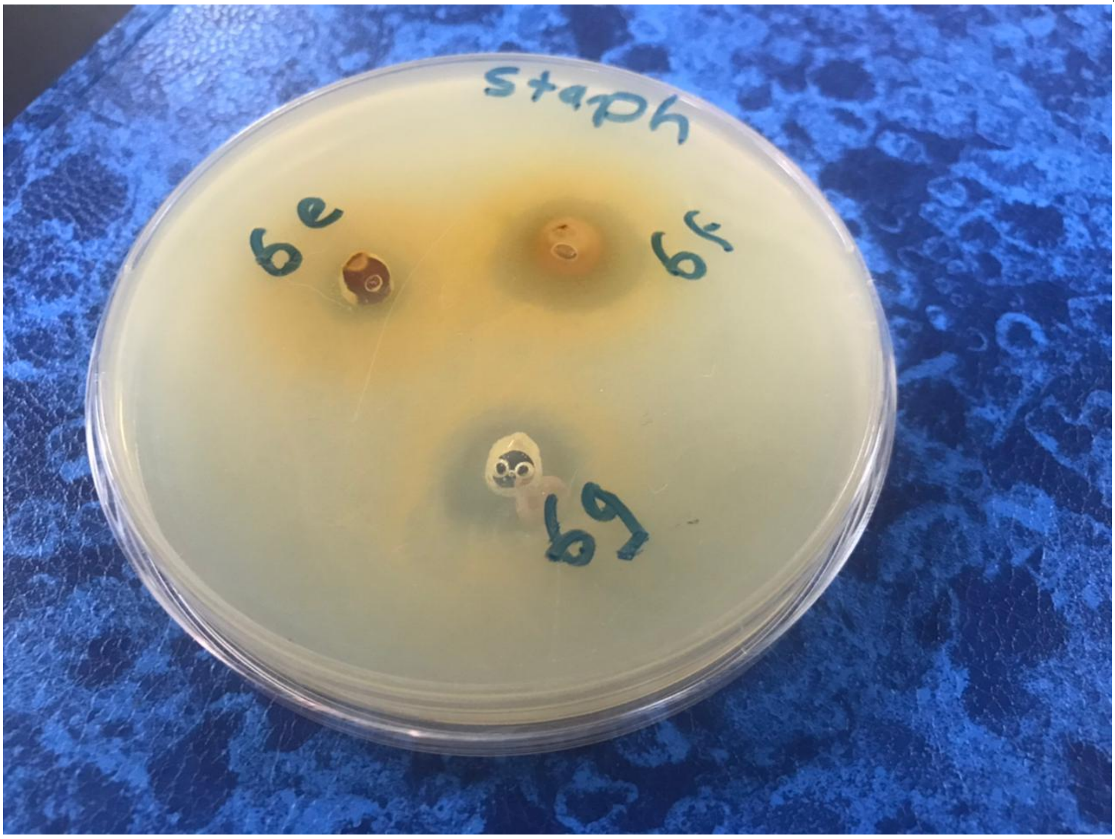

Fig 9. Antibacterial of 6e, 6f and 6g against *S. aureus*

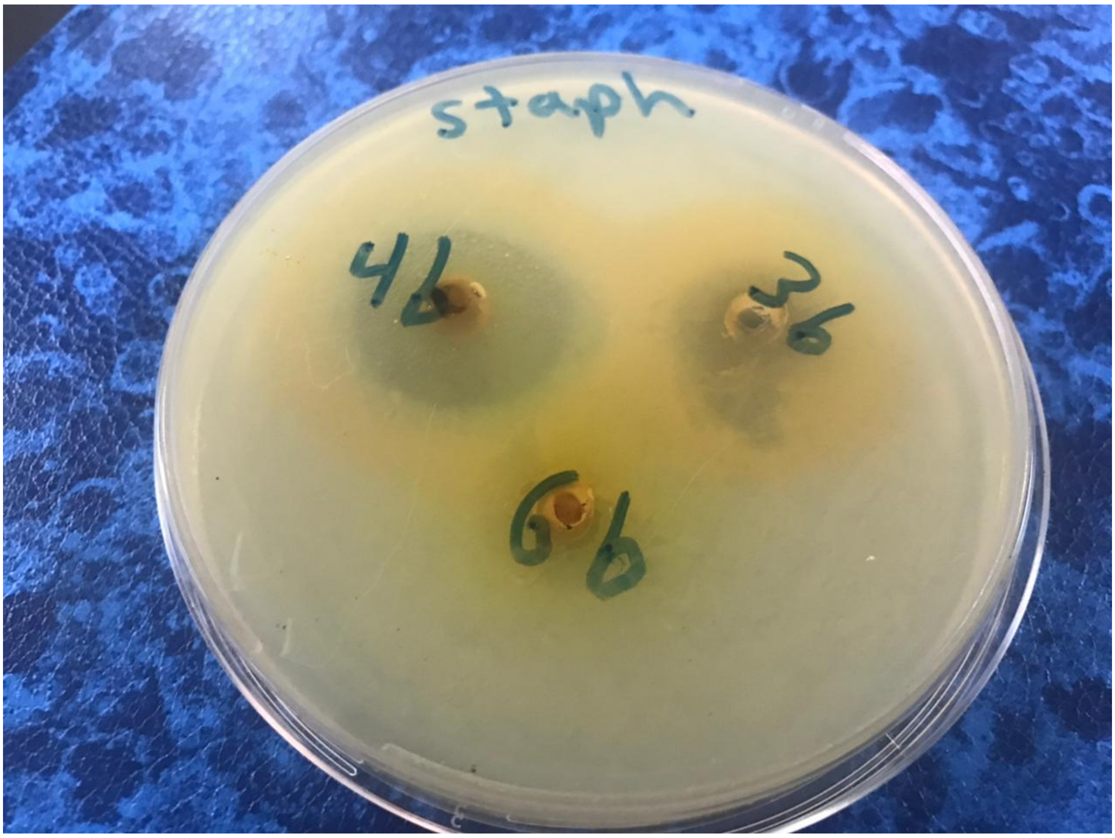

Fig 10. Antibacterial of 6e, 6f and 6g against *S. aureus*

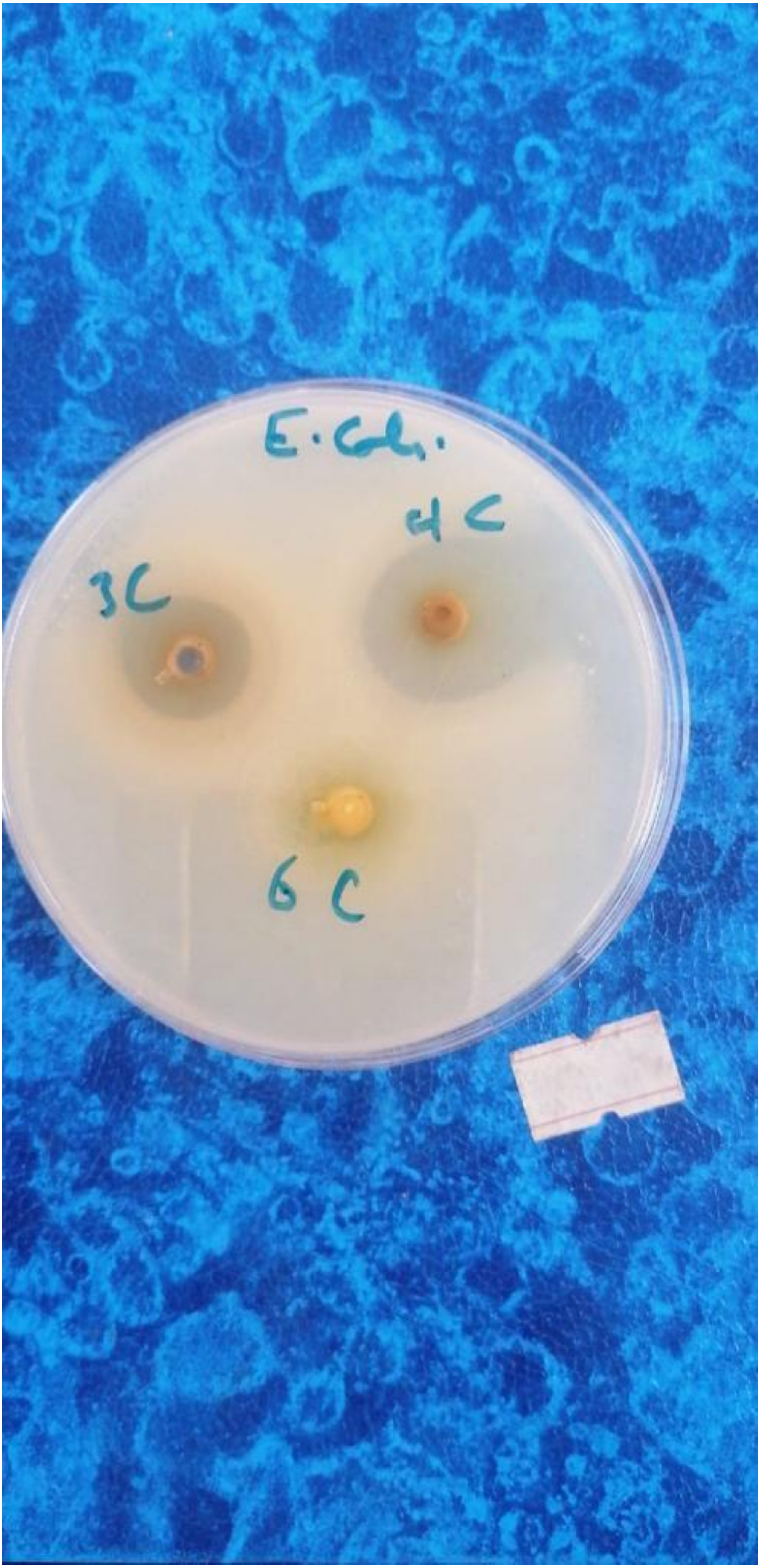
E. Coli.
4 C
3C
6 C

Fig 11. Antibacterial of 3c, 4c and 6c against *E. coli*

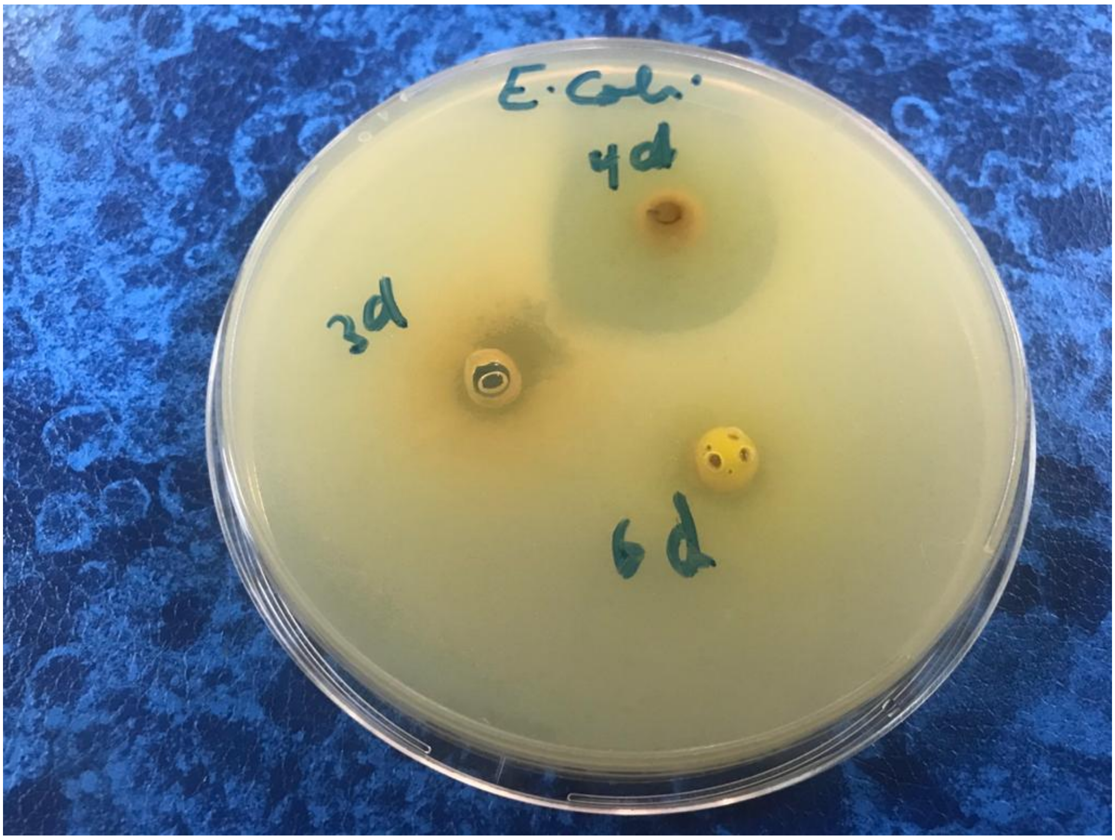

Fig 12. Antibacterial of 3d, 4d and 6d against *E. coli*

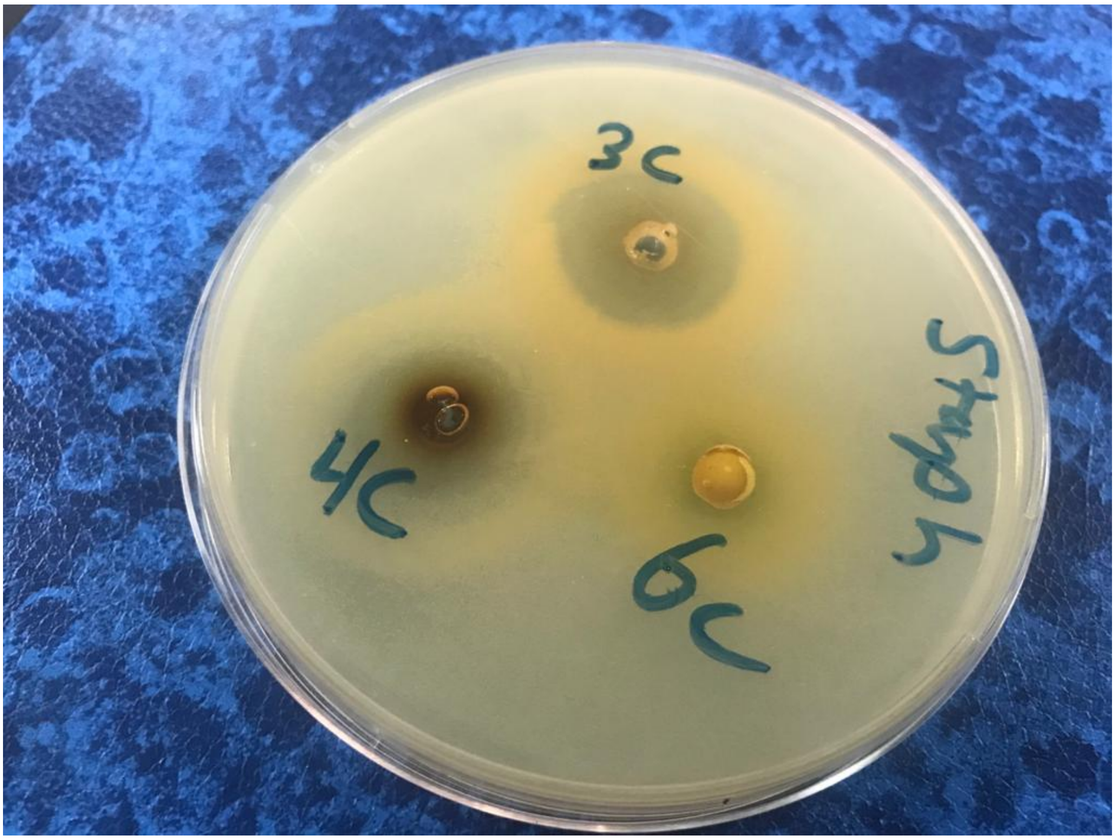

Fig 13. Antibacterial of 6e, 6f and 6g against *S. aureus*

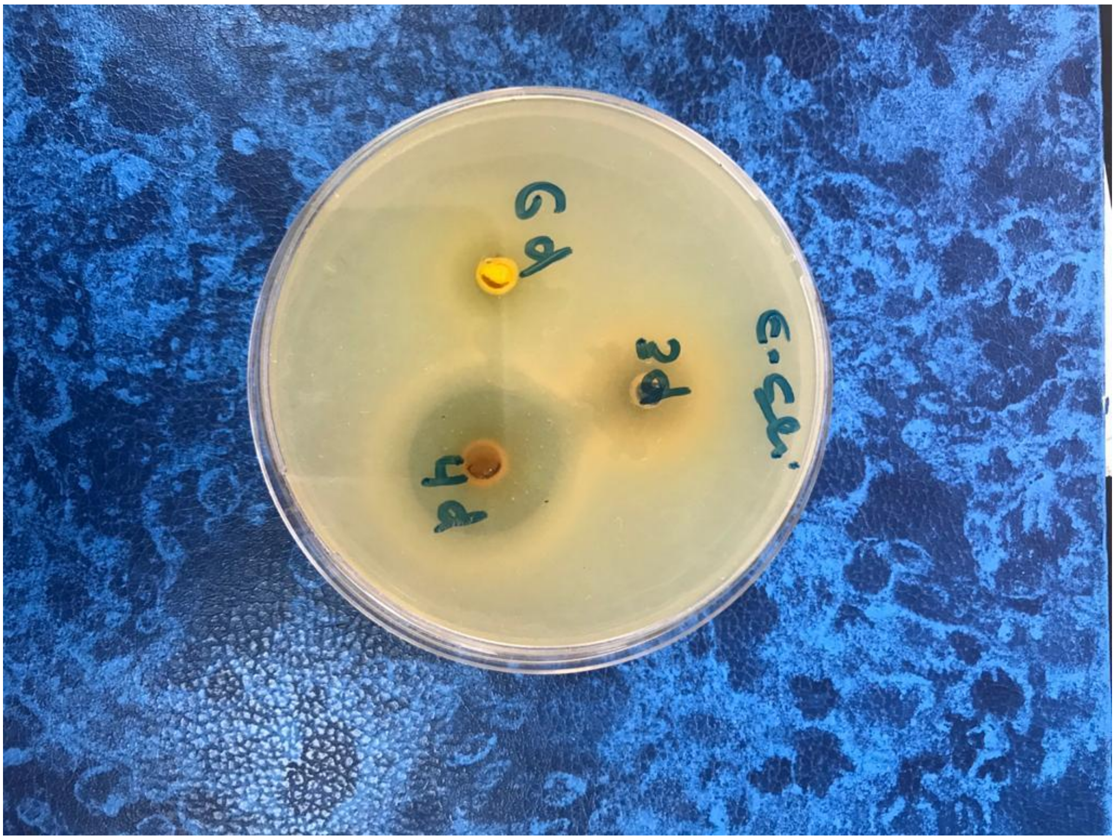

Fig 14. Antibacterial of 3d, 4d and 6d against *E. coli*

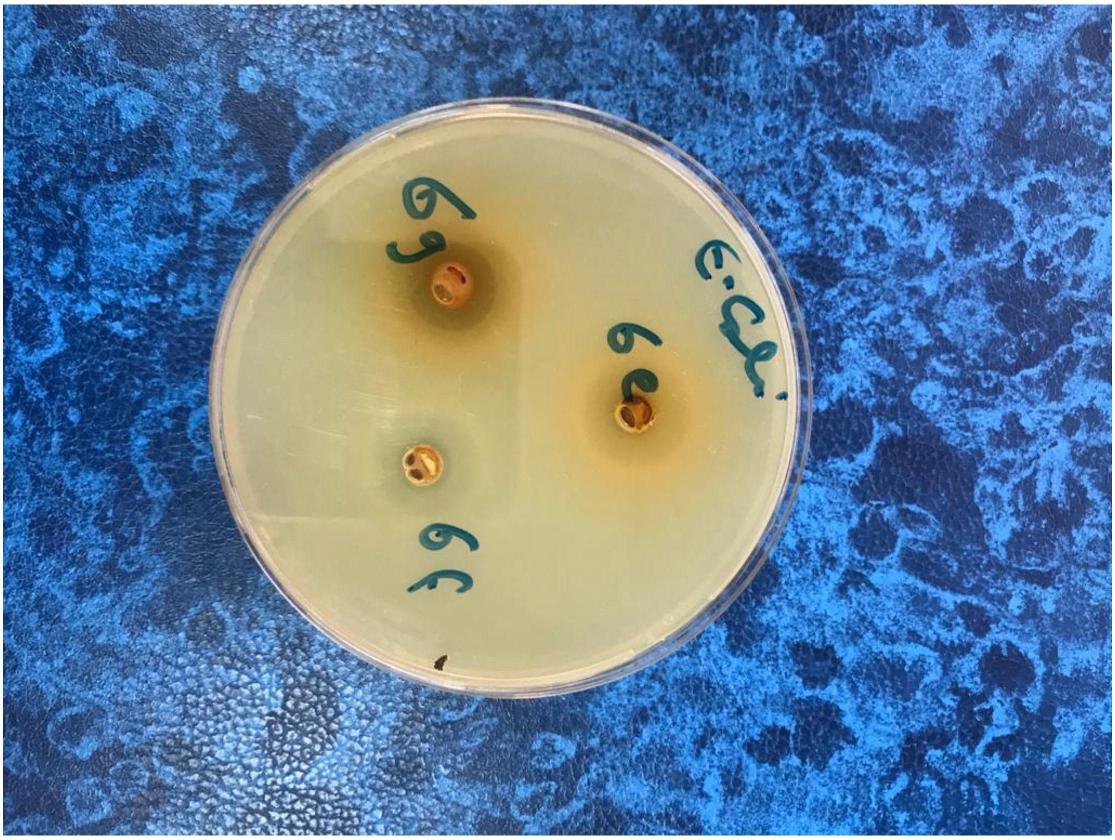

Fig 15. Antibacterial of 6e, 6f and 6g against *E. coli*

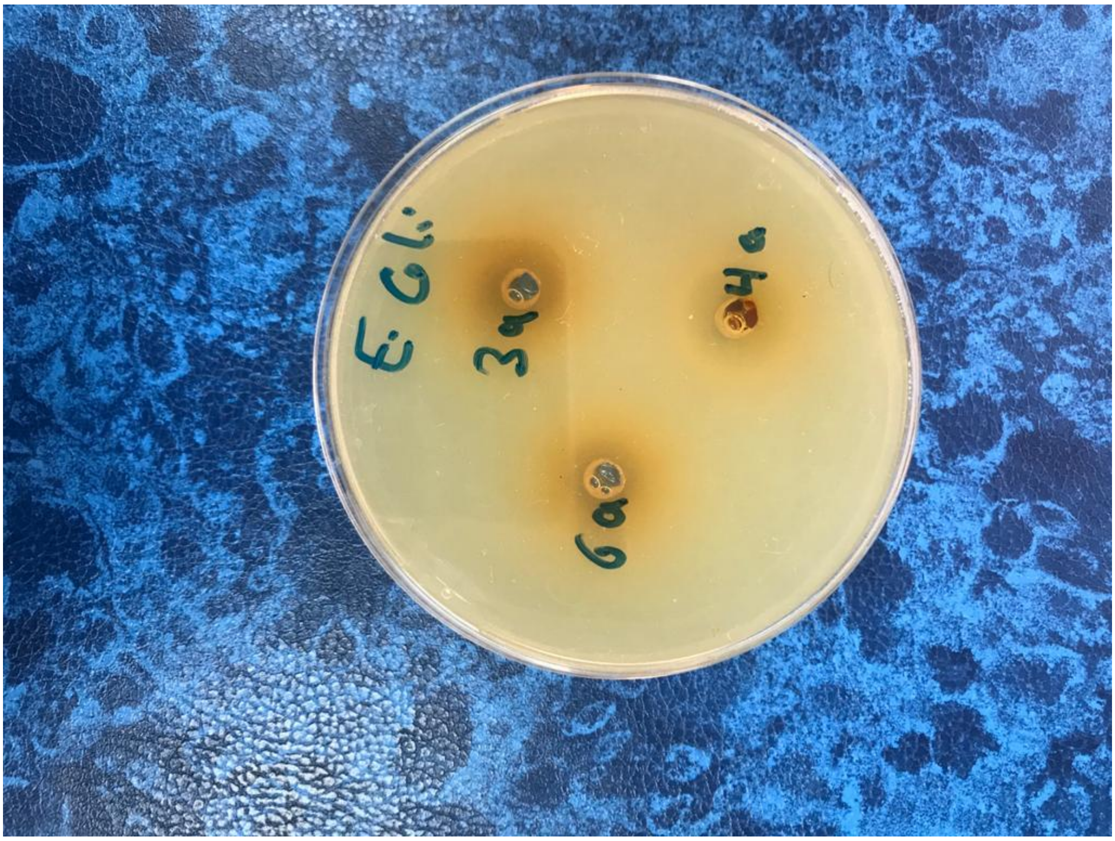

Fig 16. Antibacterial of 3a, 4a and 6a against *E. coli*

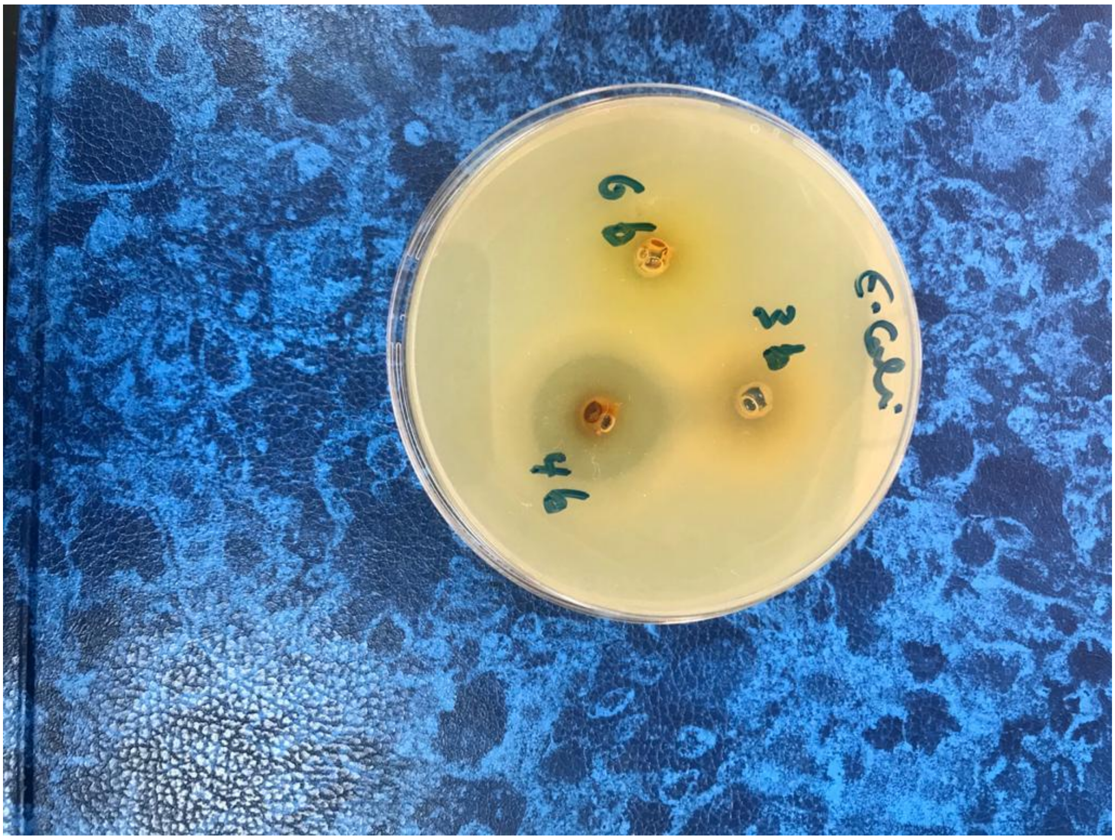

Fig 17. Antibacterial of 3d, 4d and 6d against *E. coli*

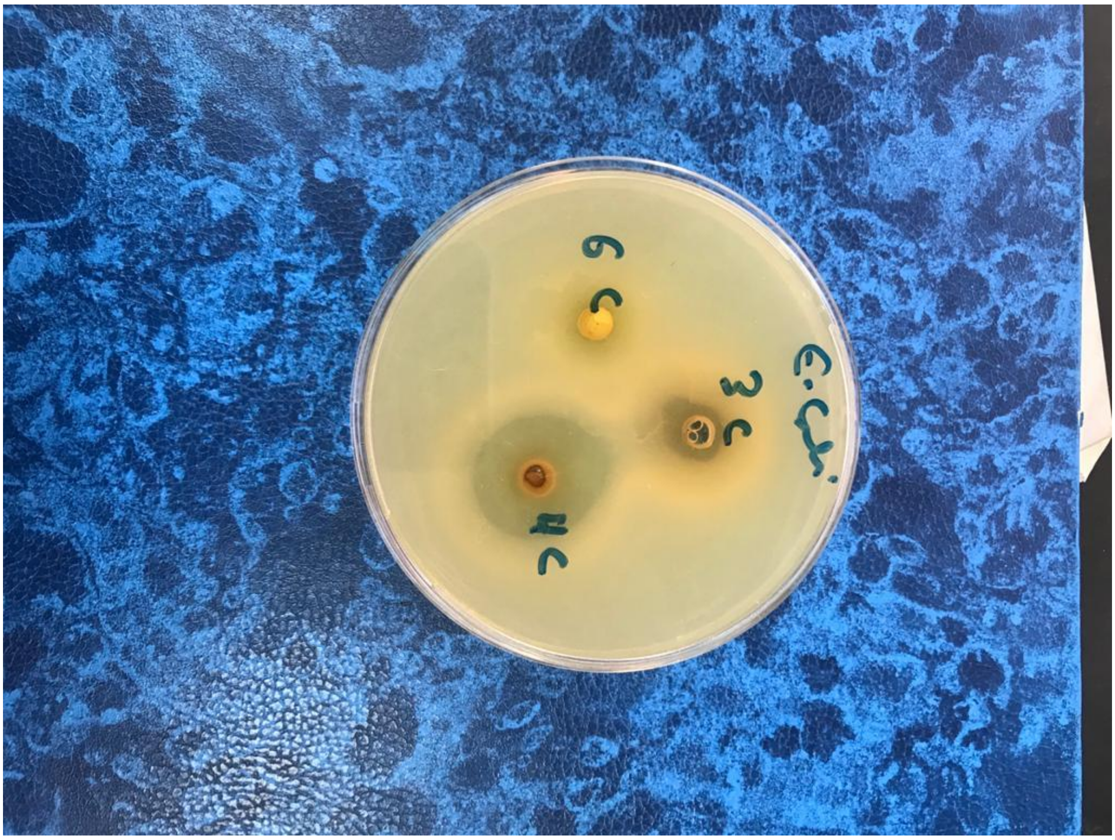

Fig 18. Antibacterial of 3c, 4c and 6c against *E. coli*

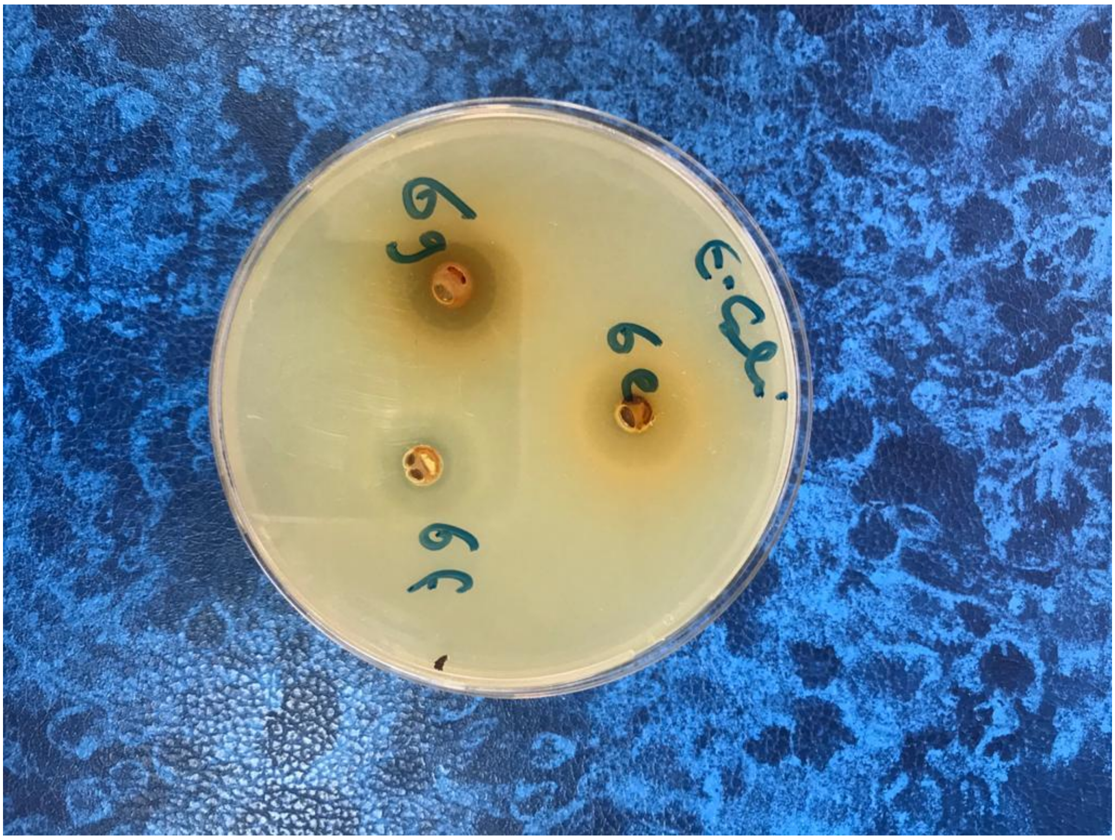

Fig 19. Antibacterial of 6e, 6f and 6g against *E. coli*

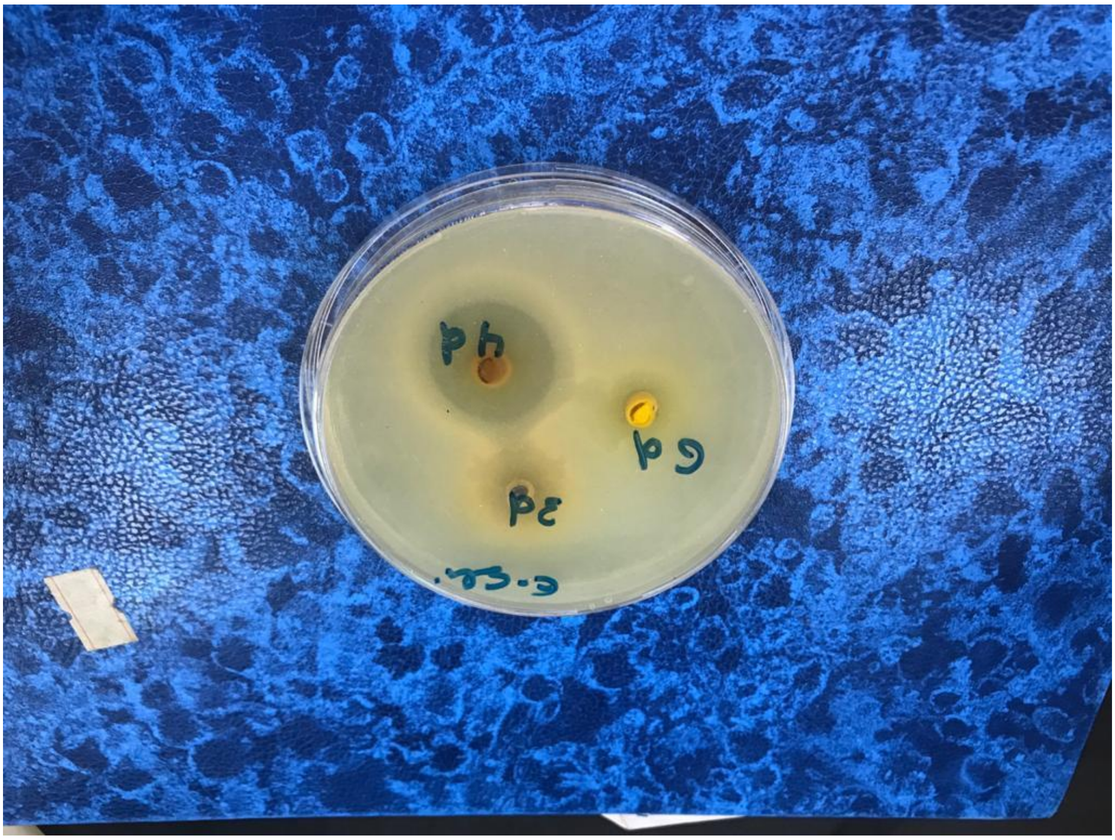

Fig 20. Antibacterial of 3d, 4d and 6d against *E. coli*

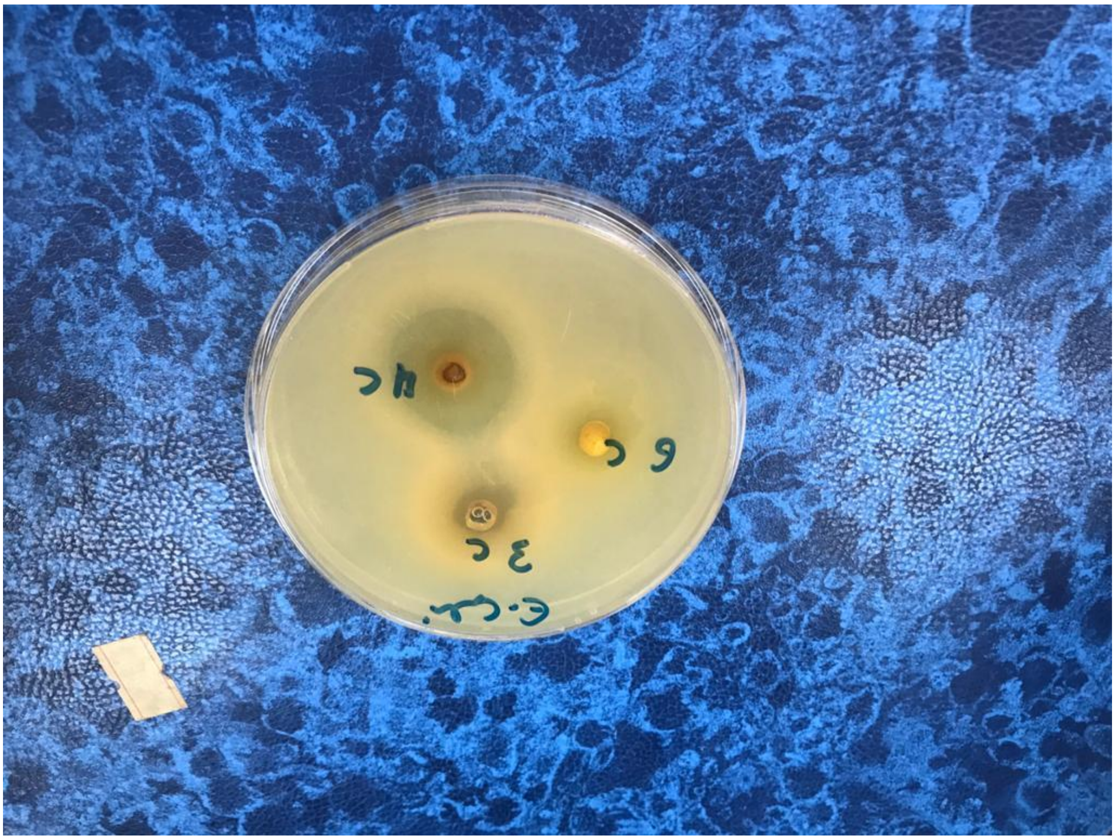

Fig 21. Antibacterial of 3c, 4c and 6c against *E. coli*

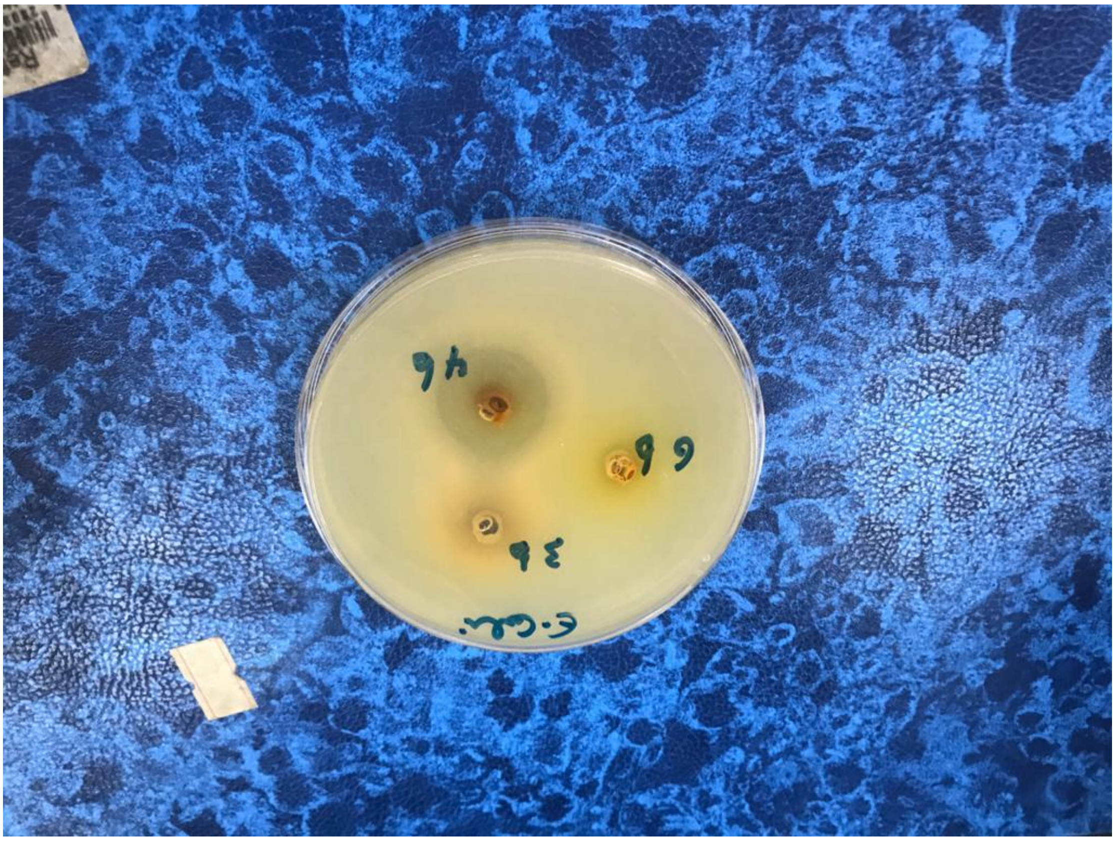

Fig 22. Antibacterial of 3b, 4b and 6b against *E. coli*

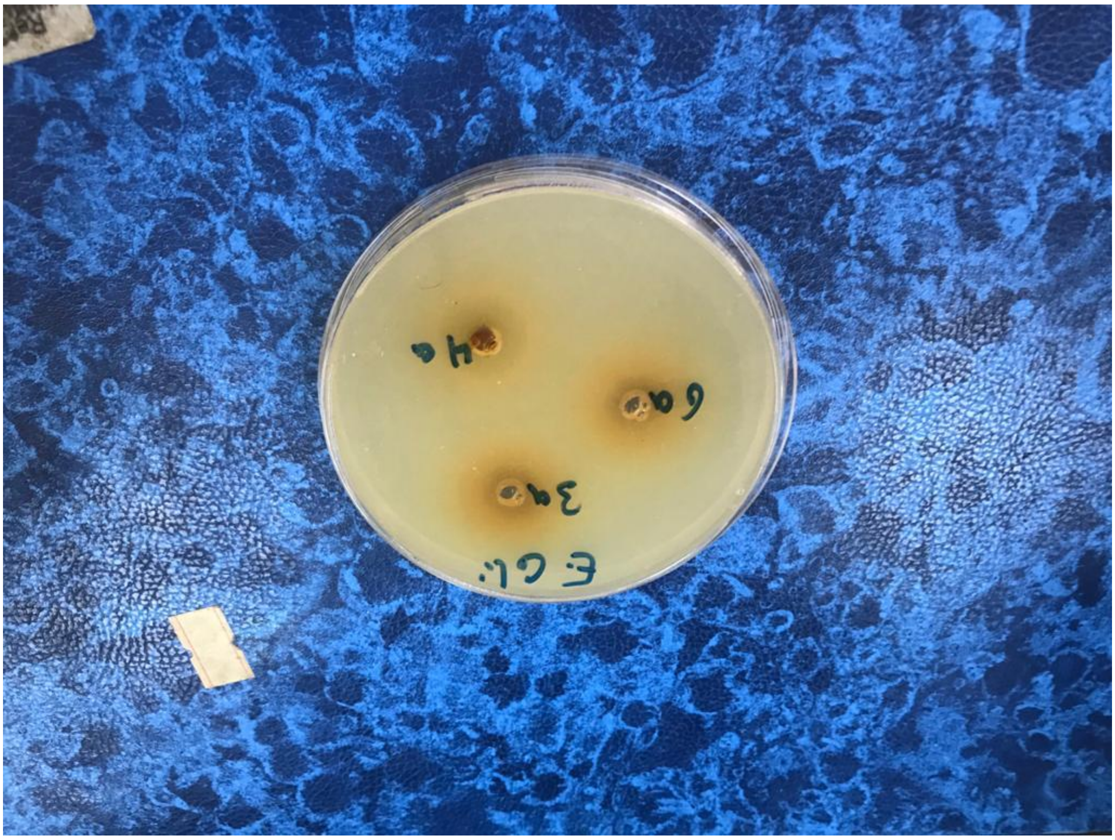

Fig 23. Antibacterial of 3a, 4a and 6a against *E. coli*

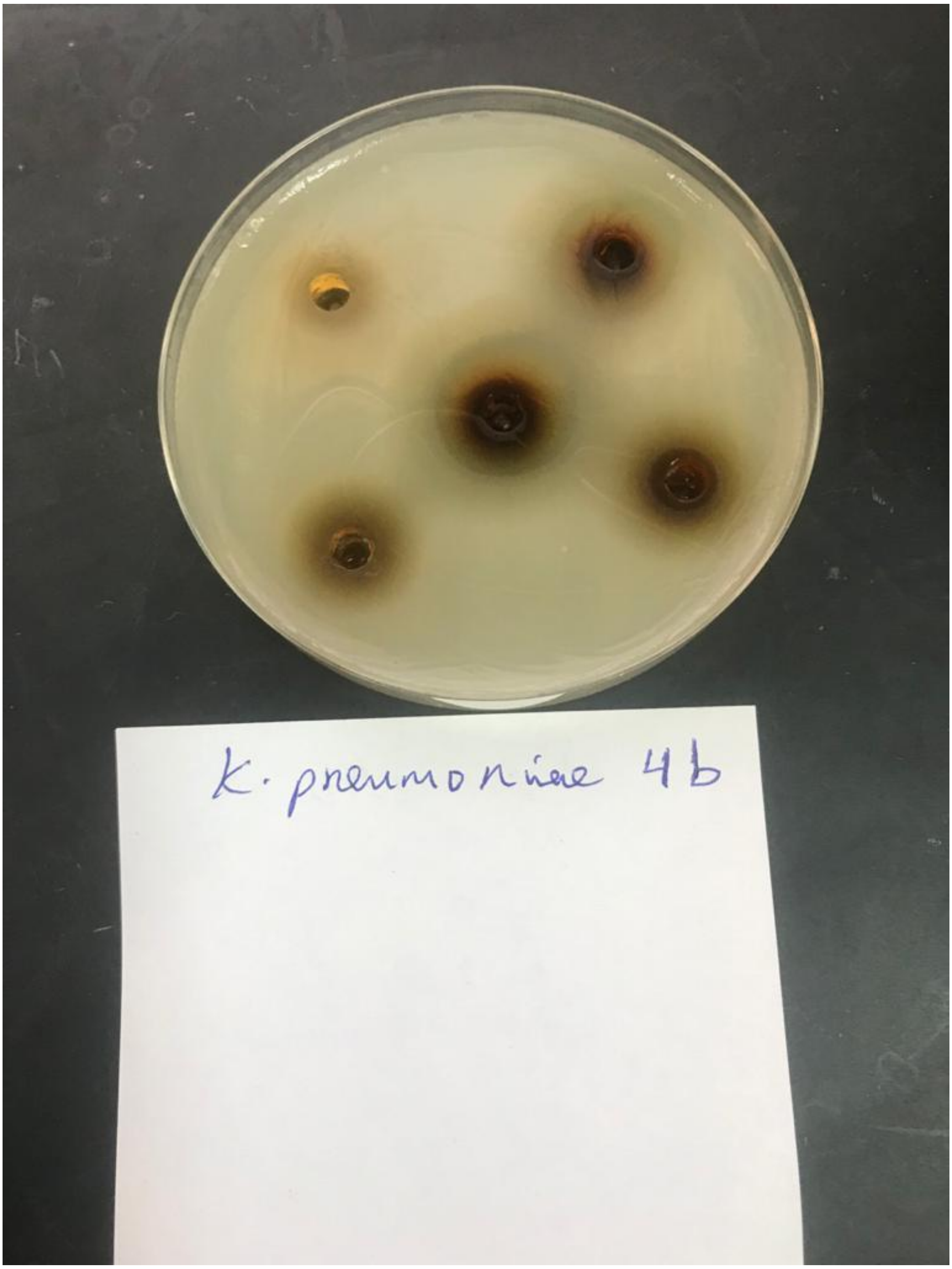

Fig 24. Antibacterial of 4b against *K. pneumoniae*.

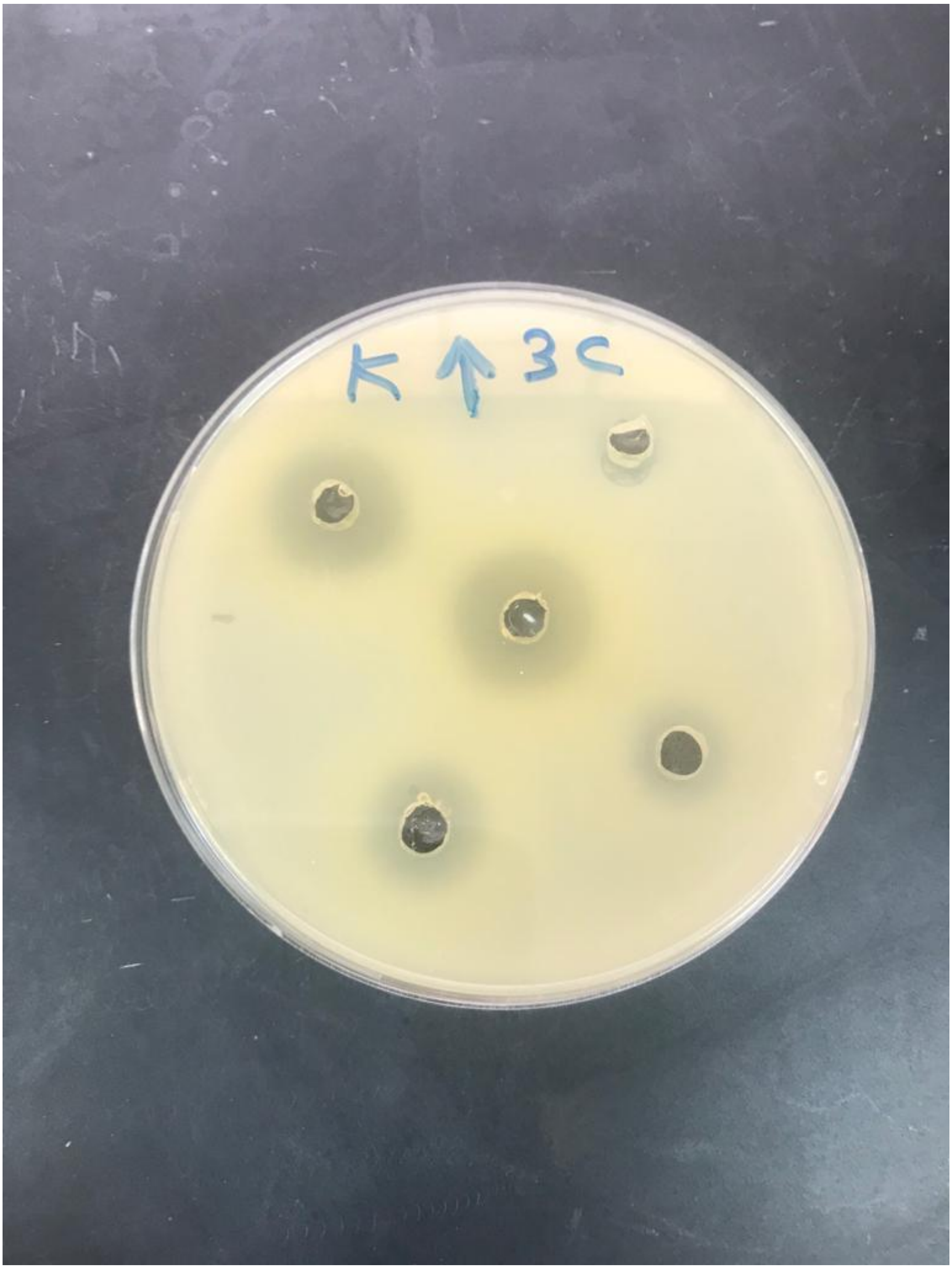

Fig 25. Antibacterial of 3c against *K. pneumoniae*.

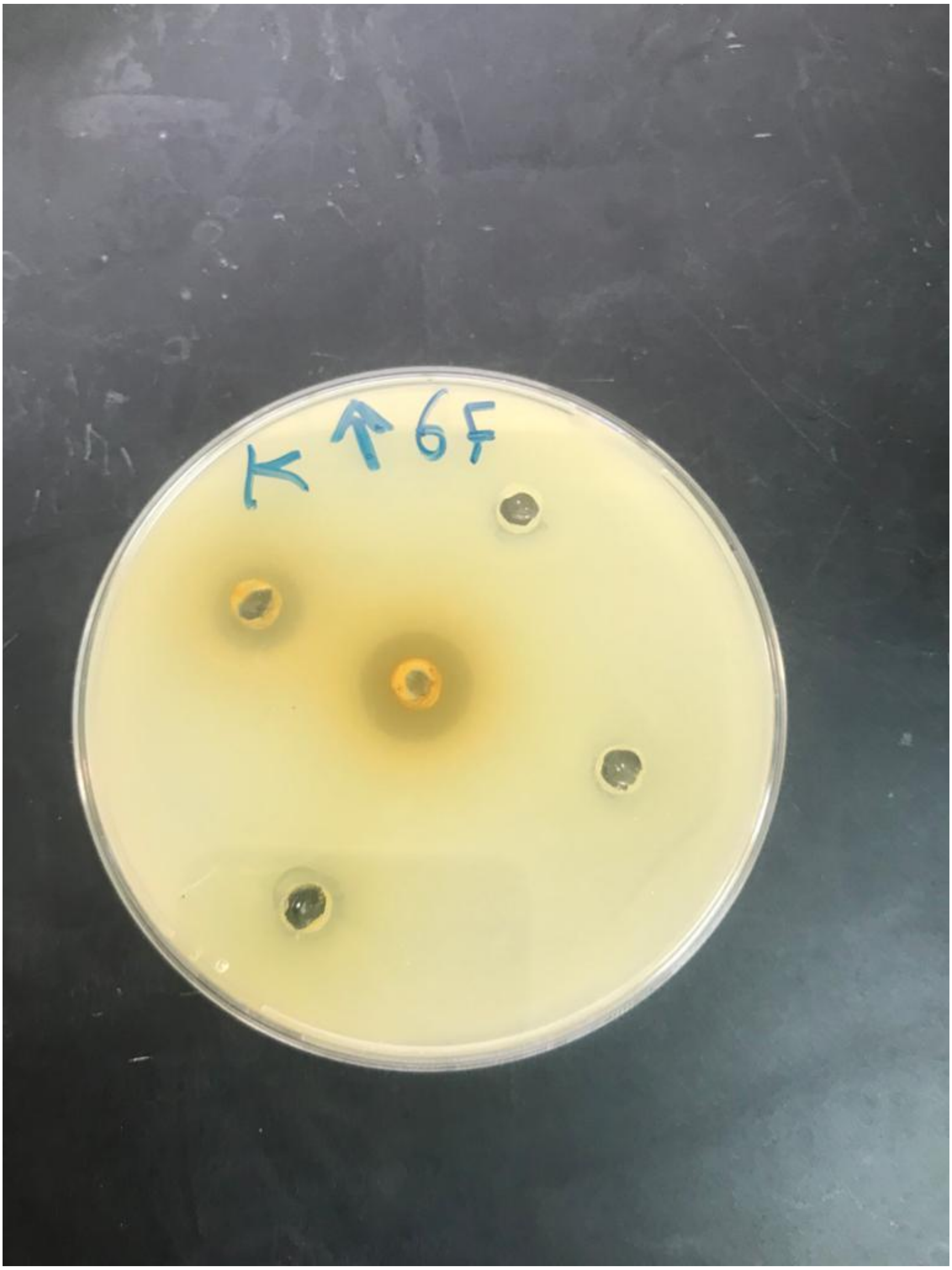

Fig 26. Antibacterial of 6f against *K. pneumoniae*.

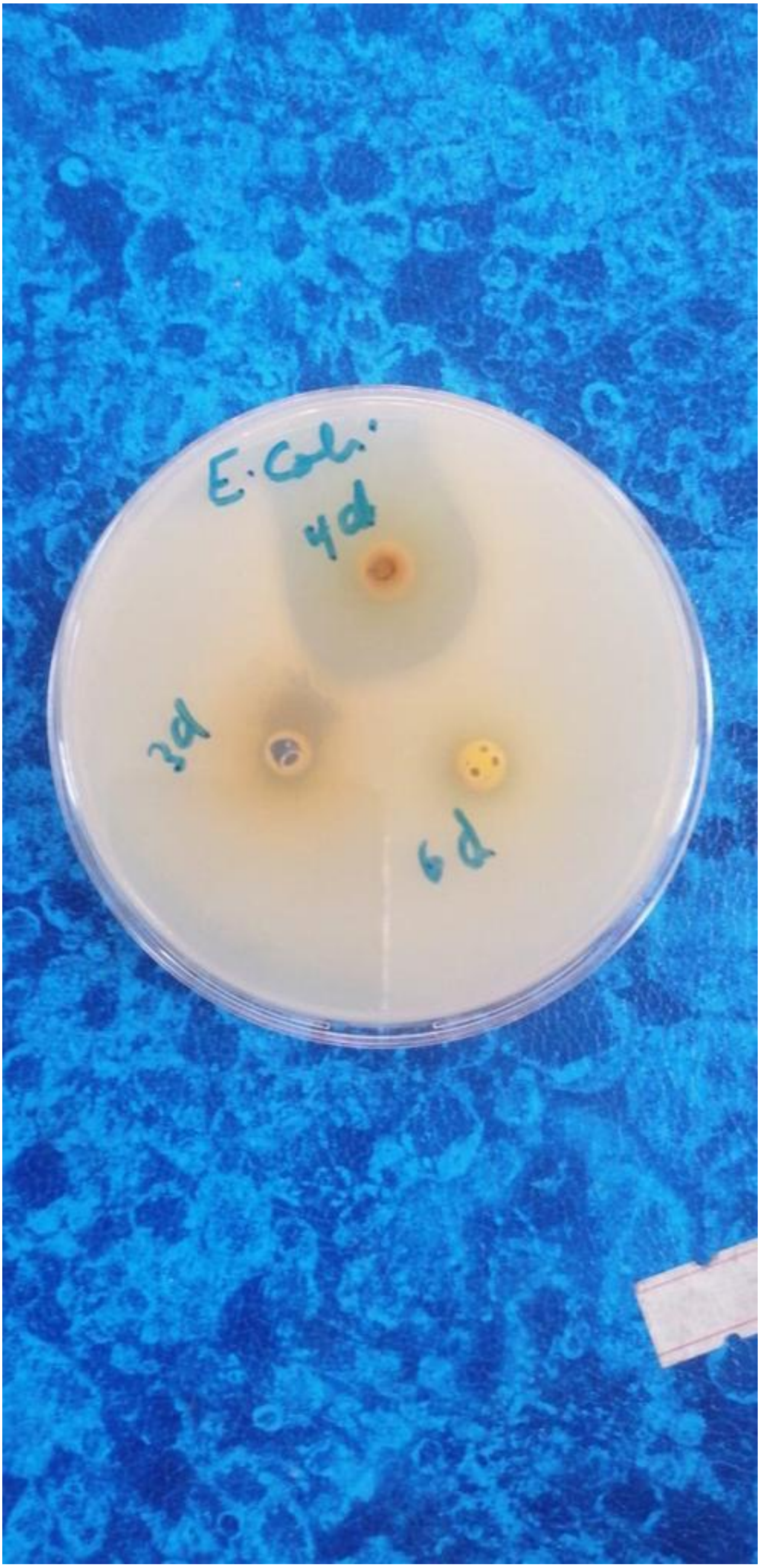
E.Coli
4d
3d
6d

Fig 27. Antibacterial of 3d, 4d and 6d against *E. coli*

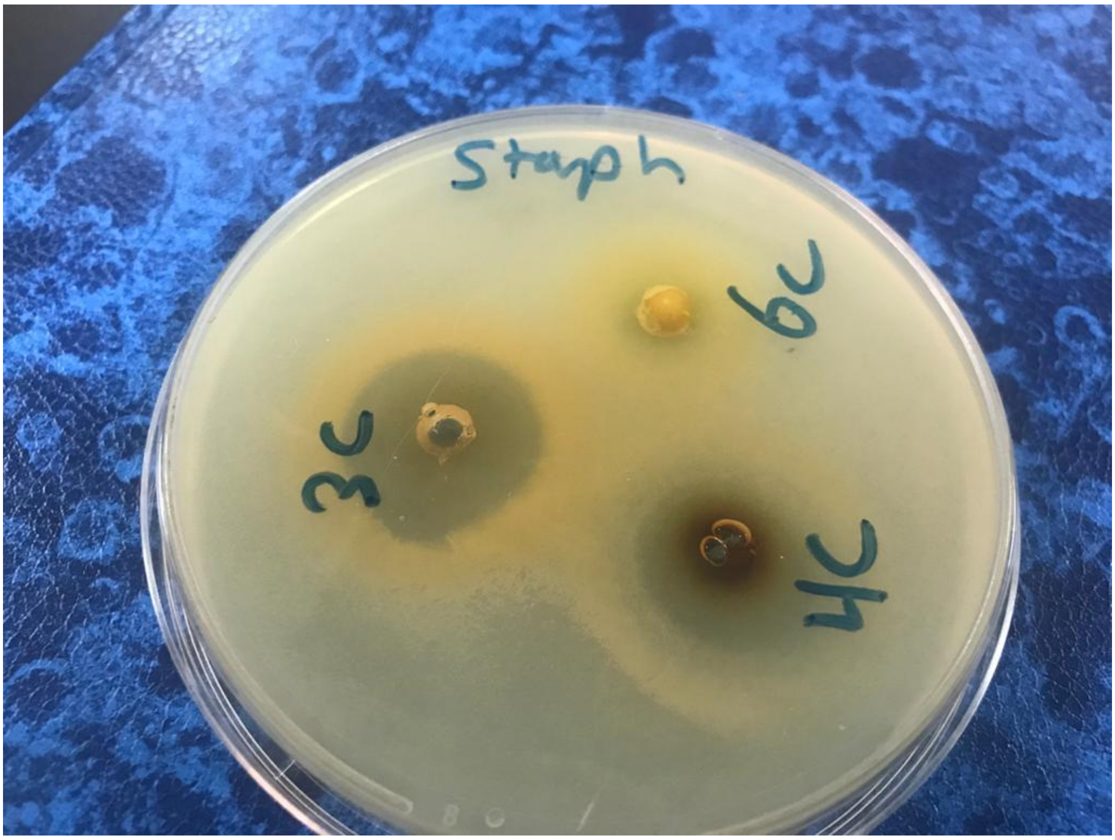

Fig 28. Antibacterial of 3c, 4c and 6c against *S. aureus*

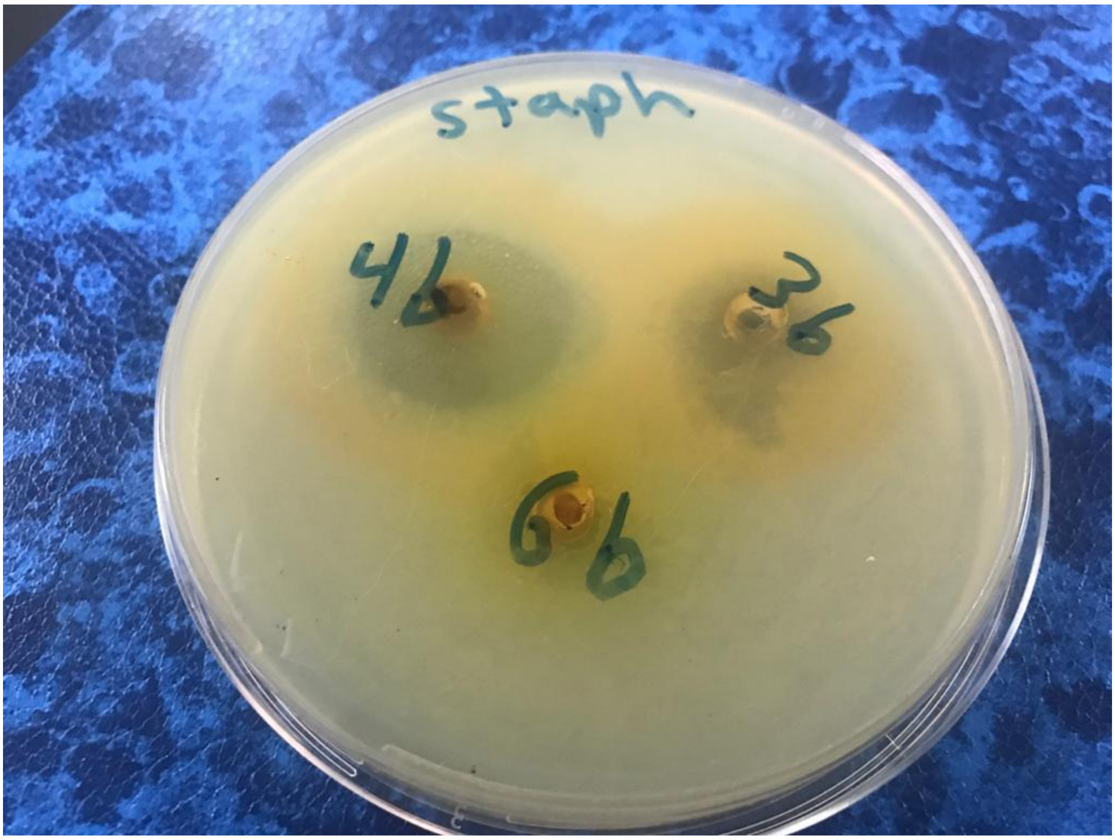

Fig 29. Antibacterial of 3b, 4b and 6b against *S. aureus*

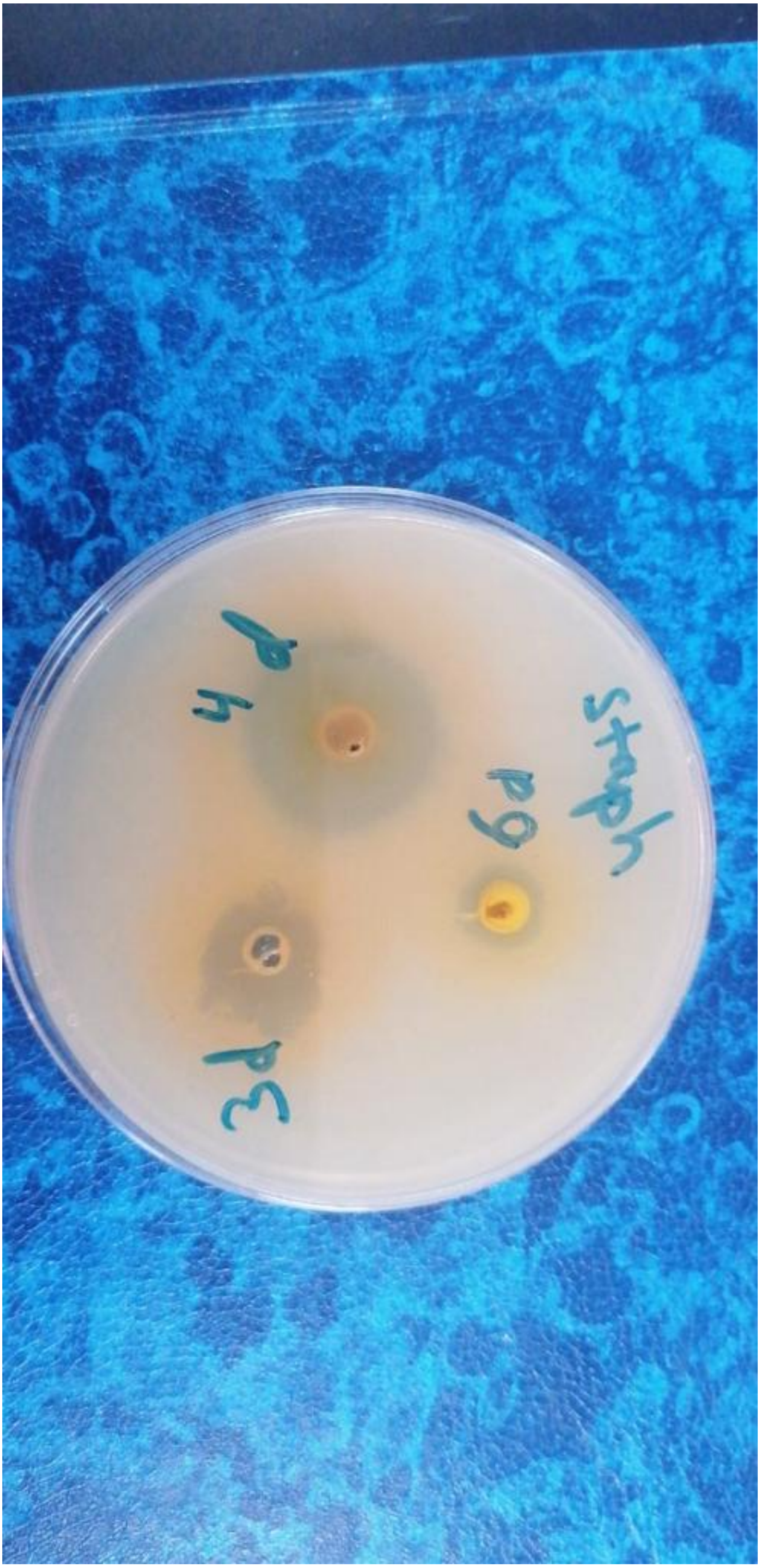

Fig 30. Antibacterial of 3d, 4d and 6a against *S. aureus*

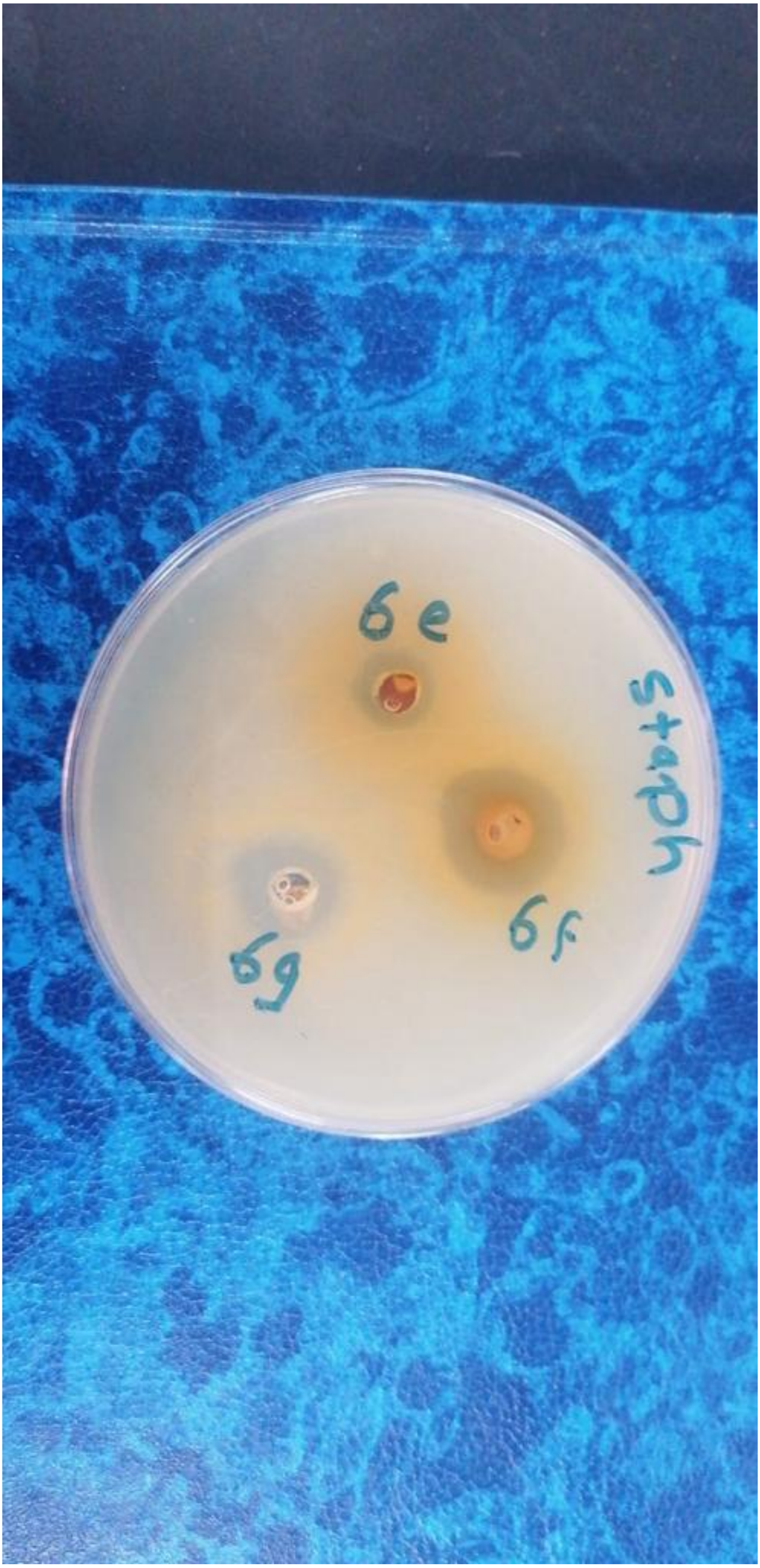
6e
6f
6g
Staph

Fig 31. Antibacterial of 6e, 6f and 6g against *S. aureus*

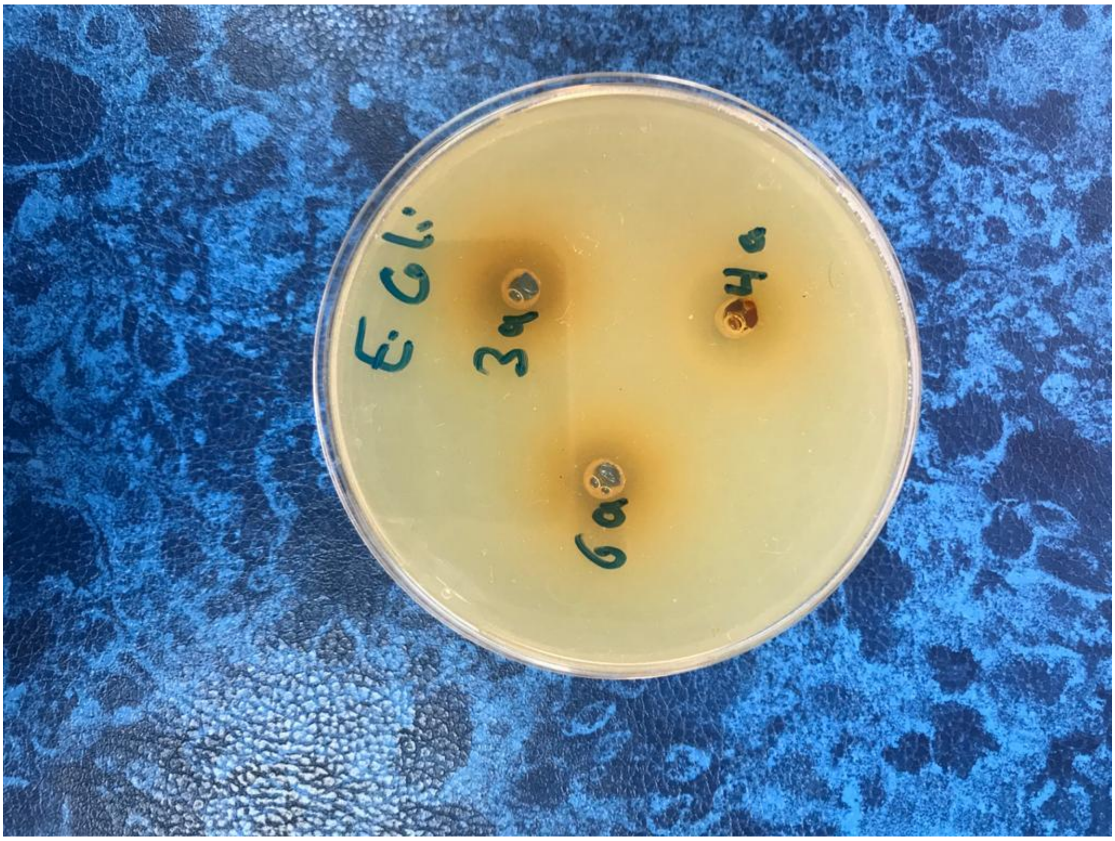

Fig 32. Antibacterial of 3a, 4a and 6a against *E. coli*

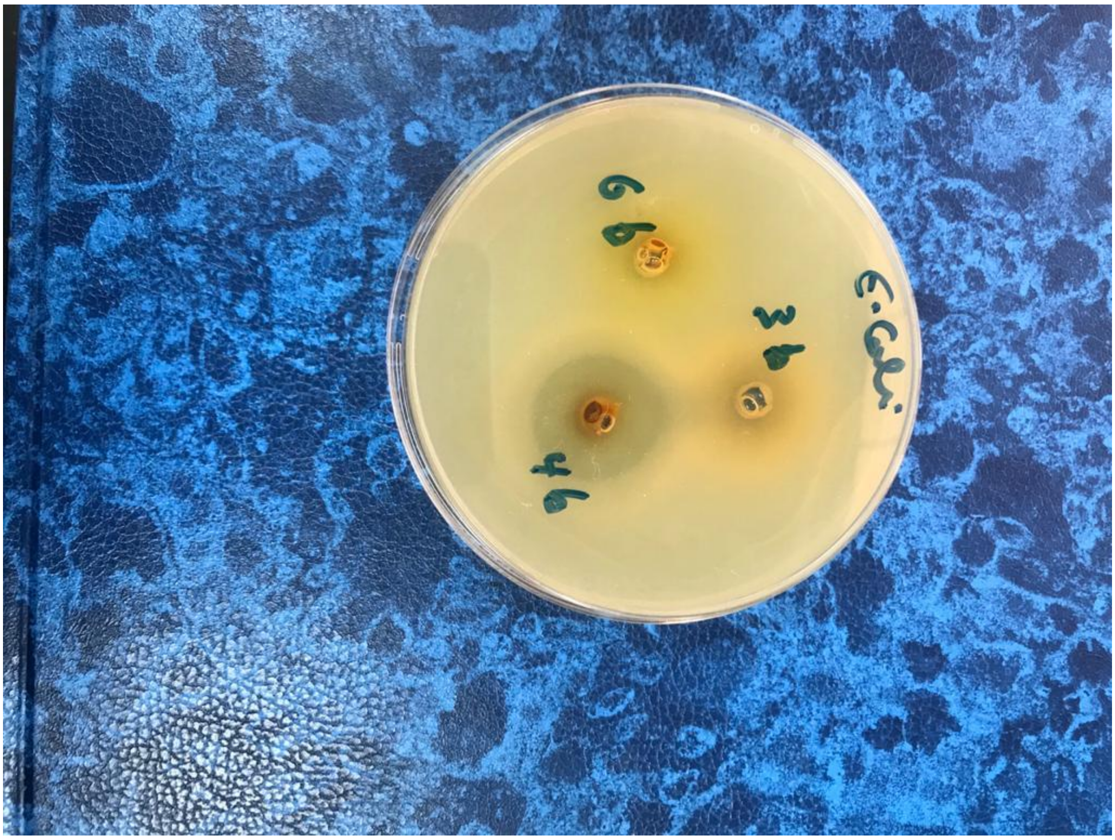

Fig 33. Antibacterial of 3b, 4b and 6b against *E. coli*

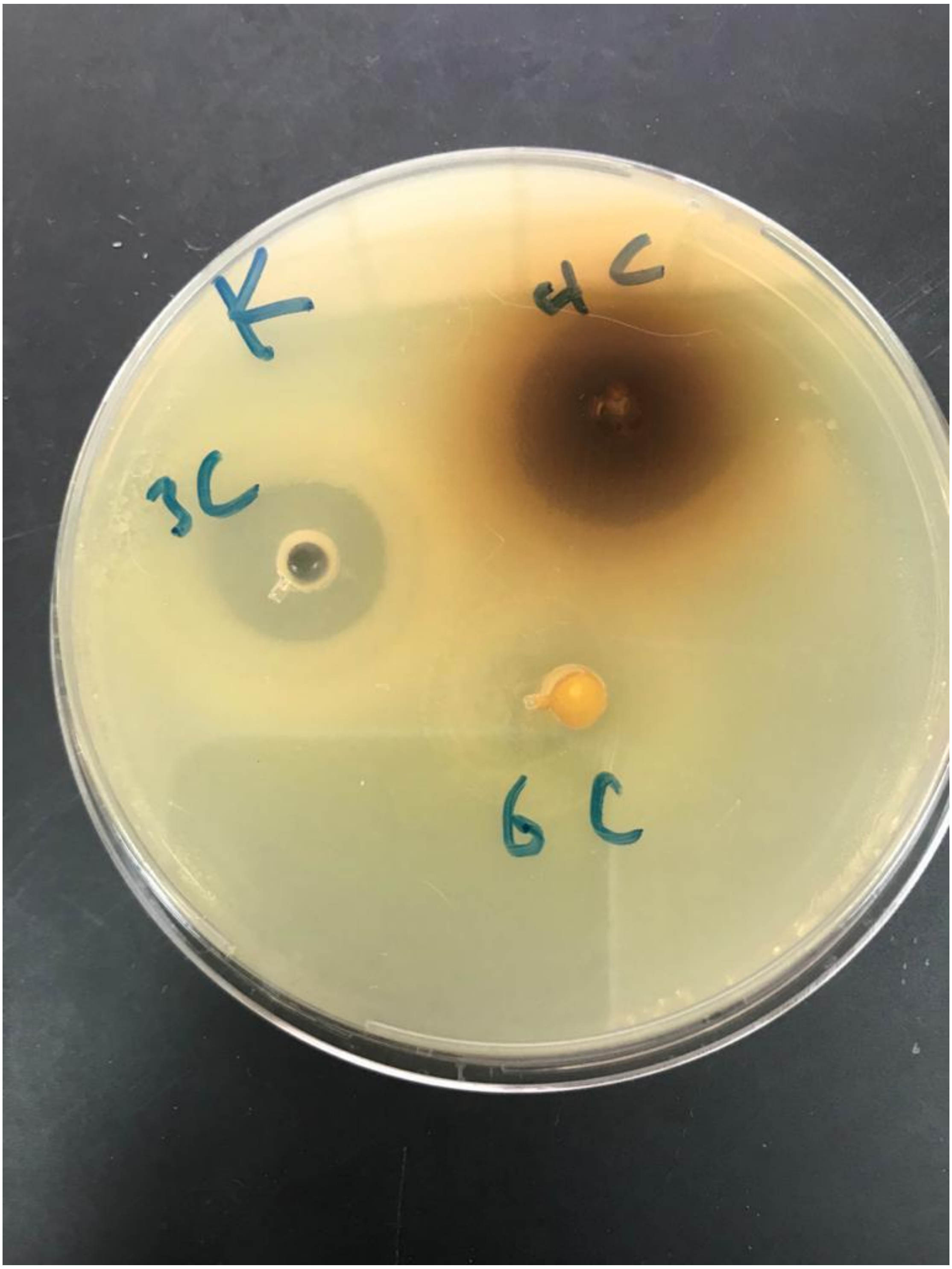

Fig 34. Antibacterial of 3c, 4c and 6c against *K. pneumoniae*.

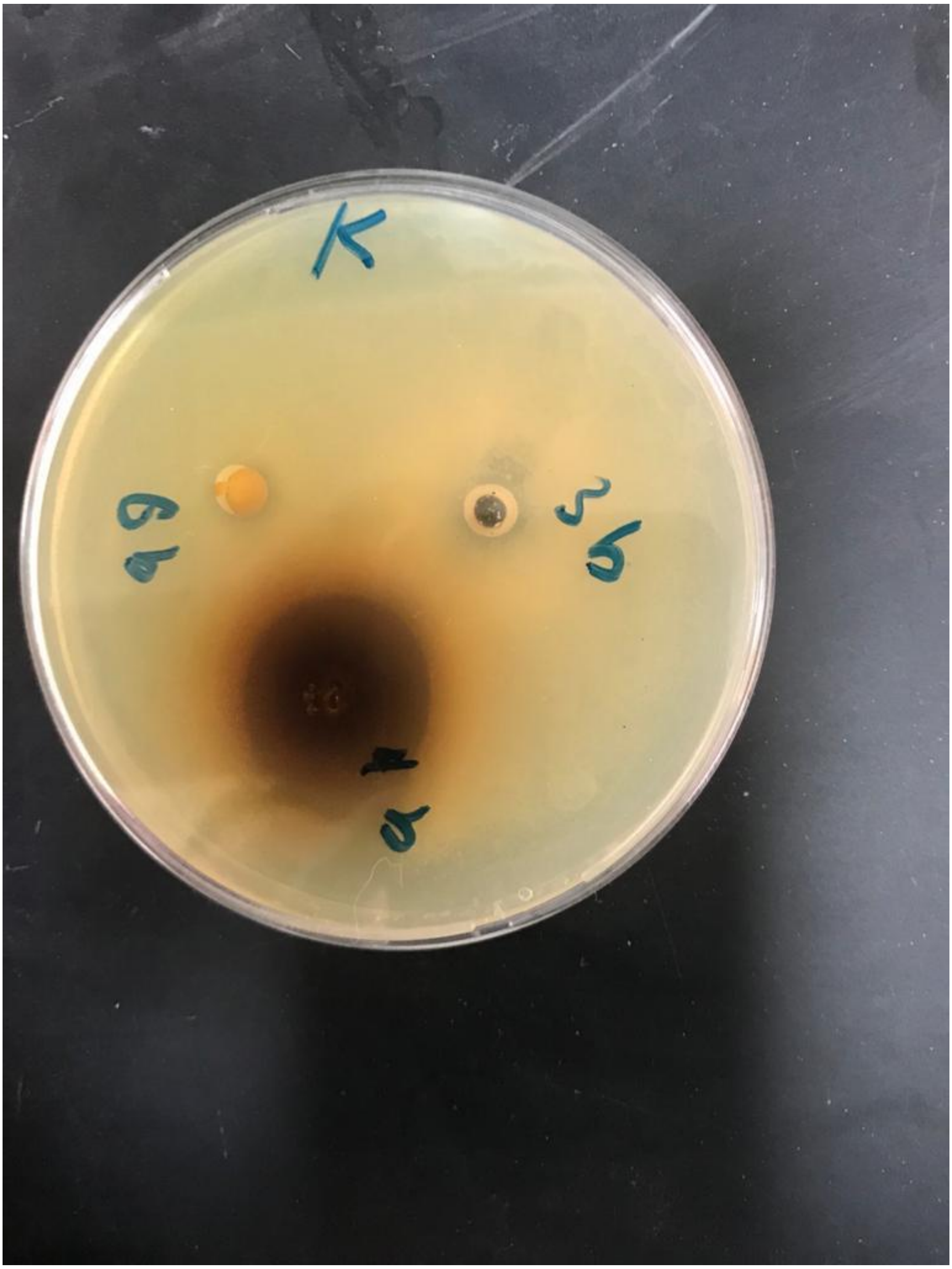

Fig 35. Antibacterial of 3b, 4b and 6b against *K. pneumoniae*.

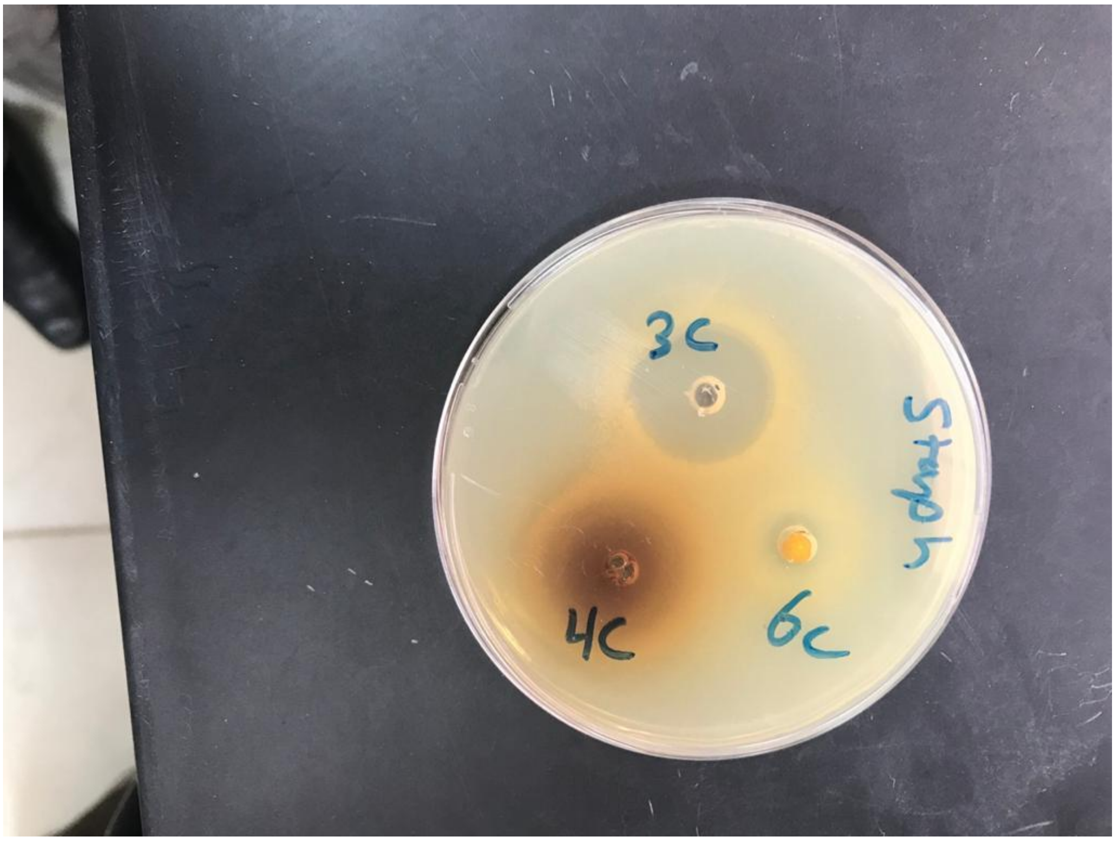

Fig 36. Antibacterial of 3c, 4c and 6c against *S. aureus*

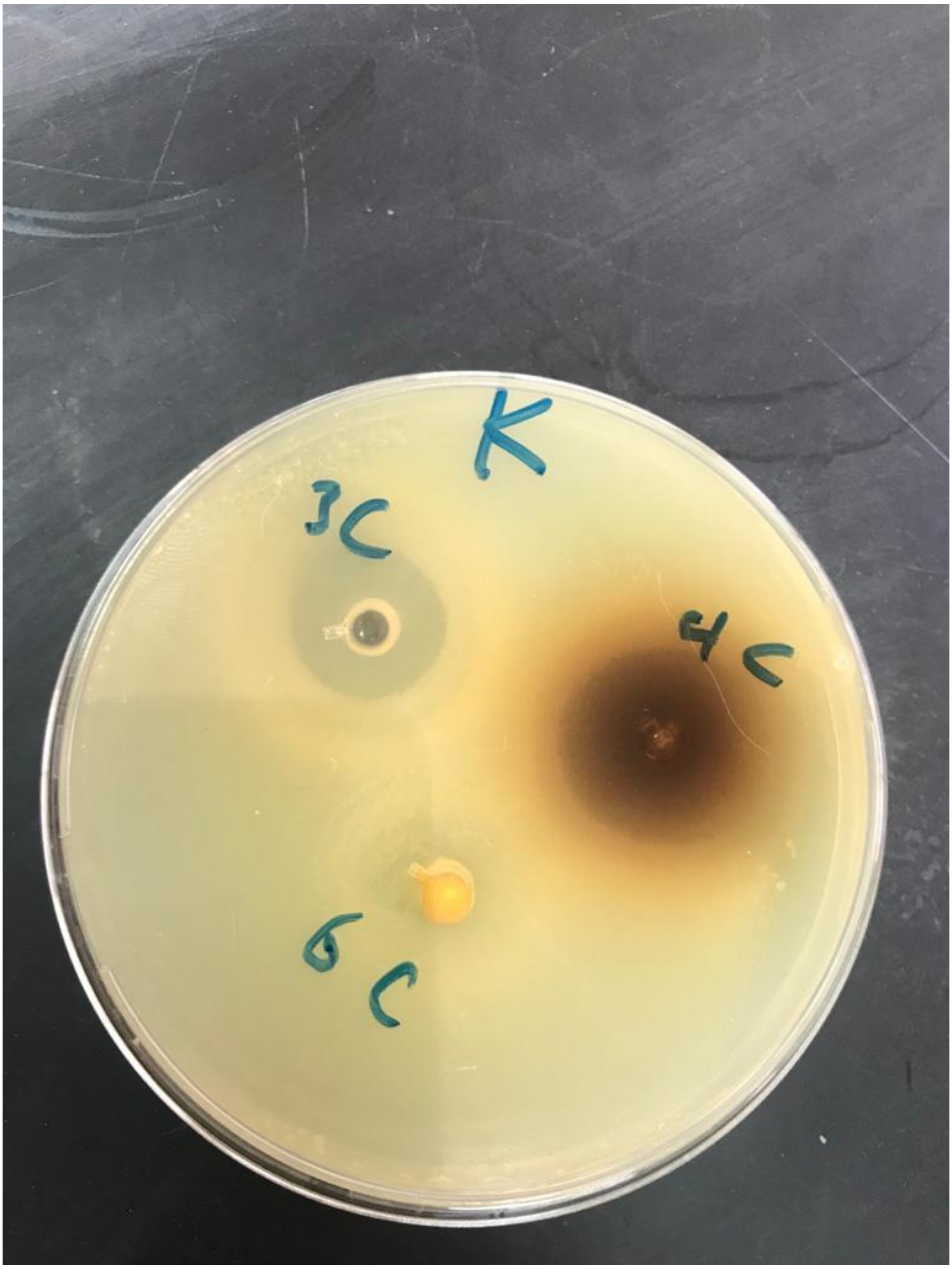

Fig 37. Antibacterial of 3c, 4c and 6c against *K. pneumoniae*.
