# Supplementary material for: Antibacterial and molecular docking studies of newly synthesized nucleosides and Schiff bases derived from sulfadimidines
Source: Sci Rep. 2021 Sep 9;11:17953. doi: 10.1038/s41598-021-97297-1 (PMC8429437; doi:10.1038/s41598-021-97297-1)
Supplement: Supplementary file 2 — Supplementary Information 2. [file 41598_2021_97297_MOESM2_ESM.pdf]

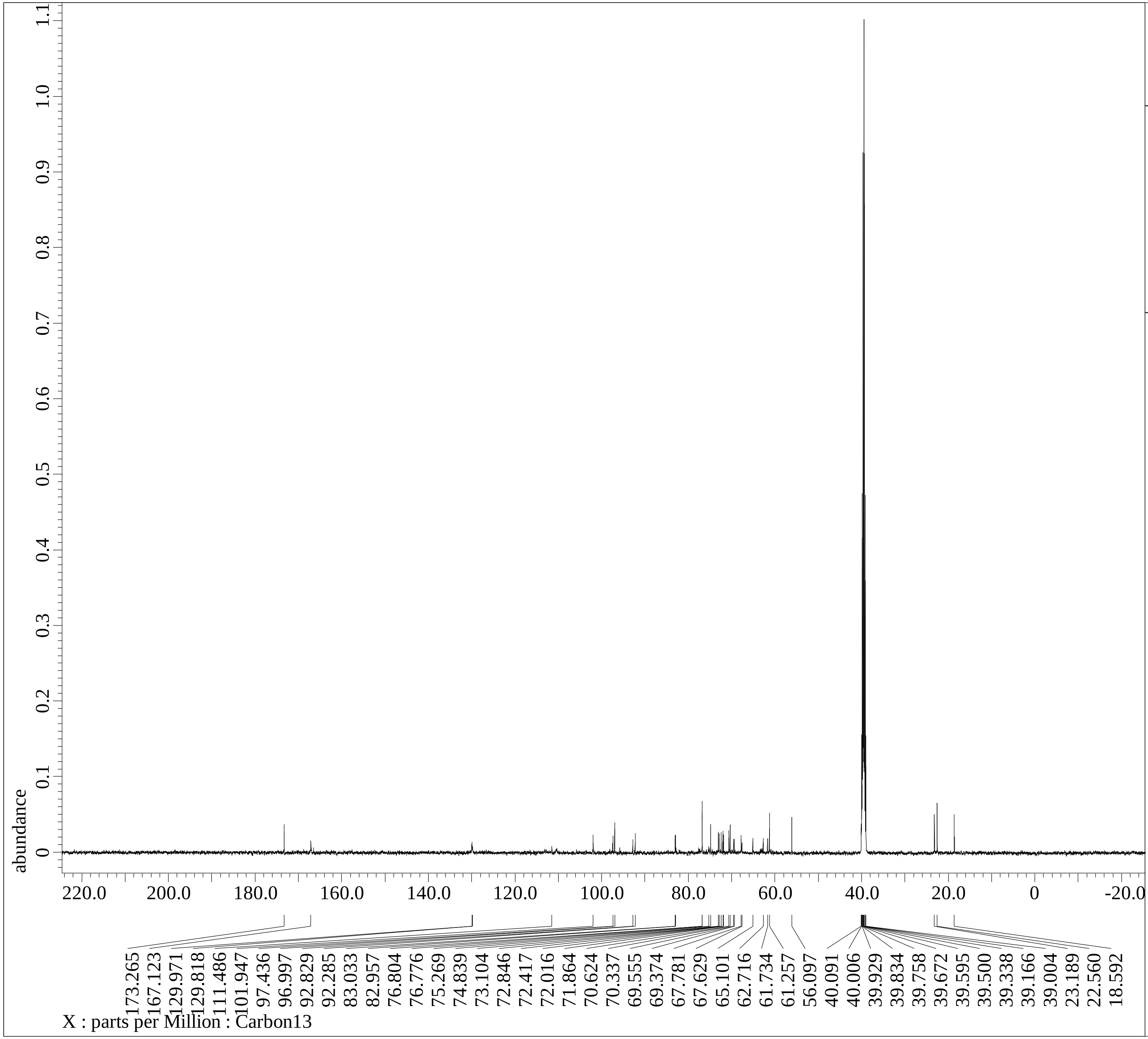


```
---- PROCESSING PARAMETERS ----
dc_balance( 0, FALSE )
sexp( 2.0[Hz], 0.0[s] )
trapezoid( 0[%], 0[%], 80[%], 100[%] )
zerofill( 1 )
fft( 1, TRUE, TRUE )
machinephase
ppm
phase( 0.20398, -76.57313, 73.97475[%] )
phase( 0, 16.60766, 74.00908[%] )
reference( 39.96578[ppm], 39.5[ppm] )

Derived from: E3a_carbon-1-1.jdf

Filename          = E3a_carbon-1-6.jdf
Author            = delta
Experiment        = carbon.jxp
Sample_Id         = E3a
Solvent           = DMSO-D6
Creation_Time     =  3-DEC-2020 14:30:20
Revision_Time     = 10-DEC-2020 12:30:46
Current_Time      = 10-DEC-2020 12:30:52

Comment           = single pulse decoupled gat
Data_Format       = 1D COMPLEX
Dim_Size          = 26214
Dim_Title         = Carbon13
Dim_Units         = [ppm]
Dimensions        = X
Site              = JNM-ECX500II
Spectrometer      = DELTA2_NMR

Field_Strength    = 11.7473579[T] (500[MHz])
X_Acq_Duration    = 0.83361792[s]
X_Domain          = 13C
X_Freq            = 125.76529768[MHz]
X_Offset          = 100[ppm]
X_Points          = 32768
X_Prescans        = 4
X_Resolution      = 1.19959034[Hz]
X_Sweep           = 39.3081761[kHz]
X_Sweep_Clipped   = 31.44654088[kHz]
Irr_Domain        = Proton
Irr_Freq          = 500.15991521[MHz]
Irr_Offset        = 5.0[ppm]
Clipped           = FALSE
Scans             = 1024
Total_Scans       = 1024

Relaxation_Delay  = 2[s]
Recvr_Gain        = 50
Temp_Get          = 23[dC]
X_90_Width        = 10[us]
X_Acq_Time        = 0.83361792[s]
X_Angle           = 30[deg]
X_Atn             = 12.1[dB]
X_Pulse           = 3.33333333[us]
Irr_Atn_Dec       = 25.569[dB]
Irr_Atn_Noe       = 25.569[dB]
Irr_Noise         = WALTZ
Irr_Pwidth        = 92[us]
Decoupling        = TRUE
```

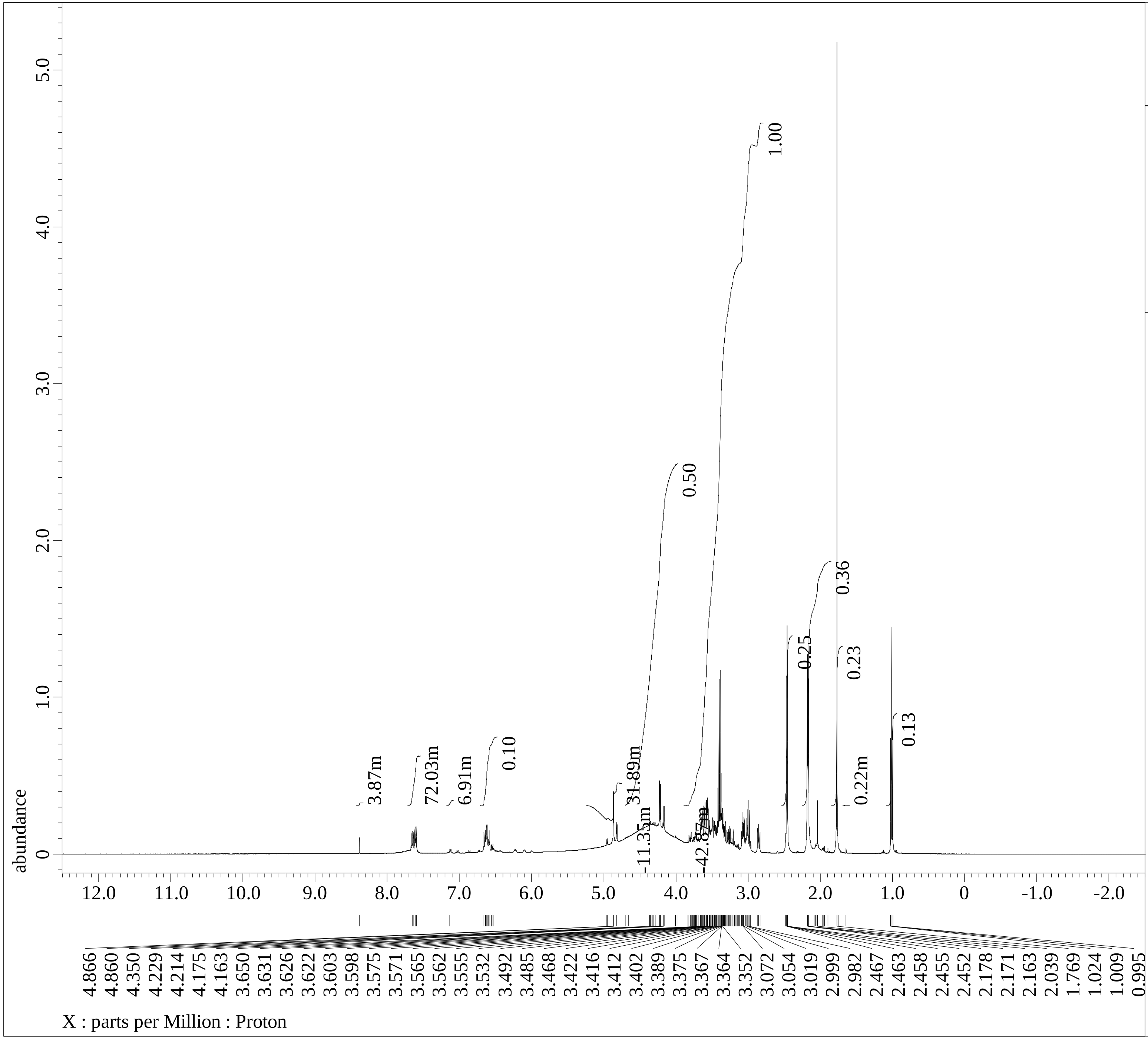

JEOL RESONANCE

```
---- PROCESSING PARAMETERS ----
dc_balance( 0, FALSE )
sexp( 0.2[Hz], 0.0[s] )
trapezoid( 0[%], 0[%], 80[%], 100[%] )
zerofill( 1 )
fft( 1, TRUE, TRUE )
machinephase
ppm
phase( 23.66171, -140.97499, 71.56264[%] )
phase( -36.69394, 121.49935, 71.53975[%] )

Filename          = E3a_proton-1-5.jdf
Author            = delta
Experiment        = proton.jxp
Sample_Id         = E3a
Solvent           = DMSO-D6
Creation_Time     =  3-DEC-2020 14:26:51
Revision_Time     = 10-DEC-2020 12:29:40
Current_Time      = 10-DEC-2020 12:29:47

Comment           = single_pulse
Data_Format       = 1D COMPLEX
Dim_Size          = 13107
Dim_Title         = Proton
Dim_Units         = [ppm]
Dimensions        = X
Site              = JNM-ECX500II
Spectrometer      = DELTA2_NMR

Field_Strength    = 11.7473579[T] (500[MHz])
X_Acq_Duration    = 1.74587904[s]
X_Domain          = 1H
X_Freq            = 500.15991521[MHz]
X_Offset          = 5.0[ppm]
X_Points          = 16384
X_Prescans        = 1
X_Resolution      = 0.57277737[Hz]
X_Sweep           = 9.38438438[kHz]
X_Sweep_Clipped   = 7.50750751[kHz]
Irr_Domain        = Proton
Irr_Freq          = 500.15991521[MHz]
Irr_Offset        = 5.0[ppm]
Tri_Domain        = Proton
Tri_Freq          = 500.15991521[MHz]
Tri_Offset        = 5.0[ppm]
Clipped           = FALSE
Scans             = 8
Total_Scans       = 8

Relaxation_Delay  = 5[s]
Recvr_Gain        = 30
Temp_Get          = 22.9[dC]
X_90_Width        = 7.25[us]
X_Acq_Time        = 1.74587904[s]
X_Angle           = 45[deg]
X_Atn             = 3.5[dB]
X_Pulse           = 3.625[us]
Irr_Mode          = Off
Tri_Mode          = Off
```

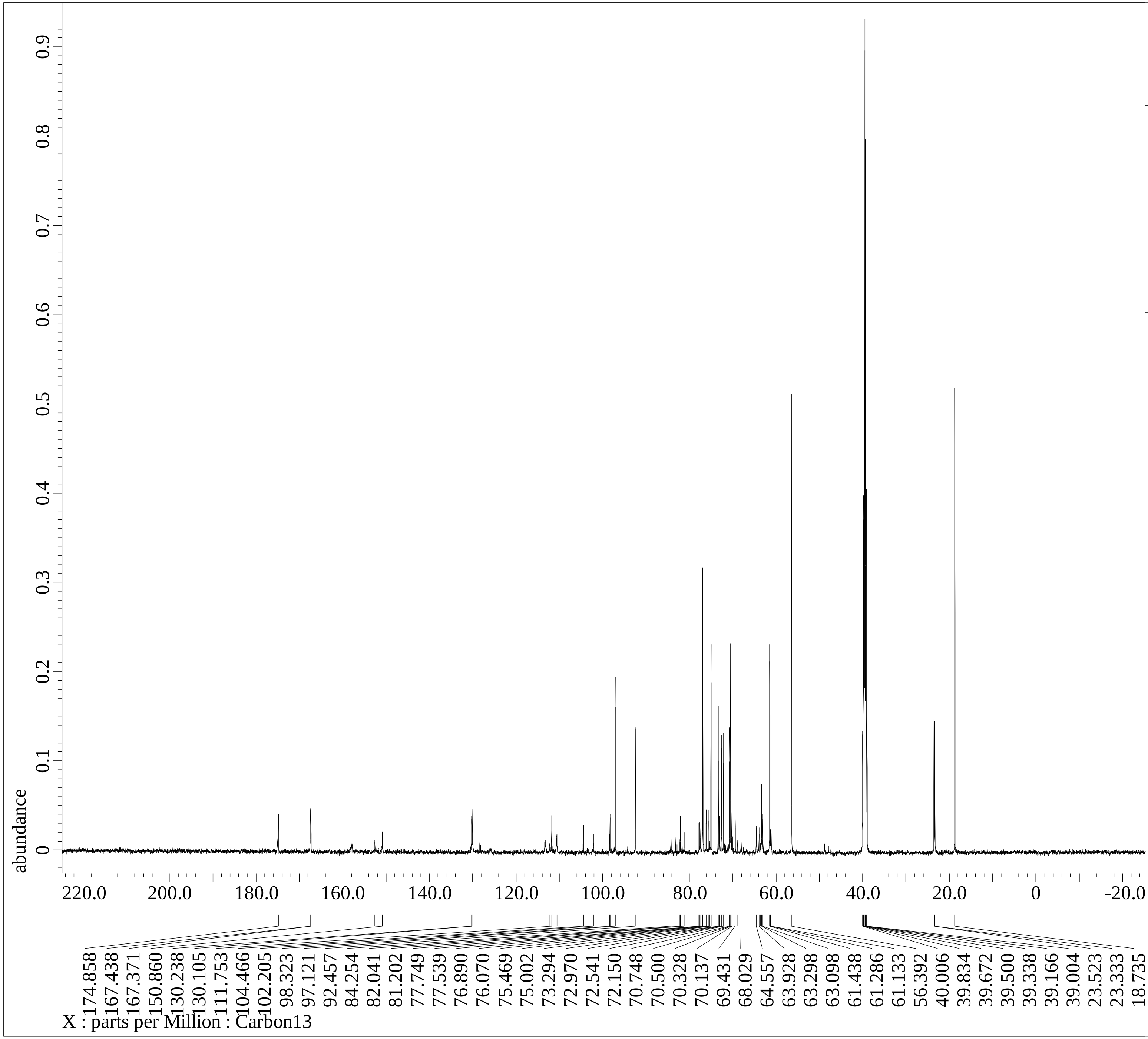

JEOL RESONANCE

```
---- PROCESSING PARAMETERS ----
dc_balance( 0, FALSE )
sexp( 2.0[Hz], 0.0[s] )
trapezoid( 0[%], 0[%], 80[%], 100[%] )
zerofill( 1 )
fft( 1, TRUE, TRUE )
machinephase
ppm
phase( 0.26604, -30.54604, 74.12734[%] )
phase( 0, 7.80309, 74.13879[%] )
reference( 39.72732[ppm], 39.5[ppm] )

Derived from: E3b_carbon-1-1.jdf
```

```
Filename          = E3b_carbon-1-6.jdf
Author            = console
Experiment        = carbon.jxp
Sample_Id         = E3b
Solvent           = DMSO-D6
Creation_Time     =  7-DEC-2020 13:10:29
Revision_Time     = 10-DEC-2020 12:33:22
Current_Time      = 10-DEC-2020 12:33:31

Comment           = single pulse decoupled gat
Data_Format       = 1D COMPLEX
Dim_Size          = 26214
Dim_Title         = Carbon13
Dim_Units         = [ppm]
Dimensions        = X
Site              = JNM-ECX500II
Spectrometer      = DELTA2_NMR

Field_Strength    = 11.7473579[T] (500[MHz])
X_Acq_Duration    = 0.83361792[s]
X_Domain          = 13C
X_Freq            = 125.76529768[MHz]
X_Offset          = 100[ppm]
X_Points          = 32768
X_Prescans        = 4
X_Resolution      = 1.19959034[Hz]
X_Sweep           = 39.3081761[kHz]
X_Sweep_Clipped   = 31.44654088[kHz]
Irr_Domain        = Proton
Irr_Freq          = 500.15991521[MHz]
Irr_Offset        = 5.0[ppm]
Clipped           = FALSE
Scans             = 1024
Total_Scans       = 1024

Relaxation_Delay  = 2[s]
Recvr_Gain        = 50
Temp_Get          = 21.7[dC]
X_90_Width        = 10[us]
X_Acq_Time        = 0.83361792[s]
X_Angle           = 30[deg]
X_Atn             = 12.1[dB]
X_Pulse           = 3.33333333[us]
Irr_Atn_Dec       = 25.569[dB]
Irr_Atn_Noe       = 25.569[dB]
Irr_Noise         = WALTZ
Irr_Pwidth        = 92[us]
Decoupling        = TRUE
```

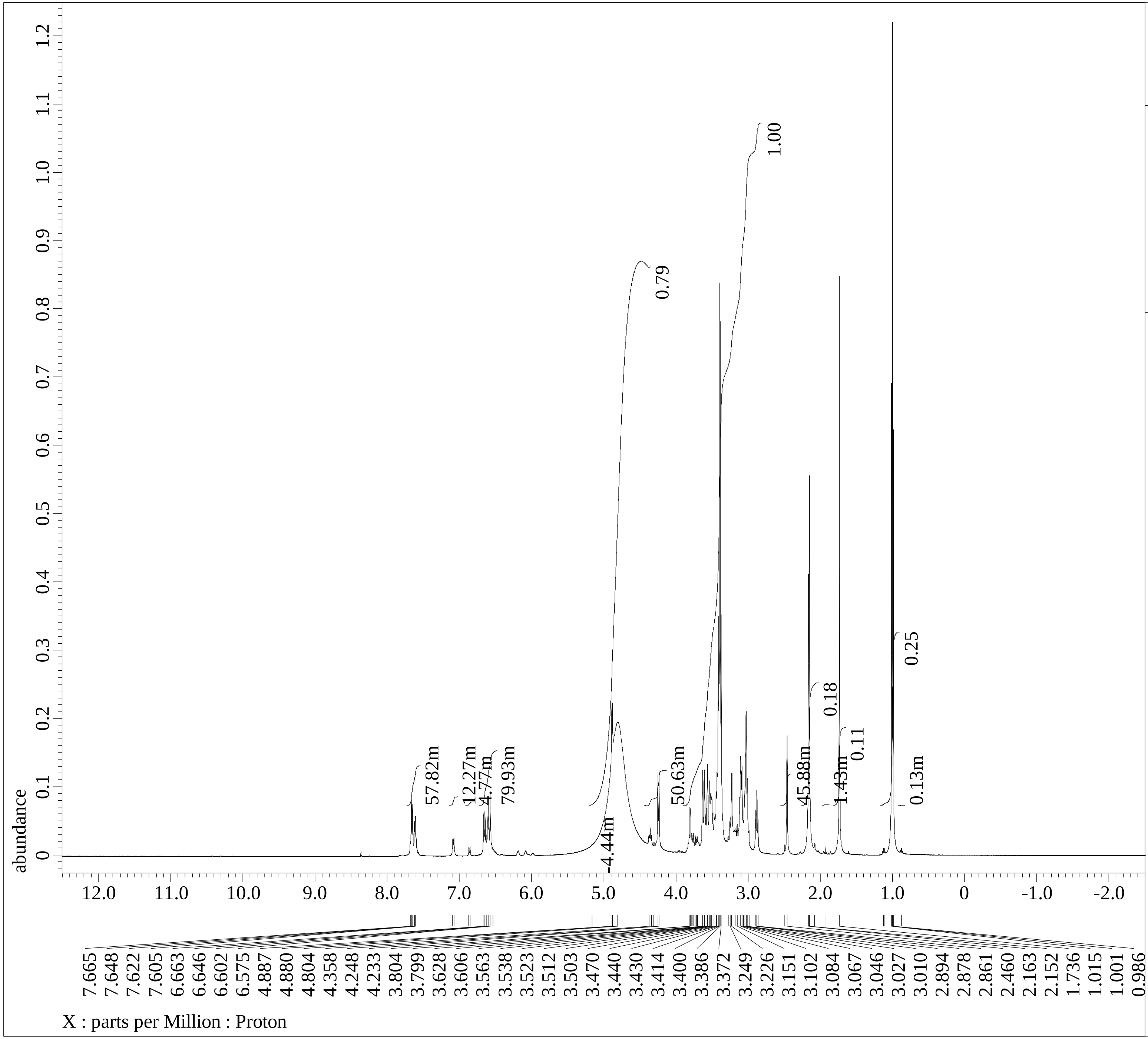

JEOL RESONANCE

```
---- PROCESSING PARAMETERS ----
dc_balance( 0, FALSE )
sexp( 0.2[Hz], 0.0[s] )
trapezoid( 0[%], 0[%], 80[%], 100[%] )
zerofill( 1 )
fft( 1, TRUE, TRUE )
machinephase
ppm
phase( 24.68218, -105.94234, 76.66718[%] )
phase( -24.07311, 106.4665, 76.67481[%] )

Filename           = E3b_proton-1-5.jdf
Author             = console
Experiment         = proton.jxp
Sample_Id          = E3b
Solvent            = DMSO-D6
Creation_Time      =  7-DEC-2020 13:06:59
Revision_Time      = 10-DEC-2020 12:32:20
Current_Time       = 10-DEC-2020 12:32:34

Comment            = single_pulse
Data_Format        = 1D COMPLEX
Dim_Size           = 13107
Dim_Title          = Proton
Dim_Units          = [ppm]
Dimensions         = X
Site               = JNM-ECX500II
Spectrometer       = DELTA2_NMR

Field_Strength     = 11.7473579[T] (500[MHz])
X_Acq_Duration     = 1.74587904[s]
X_Domain           = 1H
X_Freq             = 500.15991521[MHz]
X_Offset           = 5.0[ppm]
X_Points           = 16384
X_Prescans         = 1
X_Resolution       = 0.57277737[Hz]
X_Sweep            = 9.38438438[kHz]
X_Sweep_Clipped    = 7.50750751[kHz]
Irr_Domain         = Proton
Irr_Freq           = 500.15991521[MHz]
Irr_Offset         = 5.0[ppm]
Tri_Domain         = Proton
Tri_Freq           = 500.15991521[MHz]
Tri_Offset         = 5.0[ppm]
Clipped            = FALSE
Scans              = 8
Total_Scans        = 8

Relaxation_Delay   = 5[s]
Recvr_Gain         = 10
Temp_Get           = 21.6[dC]
X_90_Width         = 7.25[us]
X_Acq_Time         = 1.74587904[s]
X_Angle            = 45[deg]
X_Atn              = 3.5[dB]
X_Pulse            = 3.625[us]
Irr_Mode           = Off
Tri_Mode           = Off
```

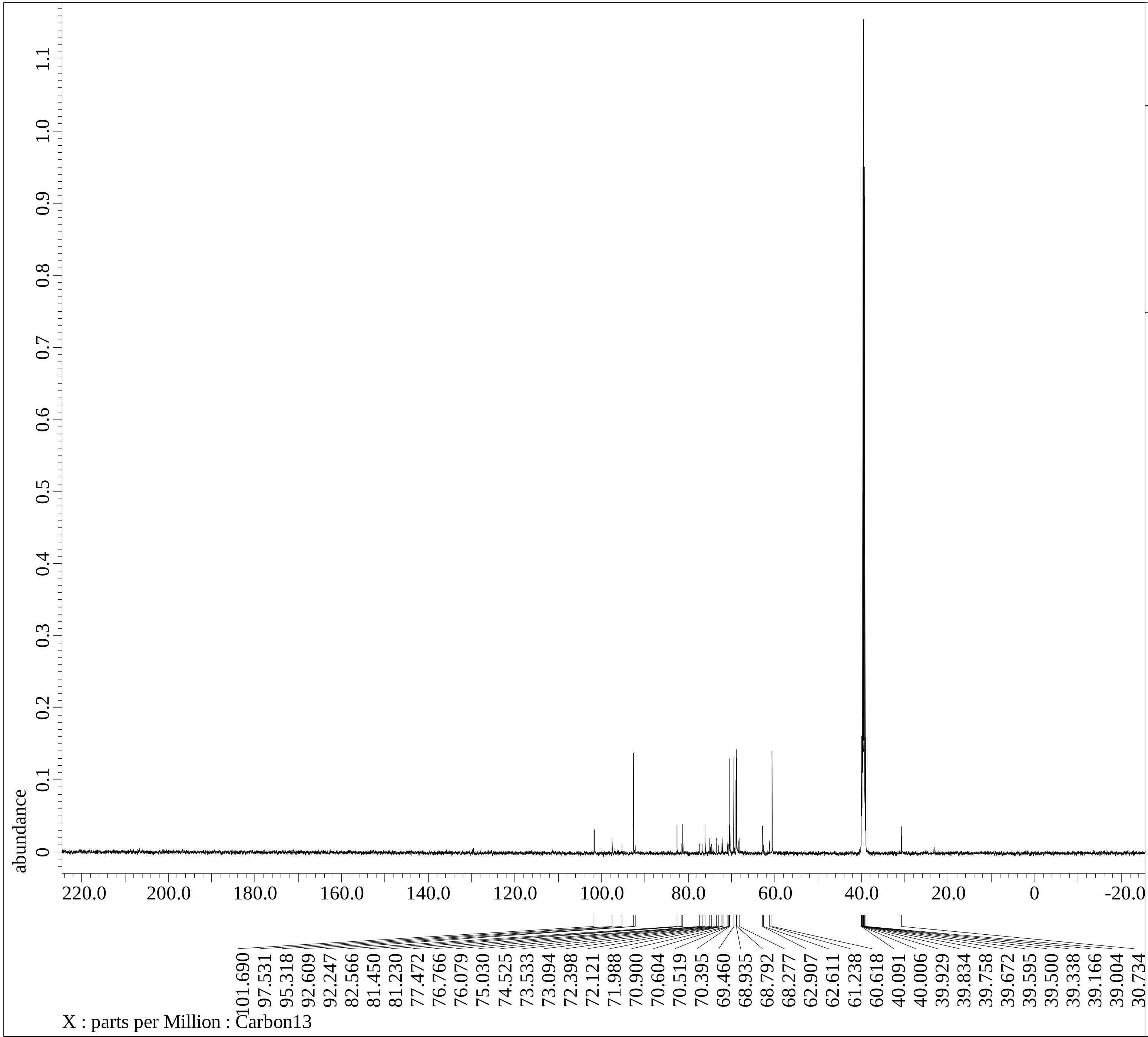

JEOL RESONANCE

```
---- PROCESSING PARAMETERS ----
dc_balance( 0, FALSE )
sexp( 2.0[Hz], 0.0[s] )
trapezoid( 0[%], 0[%], 80[%], 100[%] )
zerofill( 1 )
fft( 1, TRUE, TRUE )
machinephase
ppm
phase( 0.22122, 295.65778, 73.97856[%] )
phase( 0, -265.16274, 74.00908[%] )
reference( 39.99439[ppm], 39.5[ppm] )

Derived from: E3c_carbon-1-1.jdf
```

```
Filename          = E3c_carbon-1-6.jdf
Author            = delta
Experiment        = carbon.jxp
Sample_Id         = E3c
Solvent           = DMSO-D6
Creation_Time     =  3-DEC-2020 15:26:43
Revision_Time     = 10-DEC-2020 12:36:21
Current_Time      = 10-DEC-2020 12:36:27

Comment           = single pulse decoupled gat
Data_Format       = 1D COMPLEX
Dim_Size          = 26214
Dim_Title         = Carbon13
Dim_Units         = [ppm]
Dimensions        = X
Site              = JNM-ECX500II
Spectrometer      = DELTA2_NMR

Field_Strength    = 11.7473579[T] (500[MHz])
X_Acq_Duration    = 0.83361792[s]
X_Domain          = 13C
X_Freq            = 125.76529768[MHz]
X_Offset          = 100[ppm]
X_Points          = 32768
X_Prescans        = 4
X_Resolution      = 1.19959034[Hz]
X_Sweep           = 39.3081761[kHz]
X_Sweep_Clipped   = 31.44654088[kHz]
Irr_Domain        = Proton
Irr_Freq          = 500.15991521[MHz]
Irr_Offset        = 5.0[ppm]
Clipped           = FALSE
Scans             = 807
Total_Scans       = 807

Relaxation_Delay  = 2[s]
Recvr_Gain        = 50
Temp_Get          = 23[dC]
X_90_Width        = 10[us]
X_Acq_Time        = 0.83361792[s]
X_Angle           = 30[deg]
X_Atn             = 12.1[dB]
X_Pulse           = 3.33333333[us]
Irr_Atn_Dec       = 25.569[dB]
Irr_Atn_Noe       = 25.569[dB]
Irr_Noise         = WALTZ
Irr_Pwidth        = 92[us]
Decoupling        = TRUE
```

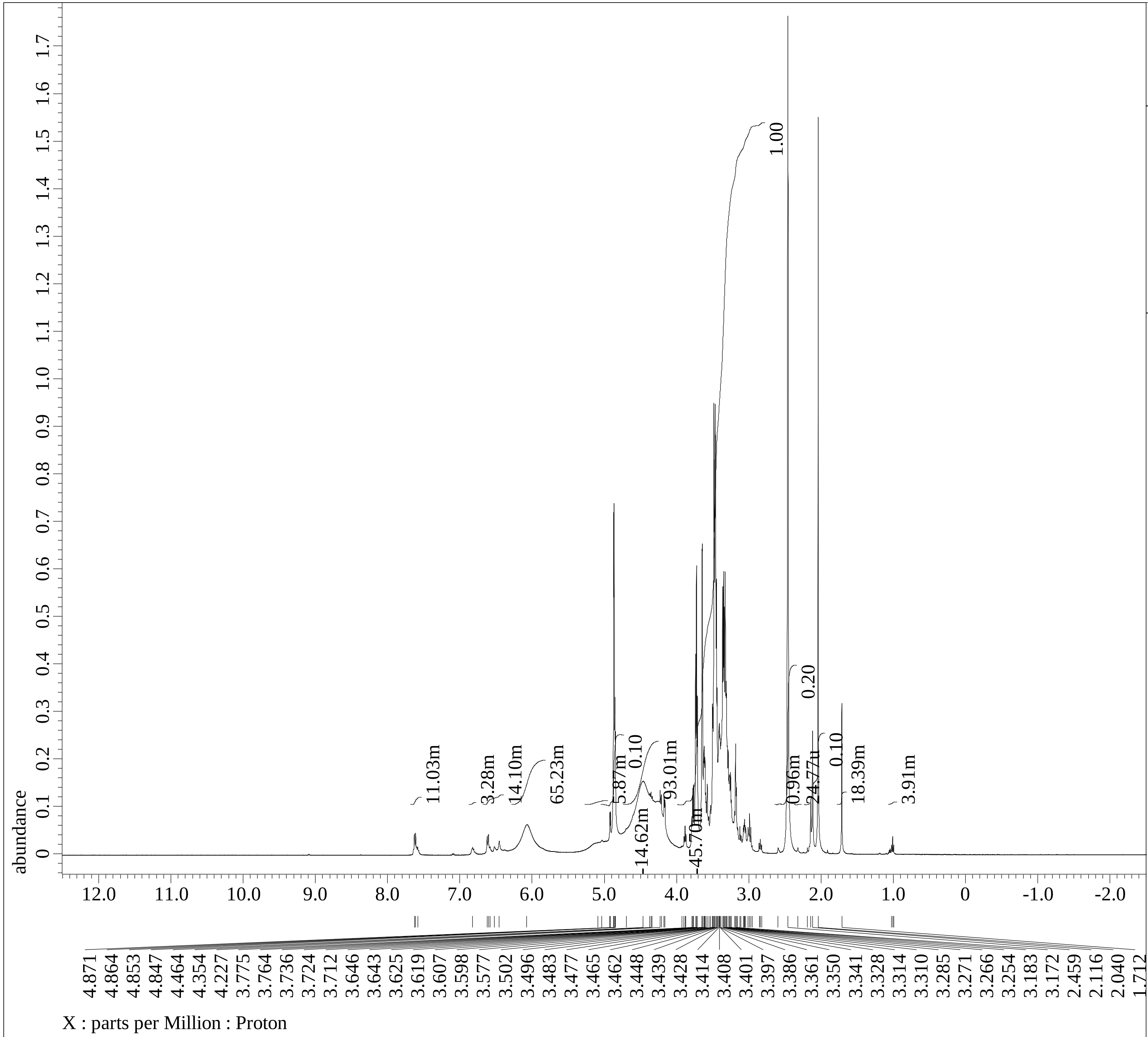

JEOL RESONANCE

```
---- PROCESSING PARAMETERS ----
dc_balance( 0, FALSE )
sexp( 0.2[Hz], 0.0[s] )
trapezoid( 0[%], 0[%], 80[%], 100[%] )
zerofill( 1 )
fft( 1, TRUE, TRUE )
machinephase
ppm
phase( -6.57038, 158.32427, 66.93881[%] )
phase( -1.21132, -154.48699, 66.99222[%] )
```

```
Filename          = E3c_proton-1-5.jdf
Author            = delta
Experiment        = proton.jxp
Sample_Id         = E3c
Solvent           = DMSO-D6
Creation_Time     =  3-DEC-2020 15:23:12
Revision_Time     = 10-DEC-2020 12:35:18
Current_Time      = 10-DEC-2020 12:35:26

Comment           = single_pulse
Data_Format       = 1D COMPLEX
Dim_Size          = 13107
Dim_Title         = Proton
Dim_Units         = [ppm]
Dimensions        = X
Site              = JNM-ECX500II
Spectrometer      = DELTA2_NMR

Field_Strength    = 11.7473579[T] (500[MHz])
X_Acq_Duration    = 1.74587904[s]
X_Domain          = 1H
X_Freq            = 500.15991521[MHz]
X_Offset          = 5.0[ppm]
X_Points          = 16384
X_Prescans        = 1
X_Resolution      = 0.57277737[Hz]
X_Sweep           = 9.38438438[kHz]
X_Sweep_Clipped   = 7.50750751[kHz]
Irr_Domain        = Proton
Irr_Freq          = 500.15991521[MHz]
Irr_Offset        = 5.0[ppm]
Tri_Domain        = Proton
Tri_Freq          = 500.15991521[MHz]
Tri_Offset        = 5.0[ppm]
Clipped           = FALSE
Scans             = 8
Total_Scans       = 8

Relaxation_Delay  = 5[s]
Recvr_Gain        = 30
Temp_Get          = 22.9[dC]
X_90_Width        = 7.25[us]
X_Acq_Time        = 1.74587904[s]
X_Angle           = 45[deg]
X_Atn             = 3.5[dB]
X_Pulse           = 3.625[us]
Irr_Mode          = Off
Tri_Mode          = Off
```

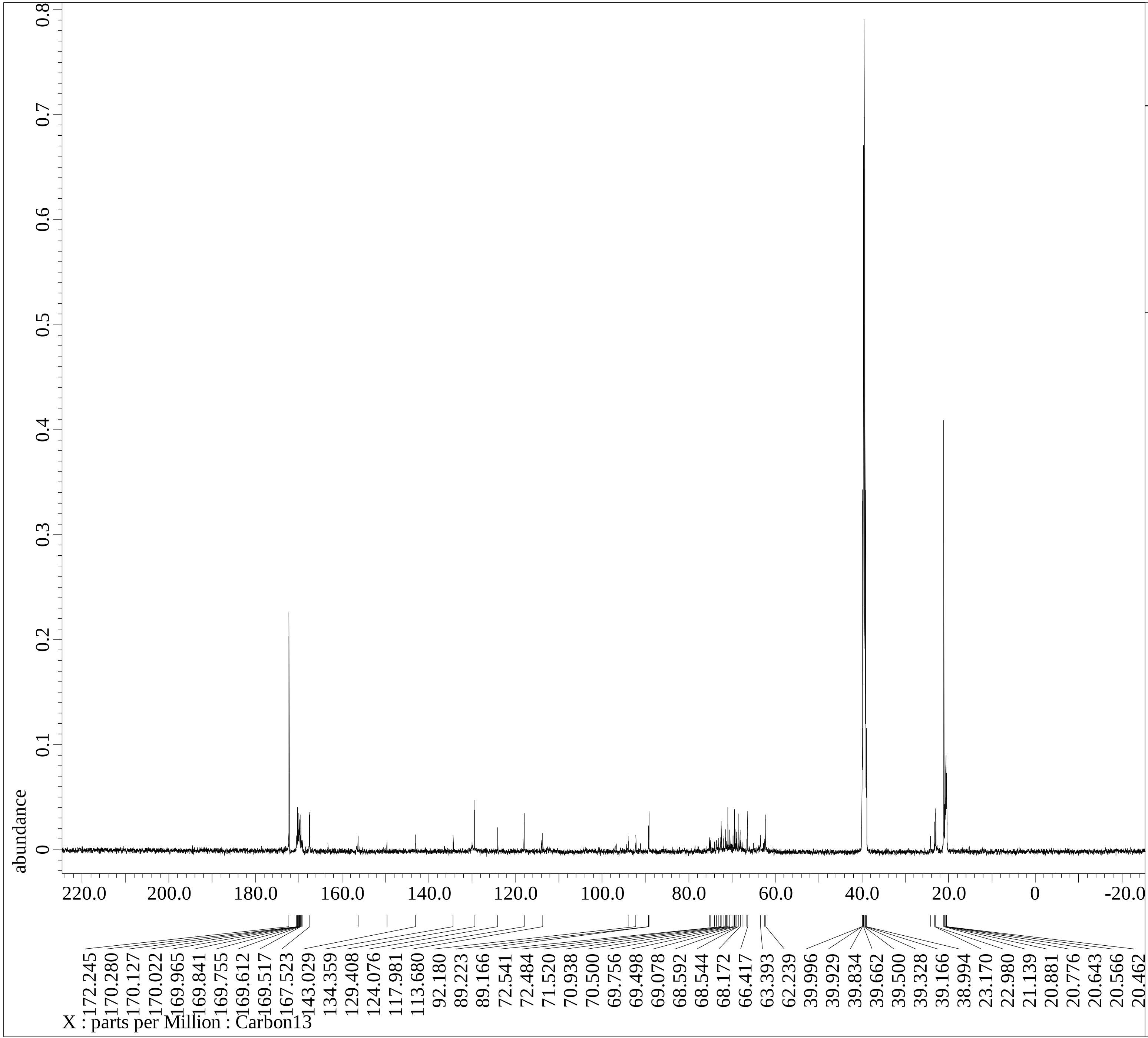

JEOL RESONANCE

```
---- PROCESSING PARAMETERS ----
dc_balance( 0, FALSE )
sexp( 2.0[Hz], 0.0[s] )
trapezoid( 0[%], 0[%], 80[%], 100[%] )
zerofill( 1 )
fft( 1, TRUE, TRUE )
machinephase
ppm
phase( -0.02248, 18.97299, 74.05104[%] )
phase( 0, -47.61016, 74.00908[%] )
reference( 39.8704[ppm], 39.5[ppm] )
```

```
Filename          = E4b_carbon-1-6.jdf
Author            = console
Experiment        = carbon.jxp
Sample_Id         = E4b
Solvent           = DMSO-D6
Creation_Time     =  7-DEC-2020 16:57:06
Revision_Time     = 10-DEC-2020 12:39:44
Current_Time      = 10-DEC-2020 12:47:30

Comment           = single pulse decoupled gat
Data_Format       = 1D COMPLEX
Dim_Size          = 26214
Dim_Title         = Carbon13
Dim_Units         = [ppm]
Dimensions        = X
Site              = JNM-ECX500II
Spectrometer      = DELTA2_NMR

Field_Strength    = 11.7473579[T] (500[MHz])
X_Acq_Duration    = 0.83361792[s]
X_Domain          = 13C
X_Freq            = 125.76529768[MHz]
X_Offset          = 100[ppm]
X_Points          = 32768
X_Prescans        = 4
X_Resolution      = 1.19959034[Hz]
X_Sweep           = 39.3081761[kHz]
X_Sweep_Clipped   = 31.44654088[kHz]
Irr_Domain        = Proton
Irr_Freq          = 500.15991521[MHz]
Irr_Offset        = 5.0[ppm]
Clipped           = FALSE
Scans             = 1024
Total_Scans       = 1024

Relaxation_Delay  = 2[s]
Recvr_Gain        = 50
Temp_Get          = 21.9[dC]
X_90_Width        = 10[us]
X_Acq_Time        = 0.83361792[s]
X_Angle           = 30[deg]
X_Atn             = 12.1[dB]
X_Pulse           = 3.33333333[us]
Irr_Atn_Dec       = 25.569[dB]
Irr_Atn_Noe       = 25.569[dB]
Irr_Noise         = WALTZ
Irr_Pwidth        = 92[us]
Decoupling        = TRUE
```

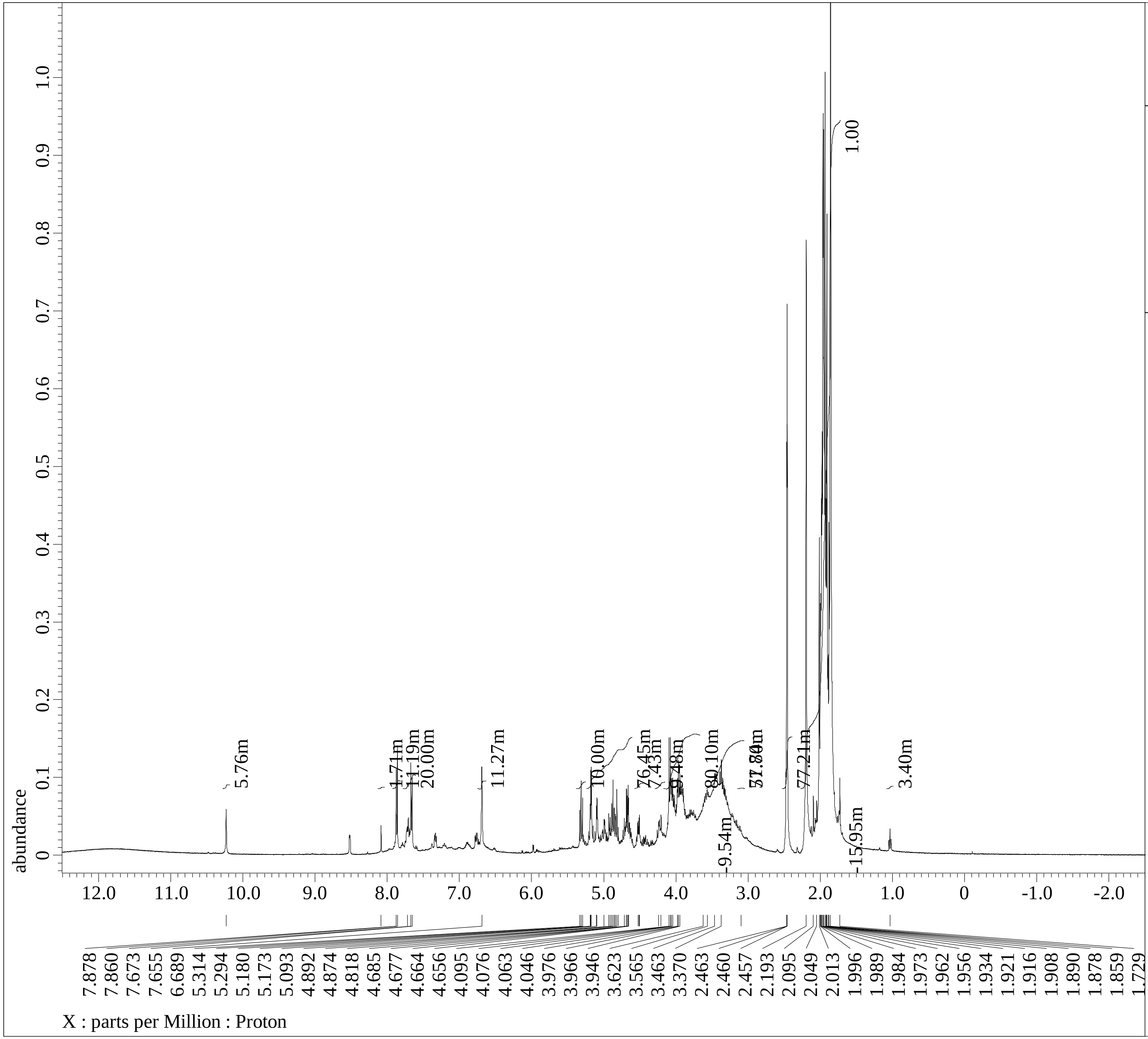

JEOL RESONANCE

```
---- PROCESSING PARAMETERS ----
dc_balance( 0, FALSE )
sexp( 0.2[Hz], 0.0[s] )
trapezoid( 0[%], 0[%], 80[%], 100[%] )
zerofill( 1 )
fft( 1, TRUE, TRUE )
machinephase
ppm
phase( -1.01699, 48.63758, 70.5631[%] )
phase( 0, -64.37605, 70.89119[%] )

Filename          = E4b_proton-1-4.jdf
Author            = console
Experiment        = proton.jxp
Sample_Id         = E4b
Solvent           = DMSO-D6
Creation_Time     =  7-DEC-2020 16:53:38
Revision_Time     = 10-DEC-2020 12:38:33
Current_Time      = 10-DEC-2020 12:38:50

Comment           = single_pulse
Data_Format       = 1D COMPLEX
Dim_Size          = 13107
Dim_Title         = Proton
Dim_Units         = [ppm]
Dimensions        = X
Site              = JNM-ECX500II
Spectrometer      = DELTA2_NMR

Field_Strength    = 11.7473579[T] (500[MHz])
X_Acq_Duration    = 1.74587904[s]
X_Domain          = 1H
X_Freq            = 500.15991521[MHz]
X_Offset          = 5.0[ppm]
X_Points          = 16384
X_Prescans        = 1
X_Resolution      = 0.57277737[Hz]
X_Sweep           = 9.38438438[kHz]
X_Sweep_Clipped   = 7.50750751[kHz]
Irr_Domain        = Proton
Irr_Freq          = 500.15991521[MHz]
Irr_Offset        = 5.0[ppm]
Tri_Domain        = Proton
Tri_Freq          = 500.15991521[MHz]
Tri_Offset        = 5.0[ppm]
Clipped           = FALSE
Scans             = 8
Total_Scans       = 8

Relaxation_Delay  = 5[s]
Recvr_Gain        = 20
Temp_Get          = 21.9[dC]
X_90_Width        = 7.25[us]
X_Acq_Time        = 1.74587904[s]
X_Angle           = 45[deg]
X_Atn             = 3.5[dB]
X_Pulse           = 3.625[us]
Irr_Mode          = Off
Tri_Mode          = Off
```

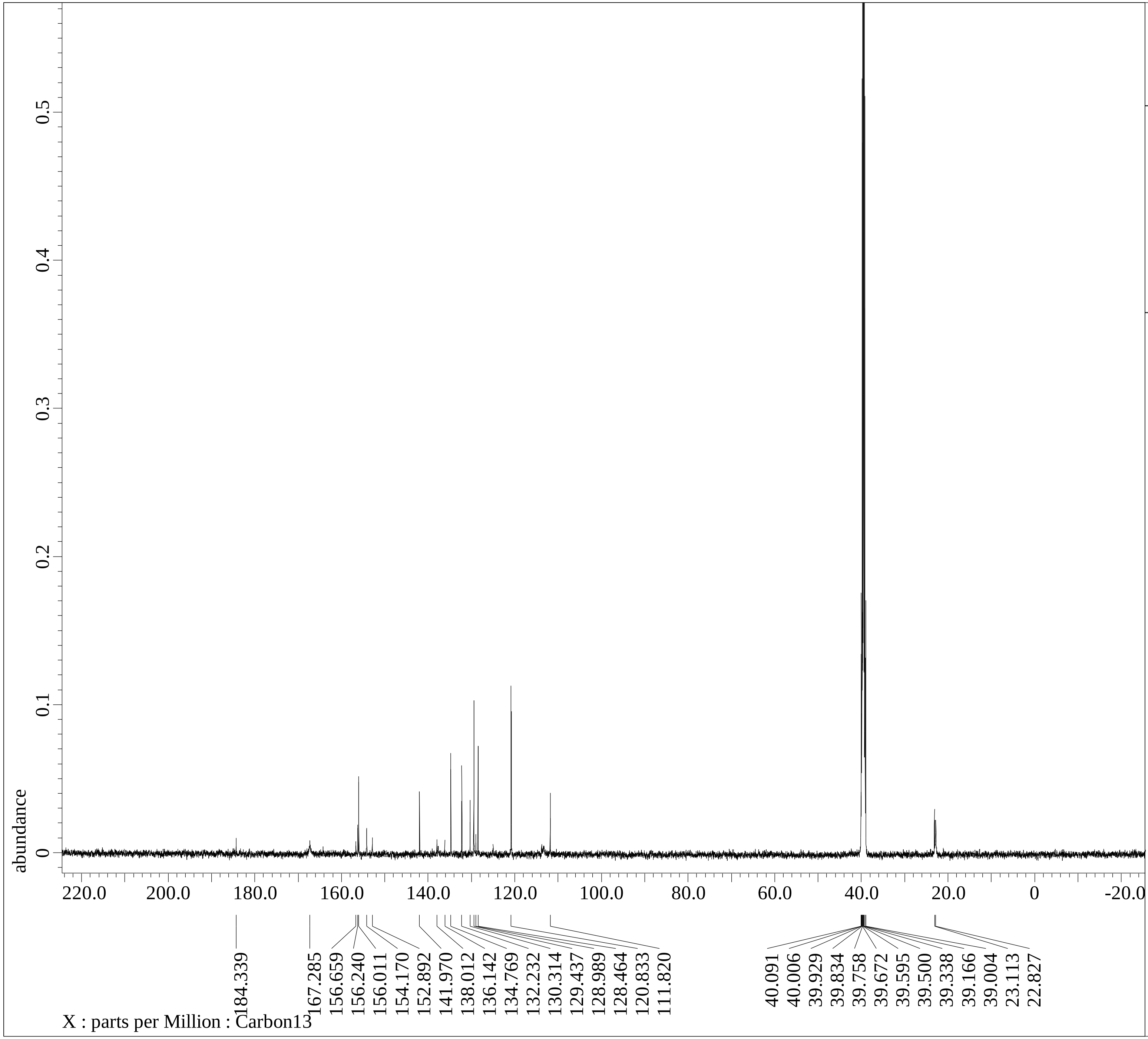

JEOL RESONANCE

```
---- PROCESSING PARAMETERS ----
dc_balance( 0, FALSE )
sexp( 2.0[Hz], 0.0[s] )
trapezoid( 0[%], 0[%], 80[%], 100[%] )
zerofill( 1 )
fft( 1, TRUE, TRUE )
machinephase
ppm
phase( -0.15528, 64.36721, 73.96712[%] )
phase( 0, -76.64628, 73.94423[%] )
reference( 40.02301[ppm], 39.5[ppm] )

Derived from: E6a_carbon-1-1.jdf
```

```
Filename          = E6a_carbon-1-6.jdf
Author            = console
Experiment        = carbon.jxp
Sample_Id         = E6a
Solvent           = DMSO-D6
Creation_Time     =  7-DEC-2020 17:53:10
Revision_Time     = 10-DEC-2020 12:52:20
Current_Time      = 10-DEC-2020 12:52:28

Comment           = single pulse decoupled gat
Data_Format       = 1D COMPLEX
Dim_Size          = 26214
Dim_Title         = Carbon13
Dim_Units         = [ppm]
Dimensions        = X
Site              = JNM-ECX500II
Spectrometer      = DELTA2_NMR

Field_Strength    = 11.7473579[T] (500[MHz])
X_Acq_Duration    = 0.83361792[s]
X_Domain          = 13C
X_Freq            = 125.76529768[MHz]
X_Offset          = 100[ppm]
X_Points          = 32768
X_Prescans        = 4
X_Resolution      = 1.19959034[Hz]
X_Sweep           = 39.3081761[kHz]
X_Sweep_Clipped   = 31.44654088[kHz]
Irr_Domain        = Proton
Irr_Freq          = 500.15991521[MHz]
Irr_Offset        = 5.0[ppm]
Clipped           = FALSE
Scans             = 1024
Total_Scans       = 1024

Relaxation_Delay  = 2[s]
Recvr_Gain        = 50
Temp_Get          = 21.9[dC]
X_90_Width        = 10[us]
X_Acq_Time        = 0.83361792[s]
X_Angle           = 30[deg]
X_Atn             = 12.1[dB]
X_Pulse           = 3.33333333[us]
Irr_Atn_Dec       = 25.569[dB]
Irr_Atn_Noe       = 25.569[dB]
Irr_Noise         = WALTZ
Irr_Pwidth        = 92[us]
Decoupling        = TRUE
```

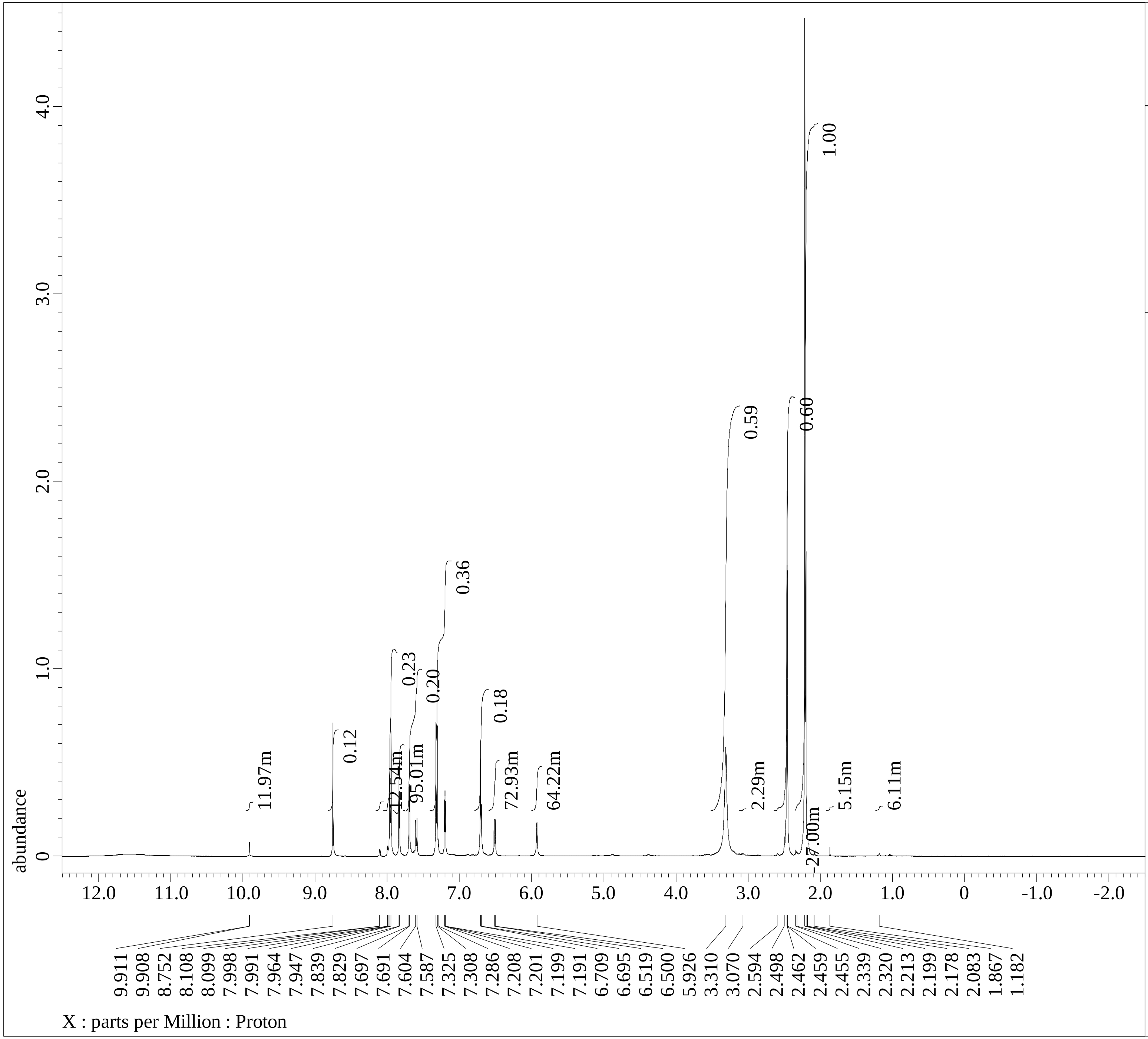

JEOL RESONANCE

---- PROCESSING PARAMETERS ----
dc_balance(0, FALSE)
sexp(0.2[Hz], 0.0[s])
trapezoid(0[%], 0[%], 80[%], 100[%])
zerofill(1)
fft(1, TRUE, TRUE)
machinephase
ppm
phase(4.49591, -59.29973, 68.65558[%])
phase(-4.11115, 56.70101, 68.61743[%])
phase(-4.38913, 49.15049, 68.54876[%])
phase(2.64763, -40.52147, 68.61743[%])

Filename = E6a_proton-1-8.jdf
Author = console
Experiment = proton.jxp
Sample_Id = E6a
Solvent = DMSO-D6
Creation_Time = 7-DEC-2020 17:49:41
Revision_Time = 10-DEC-2020 12:51:22
Current_Time = 10-DEC-2020 12:51:28

Comment = single_pulse
Data_Format = 1D COMPLEX
Dim_Size = 13107
Dim_Title = Proton
Dim_Units = [ppm]
Dimensions = X
Site = JNM-ECX500II
Spectrometer = DELTA2_NMR

Field_Strength = 11.7473579[T] (500[MHz])
X_Acq_Duration = 1.74587904[s]
X_Domain = 1H
X_Freq = 500.15991521[MHz]
X_Offset = 5.0[ppm]
X_Points = 16384
X_Prescans = 1
X_Resolution = 0.57277737[Hz]
X_Sweep = 9.38438438[kHz]
X_Sweep_Clipped = 7.50750751[kHz]
Irr_Domain = Proton
Irr_Freq = 500.15991521[MHz]
Irr_Offset = 5.0[ppm]
Tri_Domain = Proton
Tri_Freq = 500.15991521[MHz]
Tri_Offset = 5.0[ppm]
Clipped = FALSE
Scans = 8
Total_Scans = 8

Relaxation_Delay = 5[s]
Recvr_Gain = 30
Temp_Get = 21.9[dC]
X_90_Width = 7.25[us]
X_Acq_Time = 1.74587904[s]
X_Angle = 45[deg]
X_Atn = 3.5[dB]
X_Pulse = 3.625[us]
Irr_Mode = Off
Tri_Mode = Off

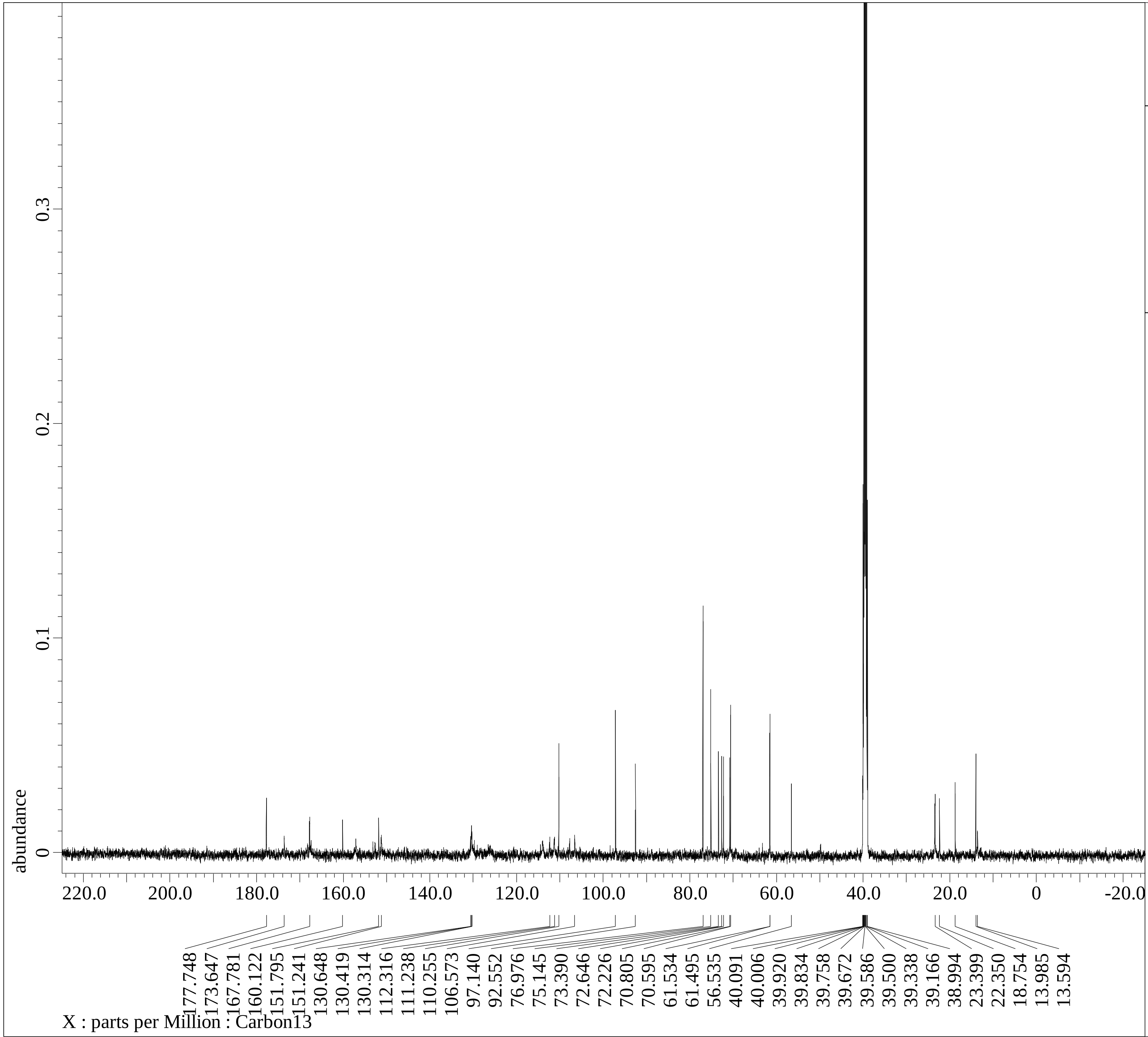

JEOL RESONANCE

```
---- PROCESSING PARAMETERS ----
dc_balance( 0, FALSE )
sexp( 2.0[Hz], 0.0[s] )
trapezoid( 0[%], 0[%], 80[%], 100[%] )
zerofill( 1 )
fft( 1, TRUE, TRUE )
machinephase
ppm
phase( 0.23642, -129.12198, 74.13116[%] )
phase( 0, 107.03624, 74.13879[%] )
reference( 39.61286[ppm], 39.5[ppm] )

Derived from: E6b_carbon-1-1.jdf
```

```
Filename          = E6b_carbon-1-6.jdf
Author            = console
Experiment        = carbon.jxp
Sample_Id         = E6b
Solvent           = DMSO-D6
Creation_Time     =  7-DEC-2020 18:49:27
Revision_Time     = 10-DEC-2020 12:49:06
Current_Time      = 10-DEC-2020 12:49:20

Comment           = single pulse decoupled gat
Data_Format       = 1D COMPLEX
Dim_Size          = 26214
Dim_Title         = Carbon13
Dim_Units         = [ppm]
Dimensions        = X
Site              = JNM-ECX500II
Spectrometer      = DELTA2_NMR

Field_Strength    = 11.7473579[T] (500[MHz])
X_Acq_Duration    = 0.83361792[s]
X_Domain          = 13C
X_Freq            = 125.76529768[MHz]
X_Offset          = 100[ppm]
X_Points          = 32768
X_Prescans        = 4
X_Resolution      = 1.19959034[Hz]
X_Sweep           = 39.3081761[kHz]
X_Sweep_Clipped   = 31.44654088[kHz]
Irr_Domain        = Proton
Irr_Freq          = 500.15991521[MHz]
Irr_Offset        = 5.0[ppm]
Clipped           = FALSE
Scans             = 1024
Total_Scans       = 1024

Relaxation_Delay  = 2[s]
Recvr_Gain        = 50
Temp_Get          = 21.9[dC]
X_90_Width        = 10[us]
X_Acq_Time        = 0.83361792[s]
X_Angle           = 30[deg]
X_Atn             = 12.1[dB]
X_Pulse           = 3.33333333[us]
Irr_Atn_Dec       = 25.569[dB]
Irr_Atn_Noe       = 25.569[dB]
Irr_Noise         = WALTZ
Irr_Pwidth        = 92[us]
Decoupling        = TRUE
```

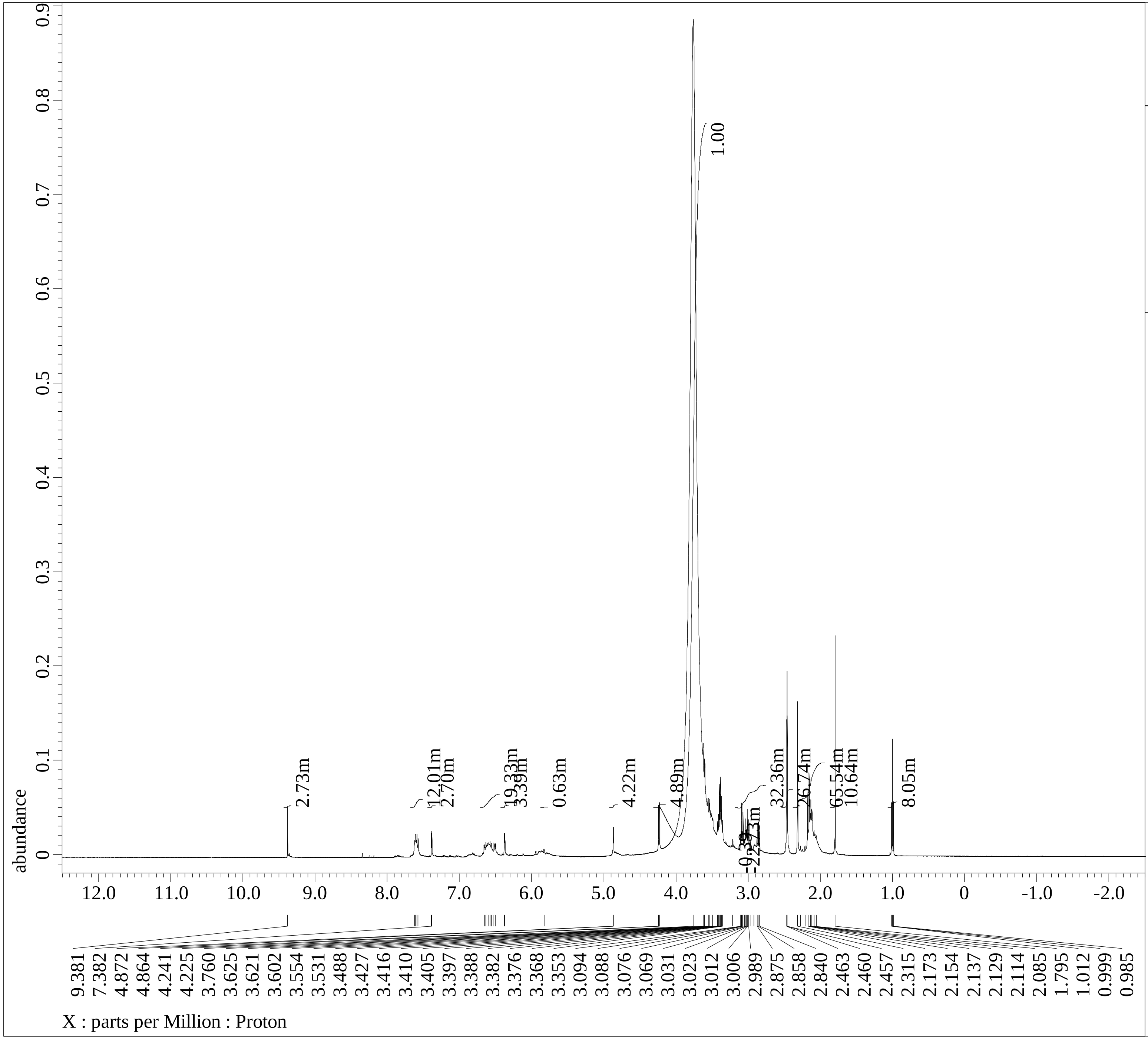

---- PROCESSING PARAMETERS ----
dc_balance(0, FALSE)
sexp(0.2[Hz], 0.0[s])
trapezoid(0[%], 0[%], 80[%], 100[%])
zerofill(1)
fft(1, TRUE, TRUE)
machinephase
ppm
phase(2.04788, -100.31889, 60.1175[%])
phase(0, 121.63024, 58.21761[%])

Filename	= E6b_proton-1-4.jdf
Author	= console
Experiment	= proton.jxp
Sample_Id	= E6b
Solvent	= DMSO-D6
Creation_Time	= 7-DEC-2020 18:45:58
Revision_Time	= 10-DEC-2020 12:53:08
Current_Time	= 10-DEC-2020 12:53:19
Comment	= single_pulse
Data_Format	= 1D COMPLEX
Dim_Size	= 13107
Dim_Title	= Proton
Dim_Units	= [ppm]
Dimensions	= X
Site	= JNM-ECX500II
Spectrometer	= DELTA2_NMR
Field_Strength	= 11.7473579[T] (500[MHz])
X_Acq_Duration	= 1.74587904[s]
X_Domain	= 1H
X_Freq	= 500.15991521[MHz]
X_Offset	= 5.0[ppm]
X_Points	= 16384
X_Prescans	= 1
X_Resolution	= 0.57277737[Hz]
X_Sweep	= 9.38438438[kHz]
X_Sweep_Clipped	= 7.50750751[kHz]
Irr_Domain	= Proton
Irr_Freq	= 500.15991521[MHz]
Irr_Offset	= 5.0[ppm]
Tri_Domain	= Proton
Tri_Freq	= 500.15991521[MHz]
Tri_Offset	= 5.0[ppm]
Clipped	= FALSE
Scans	= 8
Total_Scans	= 8
Relaxation_Delay	= 5[s]
Recvr_Gain	= 10
Temp_Get	= 21.9[dC]
X_90_Width	= 7.25[us]
X_Acq_Time	= 1.74587904[s]
X_Angle	= 45[deg]
X_Atn	= 3.5[dB]
X_Pulse	= 3.625[us]
Irr_Mode	= Off
Tri_Mode	= Off

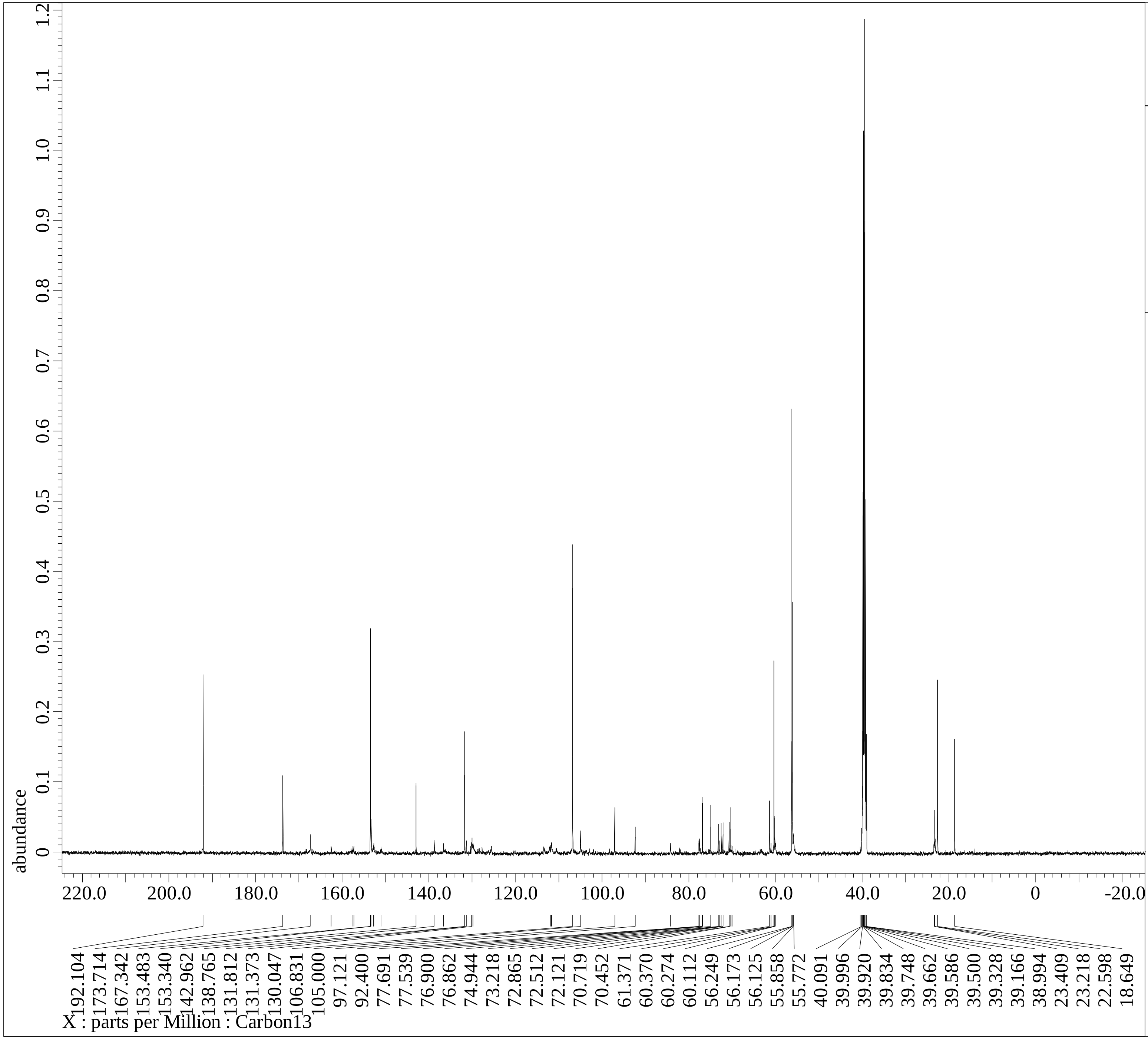

JEOL RESONANCE

```
---- PROCESSING PARAMETERS ----
dc_balance( 0, FALSE )
sexp( 2.0[Hz], 0.0[s] )
trapezoid( 0[%], 0[%], 80[%], 100[%] )
zerofill( 1 )
fft( 1, TRUE, TRUE )
machinephase
ppm
phase( 0.03871, -1.98176, 74.0396[%] )
phase( 0, -17.3864, 74.07393[%] )
reference( 39.84178[ppm], 39.5[ppm] )
```

```
Filename          = E6c_carbon-1-6.jdf
Author            = console
Experiment        = carbon.jxp
Sample_Id         = E6c
Solvent           = DMSO-D6
Creation_Time     =  7-DEC-2020 19:45:58
Revision_Time     = 10-DEC-2020 12:55:44
Current_Time      = 10-DEC-2020 12:55:48

Comment           = single pulse decoupled gat
Data_Format       = 1D COMPLEX
Dim_Size          = 26214
Dim_Title         = Carbon13
Dim_Units         = [ppm]
Dimensions        = X
Site              = JNM-ECX500II
Spectrometer      = DELTA2_NMR

Field_Strength    = 11.7473579[T] (500[MHz])
X_Acq_Duration    = 0.83361792[s]
X_Domain          = 13C
X_Freq            = 125.76529768[MHz]
X_Offset          = 100[ppm]
X_Points          = 32768
X_Prescans        = 4
X_Resolution      = 1.19959034[Hz]
X_Sweep           = 39.3081761[kHz]
X_Sweep_Clipped   = 31.44654088[kHz]
Irr_Domain        = Proton
Irr_Freq          = 500.15991521[MHz]
Irr_Offset        = 5.0[ppm]
Clipped           = FALSE
Scans             = 1024
Total_Scans       = 1024

Relaxation_Delay  = 2[s]
Recvr_Gain        = 50
Temp_Get          = 21.9[dC]
X_90_Width        = 10[us]
X_Acq_Time        = 0.83361792[s]
X_Angle           = 30[deg]
X_Atn             = 12.1[dB]
X_Pulse           = 3.33333333[us]
Irr_Atn_Dec       = 25.569[dB]
Irr_Atn_Noe       = 25.569[dB]
Irr_Noise         = WALTZ
Irr_Pwidth        = 92[us]
Decoupling        = TRUE
```

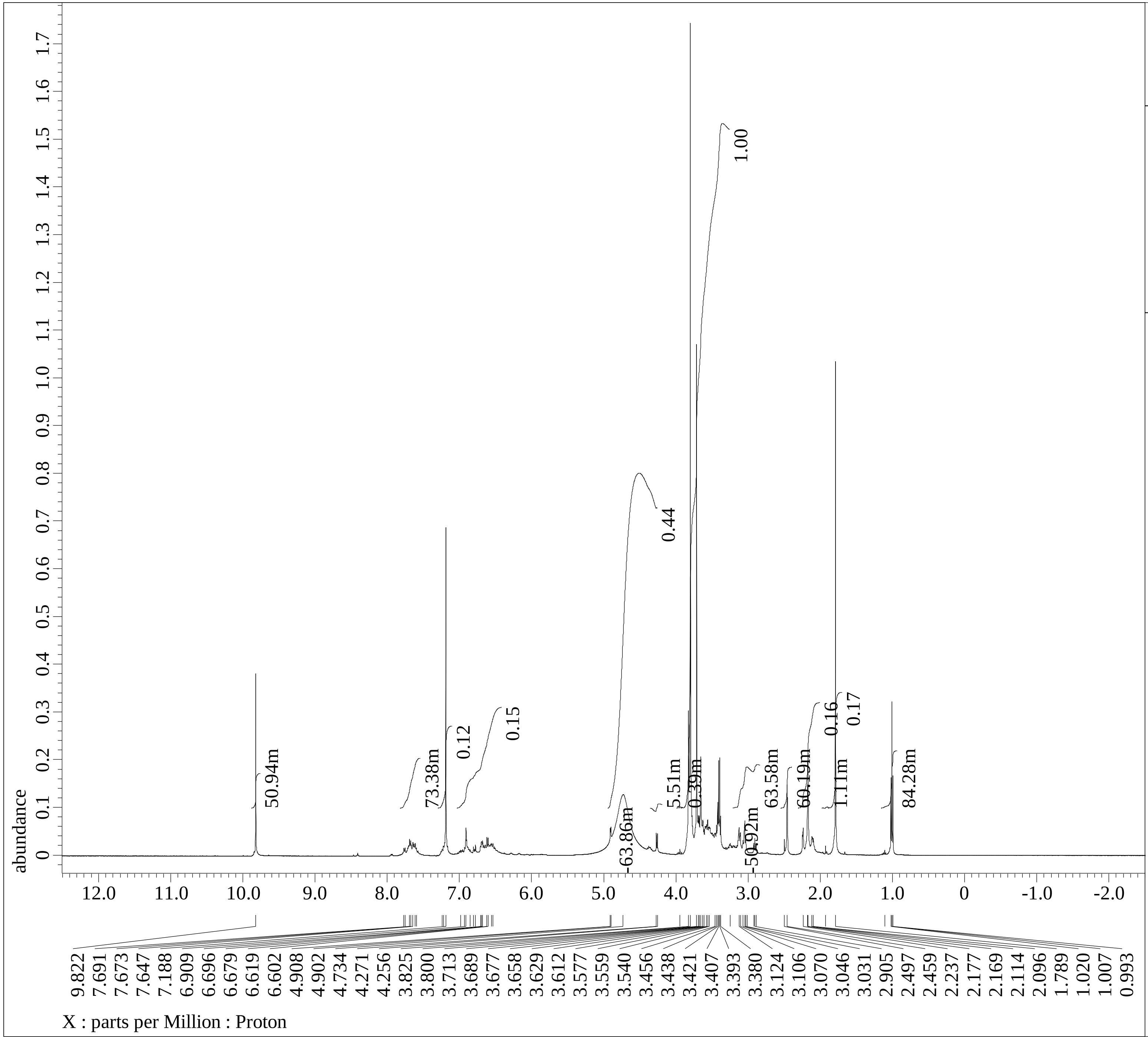

JEOL RESONANCE

```
---- PROCESSING PARAMETERS ----
dc_balance( 0, FALSE )
sexp( 0.2[Hz], 0.0[s] )
trapezoid( 0[%], 0[%], 80[%], 100[%] )
zerofill( 1 )
fft( 1, TRUE, TRUE )
machinephase
ppm
phase( -0.71625, 20.93286, 57.859[%] )
phase( -3.19703, -14.41646, 57.95819[%] )

Derived from: E6c_proton-1-1.jdf

Filename          = E6c_proton-1-4.jdf
Author            = console
Experiment        = proton.jxp
Sample_Id         = E6c
Solvent           = DMSO-D6
Creation_Time     =  7-DEC-2020 19:42:25
Revision_Time     = 10-DEC-2020 12:54:46
Current_Time      = 10-DEC-2020 12:54:53

Comment           = single_pulse
Data_Format       = 1D COMPLEX
Dim_Size          = 13107
Dim_Title         = Proton
Dim_Units         = [ppm]
Dimensions        = X
Site              = JNM-ECX500II
Spectrometer      = DELTA2_NMR

Field_Strength    = 11.7473579[T] (500[MHz])
X_Acq_Duration    = 1.74587904[s]
X_Domain          = 1H
X_Freq            = 500.15991521[MHz]
X_Offset          = 5.0[ppm]
X_Points          = 16384
X_Prescans        = 1
X_Resolution      = 0.57277737[Hz]
X_Sweep           = 9.38438438[kHz]
X_Sweep_Clipped   = 7.50750751[kHz]
Irr_Domain        = Proton
Irr_Freq          = 500.15991521[MHz]
Irr_Offset        = 5.0[ppm]
Tri_Domain        = Proton
Tri_Freq          = 500.15991521[MHz]
Tri_Offset        = 5.0[ppm]
Clipped           = FALSE
Scans             = 8
Total_Scans       = 8

Relaxation_Delay  = 5[s]
Recvr_Gain        = 10
Temp_Get          = 21.8[dC]
X_90_Width        = 7.25[us]
X_Acq_Time        = 1.74587904[s]
X_Angle           = 45[deg]
X_Atn             = 3.5[dB]
X_Pulse           = 3.625[us]
Irr_Mode          = Off
Tri_Mode          = Off
```

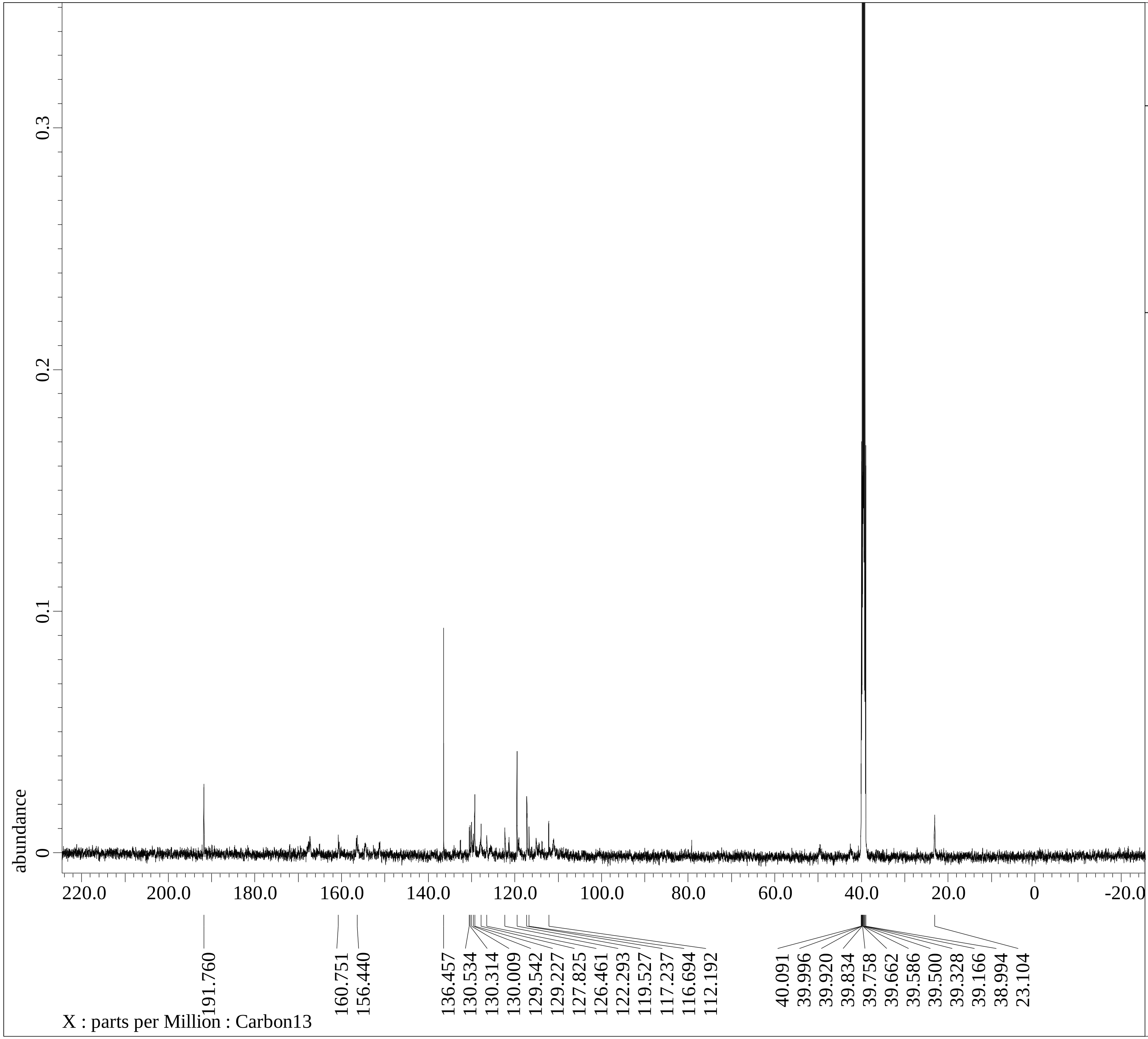


```
---- PROCESSING PARAMETERS ----
dc_balance( 0, FALSE )
sexp( 2.0[Hz], 0.0[s] )
trapezoid( 0[%], 0[%], 80[%], 100[%] )
zerofill( 1 )
fft( 1, TRUE, TRUE )
machinephase
ppm
phase( 0.1591, 0.00651, 74.00908[%] )
reference( 40.00393[ppm], 39.5[ppm] )
```

```
Filename          = E6d_carbon-1-5.jdf
Author            = console
Experiment        = carbon.jxp
Sample_Id         = E6d
Solvent           = DMSO-D6
Creation_Time     =  8-DEC-2020 09:34:11
Revision_Time     = 10-DEC-2020 12:57:45
Current_Time      = 10-DEC-2020 12:57:53

Comment           = single pulse decoupled gat
Data_Format       = 1D COMPLEX
Dim_Size          = 26214
Dim_Title         = Carbon13
Dim_Units         = [ppm]
Dimensions        = X
Site              = JNM-ECX500II
Spectrometer      = DELTA2_NMR

Field_Strength    = 11.7473579[T] (500[MHz])
X_Acq_Duration    = 0.83361792[s]
X_Domain          = 13C
X_Freq            = 125.76529768[MHz]
X_Offset          = 100[ppm]
X_Points          = 32768
X_Prescans        = 4
X_Resolution      = 1.19959034[Hz]
X_Sweep           = 39.3081761[kHz]
X_Sweep_Clipped   = 31.44654088[kHz]
Irr_Domain        = Proton
Irr_Freq          = 500.15991521[MHz]
Irr_Offset        = 5.0[ppm]
Clipped           = FALSE
Scans             = 1024
Total_Scans       = 1024

Relaxation_Delay  = 2[s]
Recvr_Gain        = 50
Temp_Get          = 22[dC]
X_90_Width        = 10[us]
X_Acq_Time        = 0.83361792[s]
X_Angle           = 30[deg]
X_Atn             = 12.1[dB]
X_Pulse           = 3.33333333[us]
Irr_Atn_Dec       = 25.569[dB]
Irr_Atn_Noe       = 25.569[dB]
Irr_Noise         = WALTZ
Irr_Pwidth        = 92[us]
Decoupling        = TRUE
```

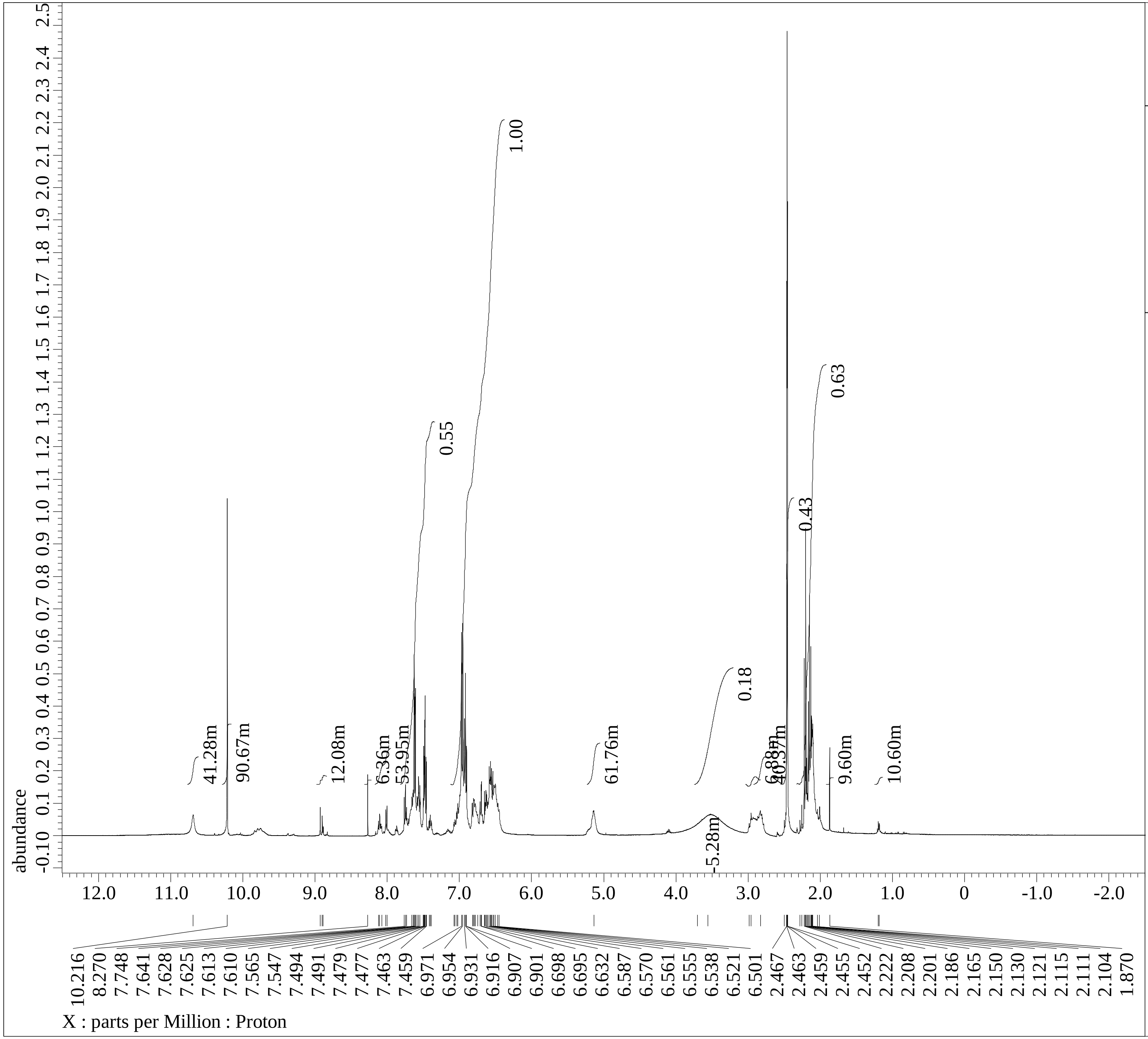

JEOL RESONANCE

```
---- PROCESSING PARAMETERS ----
dc_balance( 0, FALSE )
sexp( 0.2[Hz], 0.0[s] )
trapezoid( 0[%], 0[%], 80[%], 100[%] )
zerofill( 1 )
fft( 1, TRUE, TRUE )
machinephase
ppm
phase( 14.04862, -85.09707, 66.87014[%] )
phase( -10.75411, 66.08916, 66.99222[%] )

Derived from: E6d_proton-1-1.jdf
```

```
Filename          = E6d_proton-1-4.jdf
Author            = console
Experiment        = proton.jxp
Sample_Id         = E6d
Solvent           = DMSO-D6
Creation_Time     =  8-DEC-2020 09:30:41
Revision_Time     = 10-DEC-2020 12:57:01
Current_Time      = 10-DEC-2020 12:57:10

Comment           = single_pulse
Data_Format       = 1D COMPLEX
Dim_Size          = 13107
Dim_Title         = Proton
Dim_Units         = [ppm]
Dimensions        = X
Site              = JNM-ECX500II
Spectrometer      = DELTA2_NMR

Field_Strength    = 11.7473579[T] (500[MHz])
X_Acq_Duration    = 1.74587904[s]
X_Domain          = 1H
X_Freq            = 500.15991521[MHz]
X_Offset          = 5.0[ppm]
X_Points          = 16384
X_Prescans        = 1
X_Resolution      = 0.57277737[Hz]
X_Sweep           = 9.38438438[kHz]
X_Sweep_Clipped   = 7.50750751[kHz]
Irr_Domain        = Proton
Irr_Freq          = 500.15991521[MHz]
Irr_Offset        = 5.0[ppm]
Tri_Domain        = Proton
Tri_Freq          = 500.15991521[MHz]
Tri_Offset        = 5.0[ppm]
Clipped           = FALSE
Scans             = 8
Total_Scans       = 8

Relaxation_Delay  = 5[s]
Recvr_Gain        = 30
Temp_Get          = 21.9[dC]
X_90_Width        = 7.25[us]
X_Acq_Time        = 1.74587904[s]
X_Angle           = 45[deg]
X_Atn             = 3.5[dB]
X_Pulse           = 3.625[us]
Irr_Mode          = Off
Tri_Mode          = Off
```

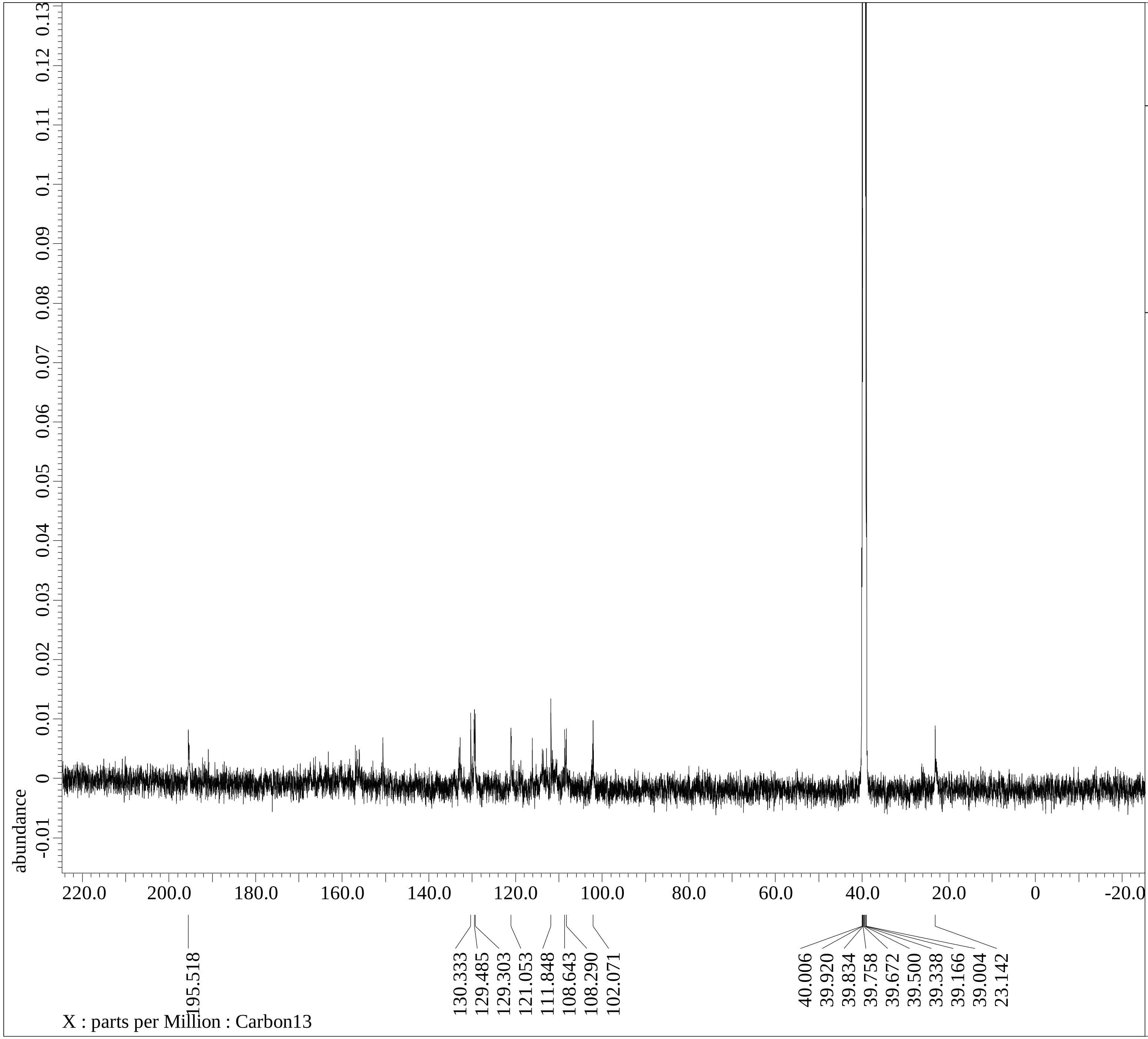


```
---- PROCESSING PARAMETERS ----
dc_balance( 0, FALSE )
sexp( 2.0[Hz], 0.0[s] )
trapezoid( 0[%], 0[%], 80[%], 100[%] )
zerofill( 1 )
fft( 1, TRUE, TRUE )
machinephase
ppm
phase( 0.06585, 0, 73.97475[%] )
reference( 40.00393[ppm], 39.5[ppm] )
```

```
Filename          = E6e_carbon-1-5.jdf
Author            = console
Experiment        = carbon.jxp
Sample_Id         = E6e
Solvent           = DMSO-D6
Creation_Time     =  8-DEC-2020 10:31:21
Revision_Time     = 10-DEC-2020 13:01:51
Current_Time      = 10-DEC-2020 13:02:05

Comment           = single pulse decoupled gat
Data_Format       = 1D COMPLEX
Dim_Size          = 26214
Dim_Title         = Carbon13
Dim_Units         = [ppm]
Dimensions        = X
Site              = JNM-ECX500II
Spectrometer      = DELTA2_NMR

Field_Strength    = 11.7473579[T] (500[MHz])
X_Acq_Duration    = 0.83361792[s]
X_Domain          = 13C
X_Freq            = 125.76529768[MHz]
X_Offset          = 100[ppm]
X_Points          = 32768
X_Prescans        = 4
X_Resolution      = 1.19959034[Hz]
X_Sweep           = 39.3081761[kHz]
X_Sweep_Clipped   = 31.44654088[kHz]
Irr_Domain        = Proton
Irr_Freq          = 500.15991521[MHz]
Irr_Offset        = 5.0[ppm]
Clipped           = FALSE
Scans             = 1024
Total_Scans       = 1024

Relaxation_Delay  = 2[s]
Recvr_Gain        = 50
Temp_Get          = 22[dC]
X_90_Width        = 10[us]
X_Acq_Time        = 0.83361792[s]
X_Angle           = 30[deg]
X_Atn             = 12.1[dB]
X_Pulse           = 3.33333333[us]
Irr_Atn_Dec       = 25.569[dB]
Irr_Atn_Noe       = 25.569[dB]
Irr_Noise         = WALTZ
Irr_Pwidth        = 92[us]
Decoupling        = TRUE
```

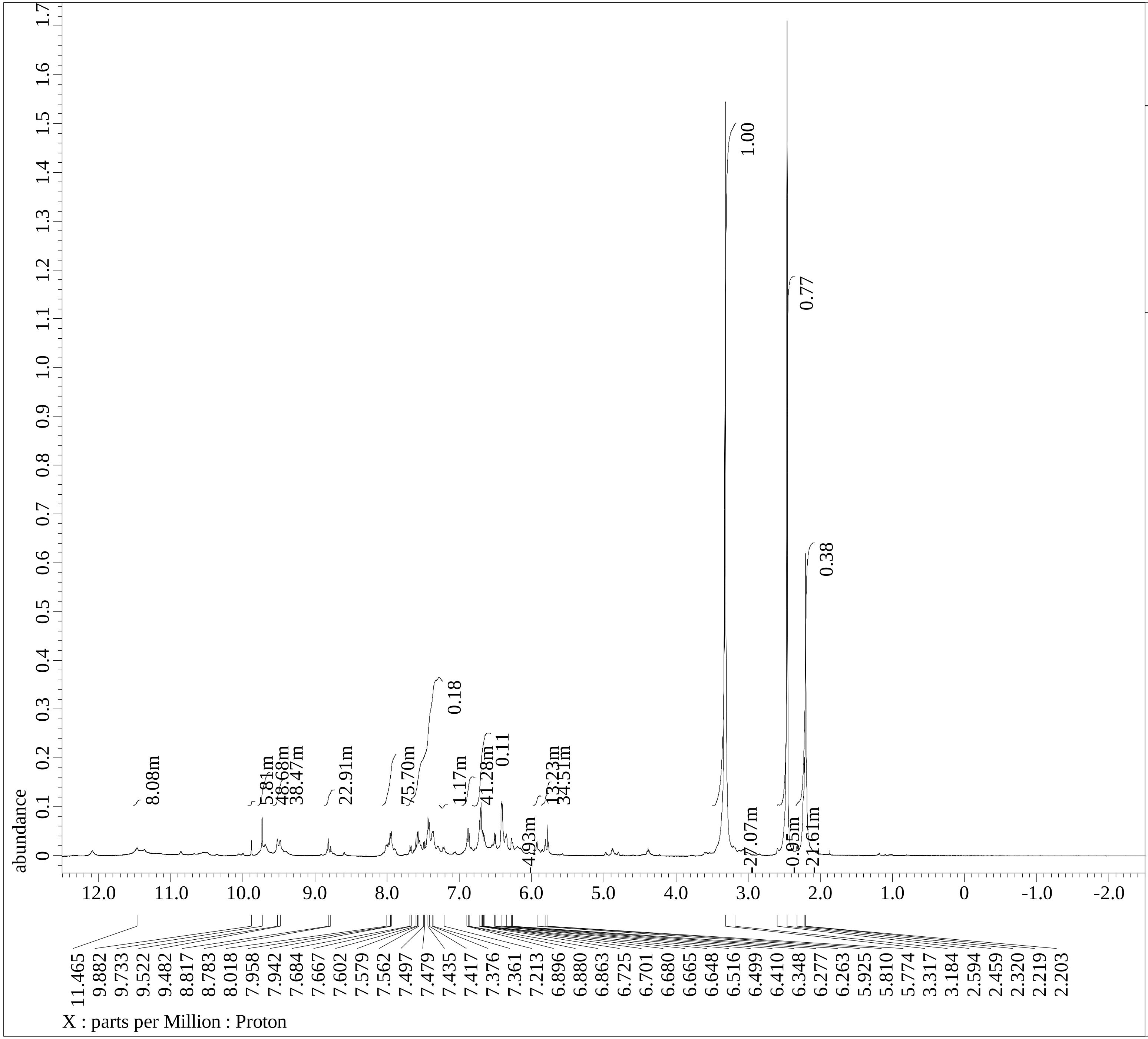

JEOL RESONANCE

```
---- PROCESSING PARAMETERS ----
dc_balance( 0, FALSE )
sexp( 0.2[Hz], 0.0[s] )
trapezoid( 0[%], 0[%], 80[%], 100[%] )
zerofill( 1 )
fft( 1, TRUE, TRUE )
machinephase
ppm
phase( 0.34606, -130.83562, 66.93881[%] )
phase( -1.13606, 134.68352, 66.92355[%] )

Derived from: E6e_proton-1-1.jdf
```

```
Filename          = E6e_proton-1-4.jdf
Author            = console
Experiment        = proton.jxp
Sample_Id         = E6e
Solvent           = DMSO-D6
Creation_Time     =  8-DEC-2020 08:59:43
Revision_Time     = 10-DEC-2020 13:00:25
Current_Time      = 10-DEC-2020 13:00:55

Comment           = single_pulse
Data_Format       = 1D COMPLEX
Dim_Size          = 13107
Dim_Title         = Proton
Dim_Units         = [ppm]
Dimensions        = X
Site              = JNM-ECX500II
Spectrometer      = DELTA2_NMR

Field_Strength    = 11.7473579[T] (500[MHz])
X_Acq_Duration    = 1.74587904[s]
X_Domain          = 1H
X_Freq            = 500.15991521[MHz]
X_Offset          = 5.0[ppm]
X_Points          = 16384
X_Prescans        = 1
X_Resolution      = 0.57277737[Hz]
X_Sweep           = 9.38438438[kHz]
X_Sweep_Clipped   = 7.50750751[kHz]
Irr_Domain        = Proton
Irr_Freq          = 500.15991521[MHz]
Irr_Offset        = 5.0[ppm]
Tri_Domain        = Proton
Tri_Freq          = 500.15991521[MHz]
Tri_Offset        = 5.0[ppm]
Clipped           = FALSE
Scans             = 8
Total_Scans       = 8

Relaxation_Delay  = 5[s]
Recvr_Gain        = 30
Temp_Get          = 21.9[dC]
X_90_Width        = 7.25[us]
X_Acq_Time        = 1.74587904[s]
X_Angle           = 45[deg]
X_Atn             = 3.5[dB]
X_Pulse           = 3.625[us]
Irr_Mode          = Off
Tri_Mode          = Off
```

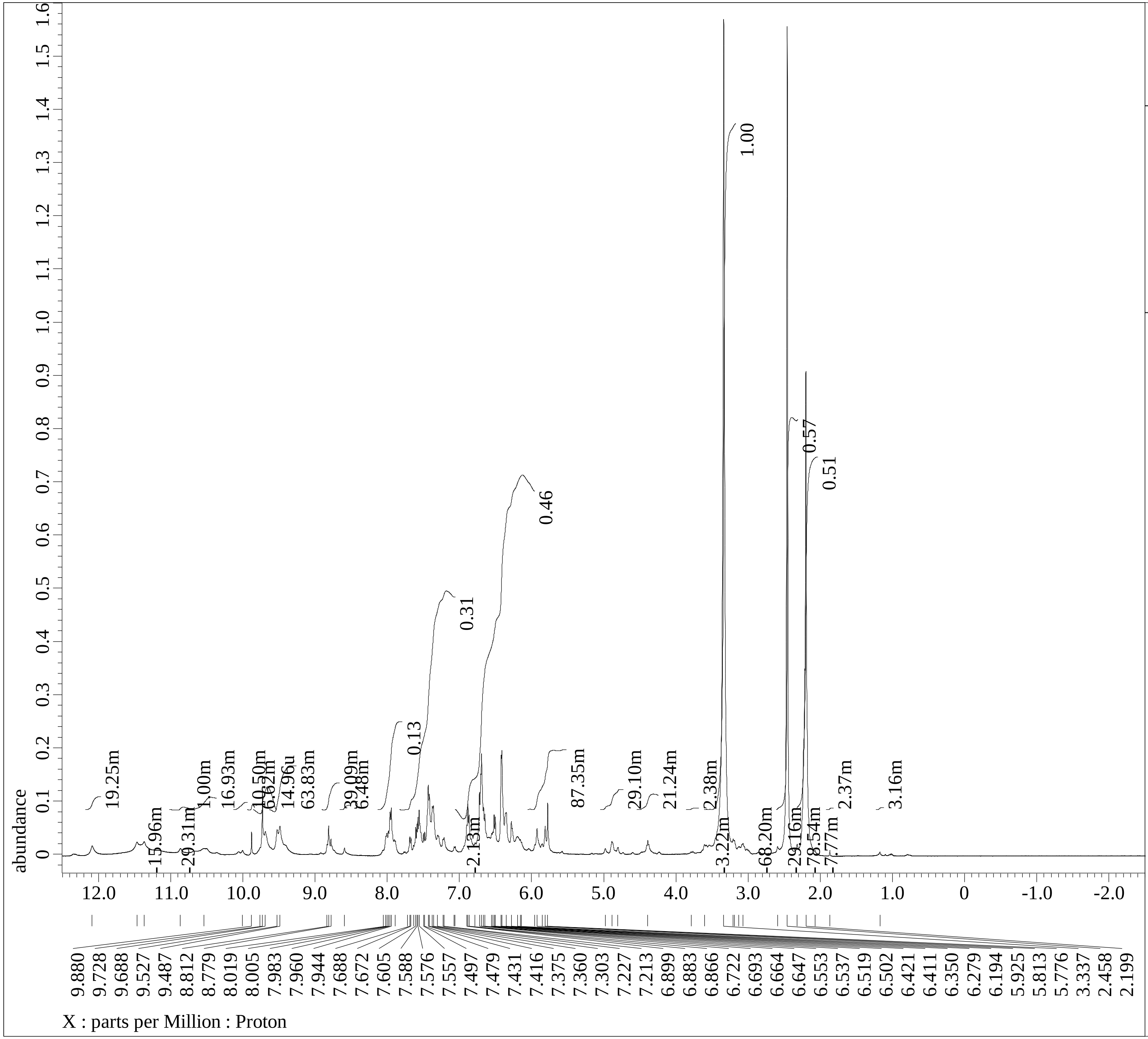

JEOL RESONANCE

---- PROCESSING PARAMETERS ----
dc_balance(0, FALSE)
sexp(0.2[Hz], 0.0[s])
trapezoid(0[%], 0[%], 80[%], 100[%])
zerofill(1)
fft(1, TRUE, TRUE)
machinephase
ppm
phase(-3.22753, -97.86927, 61.08653[%])
phase(-0.6665, 112.05262, 61.01022[%])

Derived from: E6e_proton-2-1.jdf

Filename = E6e_proton-2-4.jdf
Author = console
Experiment = proton.jxp
Sample_Id = E6e
Solvent = DMSO-D6
Creation_Time = 8-DEC-2020 10:27:51
Revision_Time = 10-DEC-2020 13:03:29
Current_Time = 10-DEC-2020 13:03:37

Comment = single_pulse
Data_Format = 1D COMPLEX
Dim_Size = 13107
Dim_Title = Proton
Dim_Units = [ppm]
Dimensions = X
Site = JNM-ECX500II
Spectrometer = DELTA2_NMR

Field_Strength = 11.7473579[T] (500[MHz])
X_Acq_Duration = 1.74587904[s]
X_Domain = 1H
X_Freq = 500.15991521[MHz]
X_Offset = 5.0[ppm]
X_Points = 16384
X_Prescans = 1
X_Resolution = 0.57277737[Hz]
X_Sweep = 9.38438438[kHz]
X_Sweep_Clipped = 7.50750751[kHz]
Irr_Domain = Proton
Irr_Freq = 500.15991521[MHz]
Irr_Offset = 5.0[ppm]
Tri_Domain = Proton
Tri_Freq = 500.15991521[MHz]
Tri_Offset = 5.0[ppm]
Clipped = FALSE
Scans = 8
Total_Scans = 8

Relaxation_Delay = 5[s]
Recvr_Gain = 30
Temp_Get = 22[dC]
X_90_Width = 7.25[us]
X_Acq_Time = 1.74587904[s]
X_Angle = 45[deg]
X_Atn = 3.5[dB]
X_Pulse = 3.625[us]
Irr_Mode = Off
Tri_Mode = Off

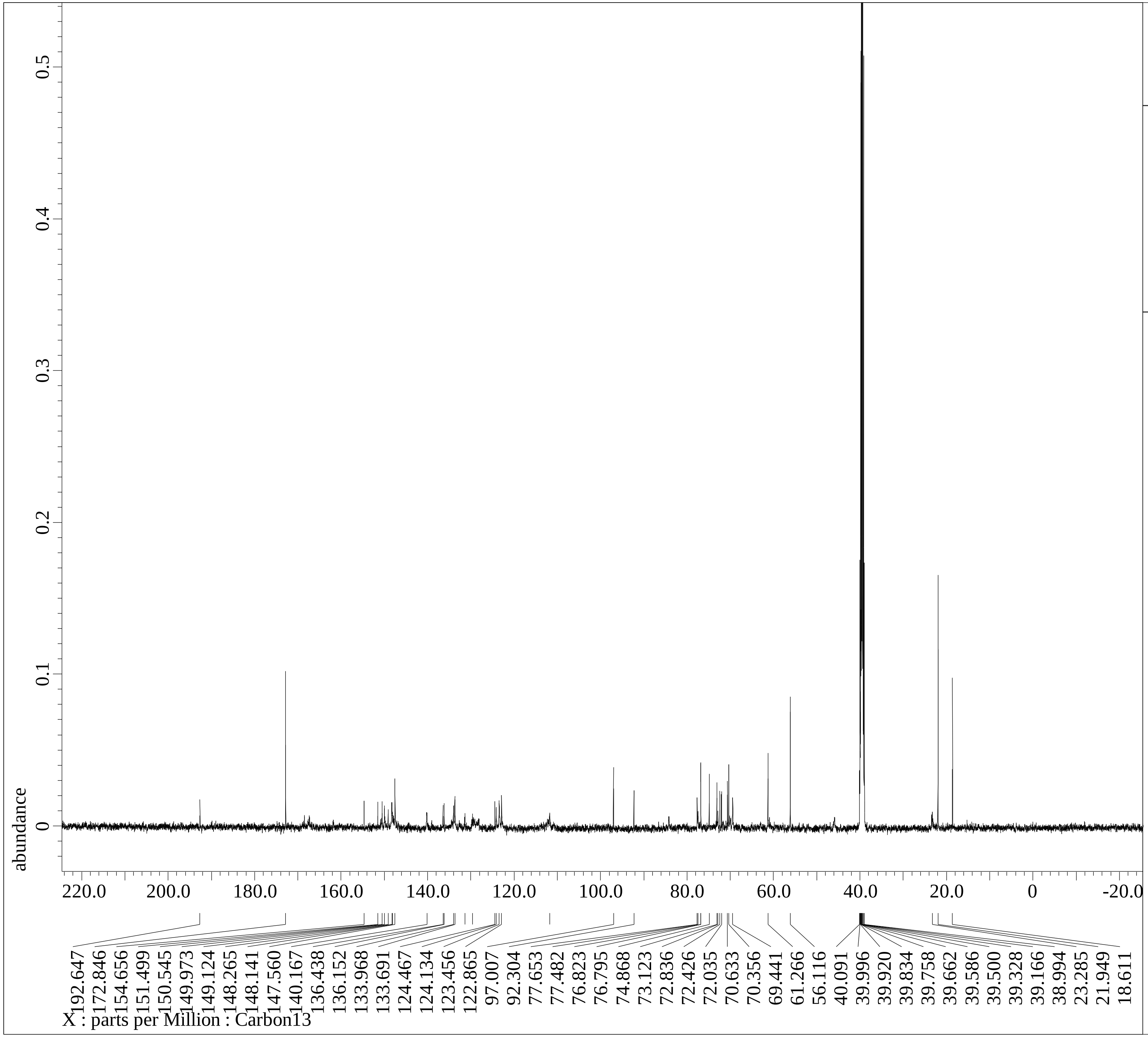

JEOL RESONANCE

```
---- PROCESSING PARAMETERS ----
dc_balance( 0, FALSE )
sexp( 2.0[Hz], 0.0[s] )
trapezoid( 0[%], 0[%], 80[%], 100[%] )
zerofill( 1 )
fft( 1, TRUE, TRUE )
machinephase
ppm
phase( 0.00765, -21.524, 73.99382[%] )
reference( 39.95624[ppm], 39.5[ppm] )
```

```
Filename          = E6f_carbon-1-5.jdf
Author            = console
Experiment        = carbon.jxp
Sample_Id         = E6f
Solvent           = DMSO-D6
Creation_Time     =  8-DEC-2020 11:27:43
Revision_Time     = 10-DEC-2020 13:05:32
Current_Time      = 10-DEC-2020 13:05:40

Comment           = single pulse decoupled gat
Data_Format       = 1D COMPLEX
Dim_Size          = 26214
Dim_Title         = Carbon13
Dim_Units         = [ppm]
Dimensions        = X
Site              = JNM-ECX500II
Spectrometer      = DELTA2_NMR

Field_Strength    = 11.7473579[T] (500[MHz])
X_Acq_Duration    = 0.83361792[s]
X_Domain          = 13C
X_Freq            = 125.76529768[MHz]
X_Offset          = 100[ppm]
X_Points          = 32768
X_Prescans        = 4
X_Resolution      = 1.19959034[Hz]
X_Sweep           = 39.3081761[kHz]
X_Sweep_Clipped   = 31.44654088[kHz]
Irr_Domain        = Proton
Irr_Freq          = 500.15991521[MHz]
Irr_Offset        = 5.0[ppm]
Clipped           = FALSE
Scans             = 1024
Total_Scans       = 1024

Relaxation_Delay  = 2[s]
Recvr_Gain        = 50
Temp_Get          = 22[dC]
X_90_Width        = 10[us]
X_Acq_Time        = 0.83361792[s]
X_Angle           = 30[deg]
X_Atn             = 12.1[dB]
X_Pulse           = 3.33333333[us]
Irr_Atn_Dec       = 25.569[dB]
Irr_Atn_Noe       = 25.569[dB]
Irr_Noise         = WALTZ
Irr_Pwidth        = 92[us]
Decoupling        = TRUE
```

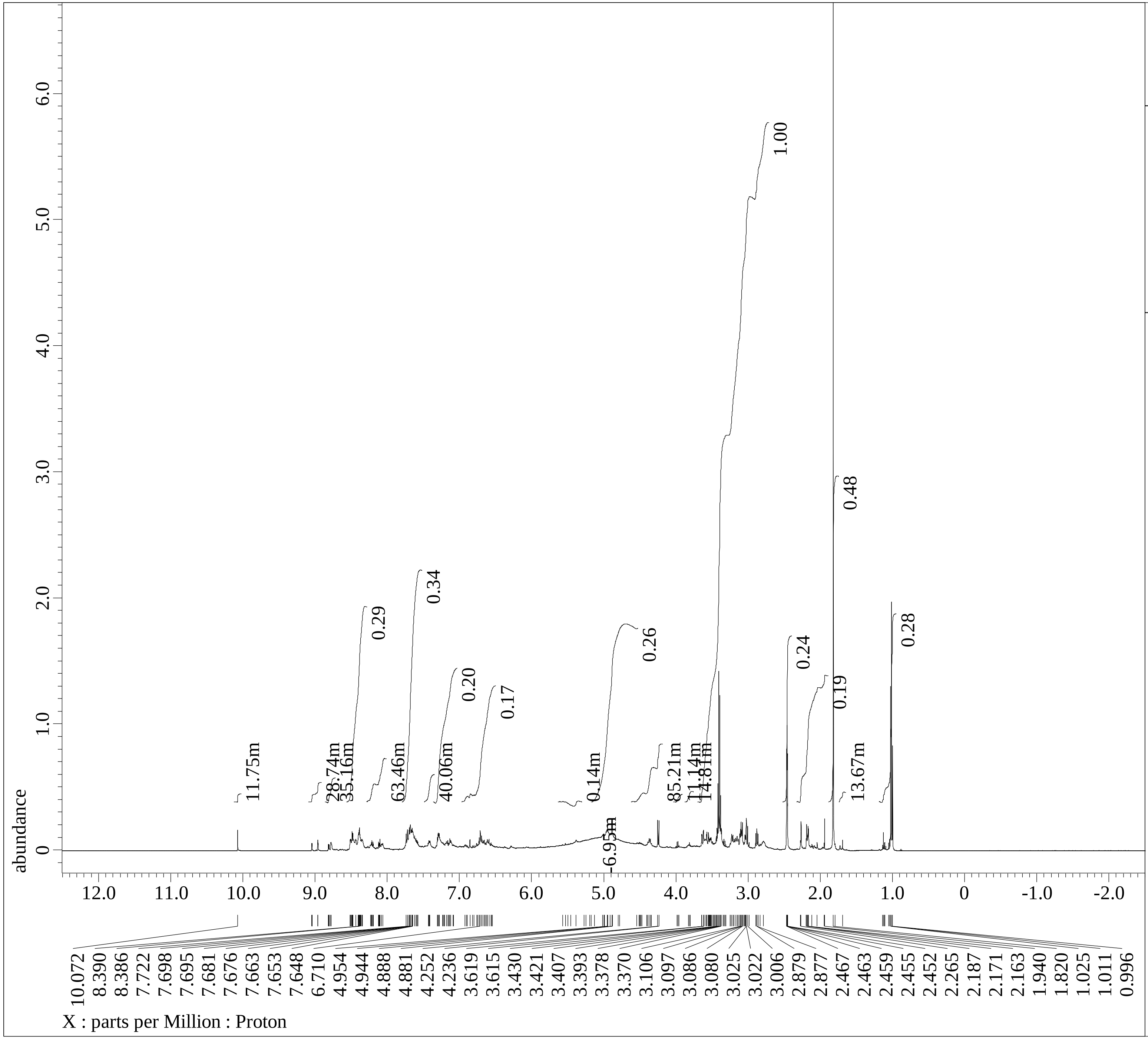

JEOL RESONANCE

```
---- PROCESSING PARAMETERS ----
dc_balance( 0, FALSE )
sexp( 0.2[Hz], 0.0[s] )
trapezoid( 0[%], 0[%], 80[%], 100[%] )
zerofill( 1 )
fft( 1, TRUE, TRUE )
machinephase
ppm
phase( 8.26697, -51.58882, 71.26507[%] )
phase( -20.17963, 68.97793, 71.15062[%] )

Derived from: E6f_proton-1-1.jdf

Filename          = E6f_proton-1-5.jdf
Author            = console
Experiment        = proton.jxp
Sample_Id         = E6f
Solvent           = DMSO-D6
Creation_Time     =  8-DEC-2020 11:24:14
Revision_Time     = 10-DEC-2020 13:04:46
Current_Time      = 10-DEC-2020 13:04:50

Comment           = single_pulse
Data_Format       = 1D COMPLEX
Dim_Size          = 13107
Dim_Title         = Proton
Dim_Units         = [ppm]
Dimensions        = X
Site              = JNM-ECX500II
Spectrometer      = DELTA2_NMR

Field_Strength    = 11.7473579[T] (500[MHz])
X_Acq_Duration    = 1.74587904[s]
X_Domain          = 1H
X_Freq            = 500.15991521[MHz]
X_Offset          = 5.0[ppm]
X_Points          = 16384
X_Prescans        = 1
X_Resolution      = 0.57277737[Hz]
X_Sweep           = 9.38438438[kHz]
X_Sweep_Clipped   = 7.50750751[kHz]
Irr_Domain        = Proton
Irr_Freq          = 500.15991521[MHz]
Irr_Offset        = 5.0[ppm]
Tri_Domain        = Proton
Tri_Freq          = 500.15991521[MHz]
Tri_Offset        = 5.0[ppm]
Clipped           = FALSE
Scans             = 8
Total_Scans       = 8

Relaxation_Delay  = 5[s]
Recvr_Gain        = 26
Temp_Get          = 22[dC]
X_90_Width        = 7.25[us]
X_Acq_Time        = 1.74587904[s]
X_Angle           = 45[deg]
X_Atn             = 3.5[dB]
X_Pulse           = 3.625[us]
Irr_Mode          = Off
Tri_Mode          = Off
```

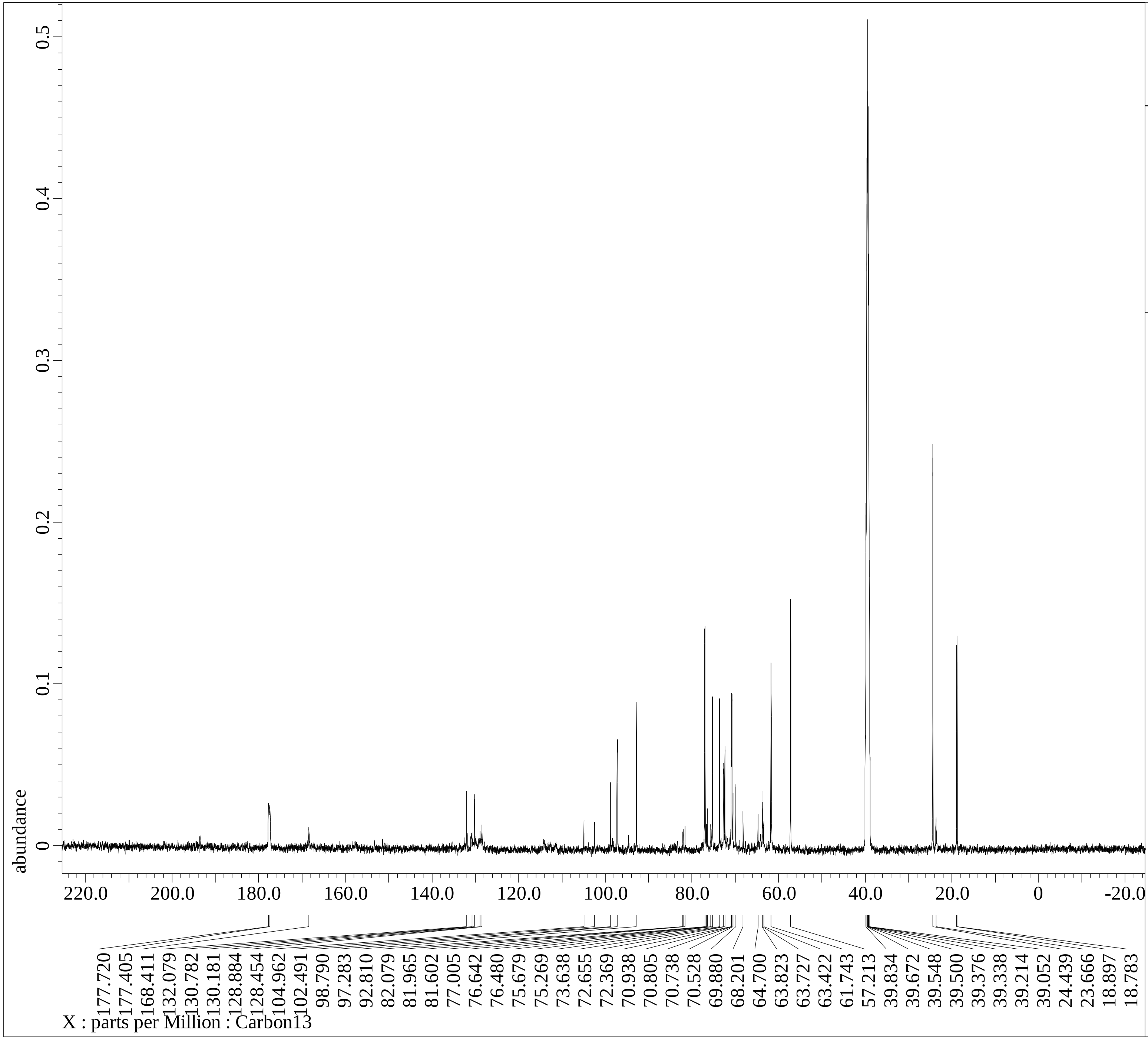


```
---- PROCESSING PARAMETERS ----
dc_balance( 0, FALSE )
sexp( 2.0[Hz], 0.0[s] )
trapezoid( 0[%], 0[%], 80[%], 100[%] )
zerofill( 1 )
fft( 1, TRUE, TRUE )
machinephase
ppm
phase( 0.35766, -19.83141, 74.36768[%] )
phase( 0, 4.47518, 74.33335[%] )
reference( 39.12641[ppm], 39.5[ppm] )

Filename          = E6g_carbon-1-6.jdf
Author            = console
Experiment        = carbon.jxp
Sample_Id         = E6g
Solvent           = DMSO-D6
Creation_Time     =  8-DEC-2020 12:23:56
Revision_Time     = 10-DEC-2020 13:07:48
Current_Time      = 10-DEC-2020 13:07:52

Comment           = single pulse decoupled gat
Data_Format       = 1D COMPLEX
Dim_Size          = 26214
Dim_Title         = Carbon13
Dim_Units         = [ppm]
Dimensions        = X
Site              = JNM-ECX500II
Spectrometer      = DELTA2_NMR

Field_Strength    = 11.7473579[T] (500[MHz])
X_Acq_Duration    = 0.83361792[s]
X_Domain          = 13C
X_Freq            = 125.76529768[MHz]
X_Offset          = 100[ppm]
X_Points          = 32768
X_Prescans        = 4
X_Resolution      = 1.19959034[Hz]
X_Sweep           = 39.3081761[kHz]
X_Sweep_Clipped   = 31.44654088[kHz]
Irr_Domain        = Proton
Irr_Freq          = 500.15991521[MHz]
Irr_Offset        = 5.0[ppm]
Clipped           = FALSE
Scans             = 1024
Total_Scans       = 1024

Relaxation_Delay  = 2[s]
Recvr_Gain        = 50
Temp_Get          = 22.1[dC]
X_90_Width        = 10[us]
X_Acq_Time        = 0.83361792[s]
X_Angle           = 30[deg]
X_Atn             = 12.1[dB]
X_Pulse           = 3.33333333[us]
Irr_Atn_Dec       = 25.569[dB]
Irr_Atn_Noe       = 25.569[dB]
Irr_Noise         = WALTZ
Irr_Pwidth        = 92[us]
Decoupling        = TRUE
```

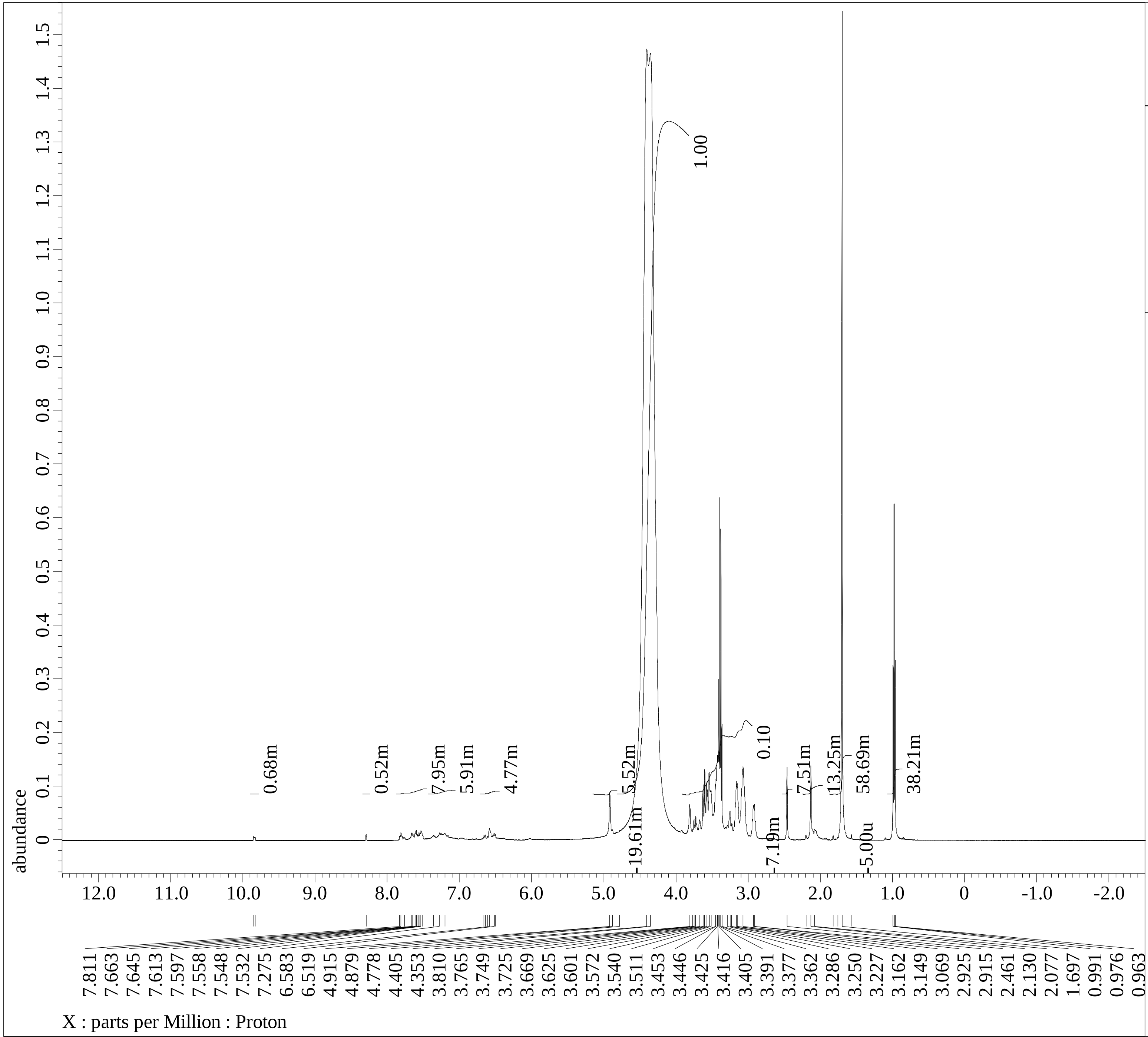


```
---- PROCESSING PARAMETERS ----
dc_balance( 0, FALSE )
sexp( 0.2[Hz], 0.0[s] )
trapezoid( 0[%], 0[%], 80[%], 100[%] )
zerofill( 1 )
fft( 1, TRUE, TRUE )
machinephase
ppm
phase( -10.68739, 59.77527, 72.06623[%] )
phase( 1.4372, -70.03315, 54.31863[%] )

Derived from: E6g_proton-1-1.jdf

Filename          = E6g_proton-1-4.jdf
Author            = console
Experiment        = proton.jxp
Sample_Id         = E6g
Solvent           = DMSO-D6
Creation_Time     =  8-DEC-2020 12:20:28
Revision_Time     = 10-DEC-2020 13:06:49
Current_Time      = 10-DEC-2020 13:06:56

Comment           = single_pulse
Data_Format       = 1D COMPLEX
Dim_Size          = 13107
Dim_Title         = Proton
Dim_Units         = [ppm]
Dimensions        = X
Site              = JNM-ECX500II
Spectrometer      = DELTA2_NMR

Field_Strength    = 11.7473579[T] (500[MHz])
X_Acq_Duration    = 1.74587904[s]
X_Domain          = 1H
X_Freq            = 500.15991521[MHz]
X_Offset          = 5.0[ppm]
X_Points          = 16384
X_Prescans        = 1
X_Resolution      = 0.57277737[Hz]
X_Sweep           = 9.38438438[kHz]
X_Sweep_Clipped   = 7.50750751[kHz]
Irr_Domain        = Proton
Irr_Freq          = 500.15991521[MHz]
Irr_Offset        = 5.0[ppm]
Tri_Domain        = Proton
Tri_Freq          = 500.15991521[MHz]
Tri_Offset        = 5.0[ppm]
Clipped           = FALSE
Scans             = 8
Total_Scans       = 8

Relaxation_Delay  = 5[s]
Recvr_Gain        = 10
Temp_Get          = 22[dC]
X_90_Width        = 7.25[us]
X_Acq_Time        = 1.74587904[s]
X_Angle           = 45[deg]
X_Atn             = 3.5[dB]
X_Pulse           = 3.625[us]
Irr_Mode          = Off
Tri_Mode          = Off
```
